# What Motives Do People Most Want to Know About When Meeting Another Person? An Investigation Into Prioritization of Information About Seven Fundamental Motives

**DOI:** 10.1177/01461672211069468

**Published:** 2022-01-26

**Authors:** Matthew I. Billet, Hugh C. McCall, Mark Schaller

**Affiliations:** 1 University of British Columbia, Vancouver, Canada; 2 University of Regina, Saskatchewan, Canada

**Keywords:** fundamental social motives, motivation, person perception, social cognition

## Abstract

What information about a person’s personality do people want to know? Prior research has focused on behavioral *traits*, but personality is also characterized in terms of *motives*. Four studies (*N* = 1,502) assessed participants’ interest in information about seven fundamental social motives (self-protection, disease avoidance, affiliation, status, mate seeking, mate retention, kin care) across 12 experimental conditions that presented details about the person or situation. In the absence of details about specific situations, participants most highly prioritized learning about kin care and mate retention motives. There was some variability across conditions, but the kin care motive was consistently highly prioritized. Additional results from Studies 1 to 4 and Study 5 (*N* = 174) showed the most highly prioritized motives were perceived to be stable across time and to be especially diagnostic of a person’s trustworthiness, warmth, competence, and dependability. Findings are discussed in relation to research on fundamental social motives and pragmatic perspectives on person perception.

What information about a person’s personality do people most want to know? Many studies have assessed the personality traits that people prioritize obtaining information about, and results converge upon a common conclusion: People want to know about traits that connote whether a person’s intentions are helpful or harmful (i.e., behavioral propensities for interpersonal warmth, communion, and “getting along”; Abele et al., 2021) and about traits that connote whether that person has the capacity to carry out those intentions (i.e., propensities for competence, agency, and “getting ahead”; Abele et al., 2021). Additional studies indicate that people may especially value information about the smaller subset of personality traits—such as trustworthiness, compassion, and dependability—that connote a person’s moral character (Cottrell et al., 2007; Goodwin et al., 2014; Hartley et al., 2016; Wortman & Wood, 2011). Based on all this evidence, it appears that the question posed earlier has been amply answered.

Or has it? That body of evidence pertains specifically to personality *traits*. Traits—behavioral dispositions—represent just one of several ways of describing a person’s personality (McAdams, 1995). At a different level of analysis, personality can be described in terms of underlying *motives* (Murray, 1938; Winter et al., 1998). These are conceptually distinct and complementary constructs: “motives involve wishes, desires, or goals (often implicit or nonconscious), whereas traits channel or direct the ways in which motives are expressed in particular actions” (Winter et al., 1998, p. 231). Just as it can be useful to know another person’s characteristic behavioral traits, so too it can be useful to know a person’s underlying enduring motives (which might potentially manifest across a wide range of specific behaviors). And, just as perceivers prioritize obtaining information about some traits—such as those that connote moral character—more than others, so too may they prioritize obtaining information about some motives more than others. If so, what motives might they most highly prioritize obtaining information about? And to what extent do these priorities vary across perceivers and social contexts? No previous research has systematically addressed these questions. The studies reported here represent one attempt to do so.

Any attempt to empirically address the prioritization of information about people’s motives must contend with the fact that there is no canonical set of motives that comprise a person’s personality. The psychological literature is replete with different lists of motives—and related concepts such as needs—that differ in length, content, and underlying conceptual rationale (e.g., Deci & Ryan, 2008; Kenrick et al., 2010; Maslow, 1943; McClelland, 1985; Murray, 1938). Because the research question here pertains not to personality, per se, but instead to person perception—which “operates in the service of social interaction” (Fiske, 1992, p. 877)—it may be sensible to direct empirical attention primarily toward motives that have clear consequences for social interaction. For this reason, we designed our studies to focus on the set of *fundamental social motives* identified in research by Kenrick and colleagues (Cook et al., 2021; Kenrick & Griskevicius, 2013; Kenrick et al., 2010; Ko et al., 2020; Krems et al., 2017; Neel et al., 2016; Schaller et al., 2017).

The fundamental social motives framework is grounded in a biological perspective that conceptualizes motives as functionally specialized psychological systems that evolved to regulate responses to threats and opportunities in organisms’ environments (Schaller et al., 2017; Tooby et al., 2008). Because humans are a highly social species, distinct motivational systems are adapted to distinct threats and opportunities posed by other people. Recent work within this framework (e.g., Brown et al., 2015; Huelsnitz et al., 2020; Ko et al., 2020; Krems et al., 2017; Morse et al., 2015; Neel et al., 2016; Neel & Lassetter, 2019; Pirlott & Cook, 2018) has focused especially on seven such “fundamental social motives”: *self-protection, disease avoidance, affiliation, status, mate seeking, mate retention*, and *kin care*.^1^

These seven motives cover a wide range of conceptual territory and—consistent with the purported “fundamental” nature of these motives—are associated with characteristic emotions (Beall & Tracy, 2017). The arousal of each motive is sensitive to immediate context (Morse et al., 2015; Schaller et al., 2017). However—consistent with the conceptualization of personality in terms of motives (Winter et al., 1998)—there are stable individual differences in the extent to which each motive is readily aroused. Some of these individual differences can be assessed with motive-specific self-report measures (e.g., Buckels et al., 2015; Duncan et al., 2009; Leary et al., 2013). In addition, Neel et al. (2016) developed a comprehensive questionnaire—the “Fundamental Social Motives Inventory”—to assess individual differences in all seven of the motives identified earlier. Results from studies validating that questionnaire revealed that these motives have unique explanatory and predictive utility (complementing the utility of trait measures) and supported the conclusion that this set of seven fundamental social motives represents “a powerful lens through which to examine individual differences. . .in social motivation” (Neel et al., 2016, p. 887).

Several recent lines of research have investigated perceivers’ subjective perceptions of these motives and their relevance to perceivers’ own actions and aspirations. One set of studies assessed the perceived relevance of these motives to Maslow’s (1943) concept of self-actualization (Krems et al., 2017). Results revealed that people perceived their pursuit of self-actualization to be governed most strongly by status and affiliation motives. Another study—for which data were obtained from thousands of people in 27 countries—assessed the extent to which people perceived each motive to be important within their own lives (Ko et al., 2020). Results revealed that, in virtually every country, people rated kin care and mate retention motives to be of greatest personal importance.

While the preceding results indicate the extent to which fundamental social motives are perceived to matter most in individuals’ own lives, only one previous study on fundamental social motives has examined perceptions of other individuals’ motives. This study focused on people’s accuracy in perceiving their friends’ motives (Huelsnitz et al., 2020). Results indicated that people were generally accurate in their perceptions of friends’ motives (although the overall level of accuracy was lower than for perceptions of friends’ traits), and greater accuracy was associated with higher friendship quality. These results are consistent with the contention that, just as it can be pragmatically useful to know someone’s behavioral traits, it is also useful to know their underlying motives. But it remains unknown which of these fundamental social motives perceivers might most want to know about.

Are there reasons to expect that perceivers might value information about some specific motives more than others? Yes, but the question of *which* motives will be prioritized is not so clear. For example, people might especially value information about motives that they believe to be most relevant to highly valued aspirations (e.g., status and affiliation motives; Krems et al., 2017), or that they subjectively judge to be important in their own lives (e.g., kin care and mate retention motives; Ko et al., 2020), or that simply seem most immediately relevant to basic biological imperatives of survival and reproduction (e.g., self-protection, disease-avoidance, and mate acquisition motives). Another possibility is that people might especially value information that is most transparently relevant to commonly experienced situations—which, based on results reported by Morse et al. (2015) implies that information about mate seeking, affiliation, and kin care motives might be most highly prioritized. Yet another perspective draws upon the body of research (e.g., Abele et al., 2021; Goodwin et al., 2014) summarized in the paragraph that opens this article: Because people value information about others’ interpersonal intentions and capacities to carry out those intentions, they might prioritize information about motives that seem relevant to “getting along” and “getting ahead” (e.g., affiliation and status motives)—and perhaps might most especially prioritize information about motives that seem especially diagnostic of moral character (e.g., kin care motive).

This question is further complicated by the tendency for perceivers to be pragmatists—with the consequence that they may be attuned to different kinds of information depending on their own goals and on situation-specific affordances (Kenrick et al., 2010; McArthur & Baron, 1983; Neel & Lassetter, 2019; Pirlott & Cook, 2018; Zebrowitz & Montepare, 2006). People differ in the extent to which different fundamental motives are chronically active (Neel et al., 2016), and this may affect the extent to which they desire information about specific motives in others. In addition, because different motives are likely to be evoked—and thus to guide behavior—in different situations (Morse et al., 2015), information about specific motives might be more highly prized in specific situations within which they are more likely to be evoked. For instance, people might especially value information about mating motives in a dating context, status-seeking motives in a workplace context, and self-protection motives in a context that connotes potential danger.

Given the many different hypotheses that can be presented as plausible, and the difficulty of adjudicating their plausibility on strictly a priori grounds, we prefer to avoid framing this research as a hypothesis-testing project. (Another reason we avoid this framing: none of the studies reported below were preregistered.) Instead, we present the results of these studies simply as evidence bearing on the central research question (What motives do people prioritize obtaining information about?) and on the more specific set of goals identified subsequently.

## Overview of Empirical Studies

We conducted five studies that were designed to accomplish four complementary goals. One goal was to assess the extent to which, when encountering others, perceivers generally prioritize obtaining information about each of the seven fundamental social motives identified earlier. A second goal was to explore the extent to which these priorities might differ depending on perceivers’ own motives, traits, and characteristics. A third goal was to explore the extent to which these priorities might vary across different social contexts. Finally, if indeed perceivers generally prioritize information about some motives more than others, it raises the question why. As one means of addressing that question, a fourth goal was to identify variables that are diagnostic of the motives that people are most interested in obtaining information about.

Studies 1 to 4 employed a common set of methods that focused primarily on the first three goals. Study 5 was designed to focus more directly on the fourth goal.^2^

## Studies 1 to 4

Studies 1 to 4 were designed to assess the extent to which, when anticipating an encounter with an unfamiliar person, participants prioritized obtaining information about each of the seven fundamental social motives identified earlier. Each study included multiple experimental conditions within which participants imagined meeting a previously unknown person and completed measures assessing their interest in obtaining information about that person’s motives. (Participants also completed measures assessing their own personality traits and motives and—in a preliminary attempt to address the fourth goal specified above—a measure assessing the perceived stability of each motive.)

One condition was included in all four studies. In this condition, participants were asked simply to “Imagine that you’re about to meet a person.” They were provided no additional details—thus imposing no constraints on the characteristics that participants might imagine that person having or on the circumstances that participants might imagine encountering them.

Additional experimental conditions provided additional details. These conditions differed across the four studies. Study 1 included two conditions in which the person’s gender was identified (participants were asked to imagine meeting either a man or a woman). Study 2 included two conditions in which participants imagined meeting the person in specific kinds of dating contexts. Study 3 included three conditions in which participants imagined meeting the person in specific workplace contexts. Study 4 included four conditions in which participants imagined a context—encountering a stranger in a dark alley—that connotes potential danger. These additional conditions in Studies 2, 3, and 4 were designed to have specific relevance to specific motives (mating motives, status motives, and self-protection motives, respectively). Therefore—although these scenarios represent just a tiny sample of the wider universe of social situations—these methods provided some opportunity to assess the extent to which perceivers’ priorities were consistent, or not, across different contexts.

All other procedures and measures were identical across these studies. Methods and results are presented as if from a single study.

## Method

### Participants

Between May and July 2017, a total of 1,502 English-speaking North American participants were recruited from Amazon’s Mechanical Turk in exchange for monetary compensation (*N*s = 300, 302, 401, and 499 for Studies 1 to 4, respectively; see Table 1 for demographic details). In all studies, the sample size exceeded the size at which effect size estimates typically stabilize (Schönbrodt & Perugini, 2013). Power analysis shows, with α set at .01 (see below), a sample size of 100 per condition would have sufficient power to detect a medium effect size with .80 power. The studies were conducted online via Qualtrics.

**Table 1.** Demographic Characteristics of Participants in Studies 1 to 4.

Total *N*	1,502
Mean Age (*SD*)	36.50 (12.19)
Sex	906 Female 596 Male
Parental status	695 Parents 807 Nonparents
Relationship status	649 Married 345 In a committed relationship 74 Dating one person 14 Dating several people 400 Single 20 Other
Ethnic background	1187 Caucasian (White)
	105 Hispanic/Latino
	121 African
	76 East Asian (e.g., Chinese, Japanese, or Korean)
	47 Southeast Asian (e.g., Indian)
	4 Middle Eastern (e.g., Iranian)
	18 Indigenous (e.g., Metis, First Nations, or Inuit)
	17 Other

*Note.* Participants could select all applicable ethnic backgrounds.

### Person Perception Scenarios

Participants were prompted to imagine a potential encounter with a person about whom they had no prior dispositional information. Each study included different experimental conditions that described different contexts within which participants were asked to imagine encountering this target person (see Table 2). Within each study, participants were randomly assigned to one experimental condition.

**Table 2.** Twelve Scenarios Used Across Different Experimental Conditions in Studies 1 to 4.

Experimental condition	Wording of the scenario
*No Social Context* (Studies 1–4; *n* = 408)	Imagine that you’re about to meet a person.
*Man* (Study 1; *n* = 99)	Imagine that you’re about to meet a man.
*Woman* (Study 1; *n* = 99)	Imagine that you’re about to meet a woman.
*Casual Sexual Partner* (Study 2; *n* = 98)	Imagine that you’re single and are looking for a casual sexual encounter. You’ve just met someone who you find incredibly attractive. After flirting with this person for a while, it seems that the two of you have sexual chemistry, and you will have the opportunity to hook up with this person for a one-night stand if you want to.
*Potential Life Partner* (Study 2; *n* = 103)	Imagine that you’re single and are looking for a long-term life partner. You’ve just met someone who might be a good match for you. After talking to this person for a while, it seems that the two of you have relationship potential, and you will have the opportunity to pursue a long-term romantic relationship if you want to.
*Workplace—Boss* (Study 3; *n* = 96)	Imagine that you’re about to start a new job, but you haven’t yet met your new boss. Because you know you’ll be spending a lot of time working under this person’s direction, you’re probably curious to learn more about what he or she is like.
*Workplace—Assistant* (Study 3; *n* = 100)	Imagine that you’re about to start a new job, but you haven’t yet met your new assistant. Because you know you’ll be spending a lot of time directing this person’s work, you’re probably curious to learn more about what he or she is like.
*Workplace—Coworker* (Study 3; *n* = 102)	Imagine that you’re about to start a new job, but you haven’t yet met your new co-worker. Because you know you’ll be spending a lot of time working alongside this person, you’re probably curious to learn more about what he or she is like.
*Dark Alley—Young Man* (Study 4; *n* = 96)	Imagine that you’re visiting a big city far from home. Late one night, as you’re walking back to your hotel, you accidentally take a wrong turn into a dark alley. At first, you’re all alone in the alley, but then you notice a young man emerge from the shadows, approaching you.
*Dark Alley—Young Woman* (Study 4; *n* = 100)	Imagine that you’re visiting a big city far from home. Late one night, as you’re walking back to your hotel, you accidentally take a wrong turn into a dark alley. At first, you’re all alone in the alley, but then you notice a young woman emerge from the shadows, approaching you.
*Dark Alley—Elderly Man* (Study 4; *n* = 99)	Imagine that you’re visiting a big city far from home. Late one night, as you’re walking back to your hotel, you accidentally take a wrong turn into a dark alley. At first, you’re all alone in the alley, but then you notice an elderly man emerge from the shadows, approaching you.
*Dark Alley—Elderly Woman* (Study 4; *n* = 102)	Imagine that you’re visiting a big city far from home. Late one night, as you’re walking back to your hotel, you accidentally take a wrong turn into a dark alley. At first, you’re all alone in the alley, but then you notice an elderly woman emerge from the shadows, approaching you.

One experimental condition (*n* = 408) was included in all four studies: Participants were simply asked to “Imagine that you’re about to meet a person” and were provided no additional details.

Eleven additional conditions (*n*s ranged from 96 to 103) included some additional details—about either the person or the situation in which participants imagined meeting the person, or both. Study 1 included two conditions in which the person was described either as a *man* or as a *woman*. Study 2 included two conditions that supplied a dating context: Participants were asked to imagine meeting a person under circumstances in which they were seeking either a *casual sexual partner* or a *potential life partner*. Study 3 included three conditions that supplied a *workplace* context: Participants were asked to imagine starting a new job and meeting their new *boss, assistant*, or *coworker*. Study 4 included four conditions in which participants were asked to imagine walking alone in an unfamiliar *dark alley* and encountering a stranger described as a *young man, young woman, elderly man*, or *elderly woman*. See Table 2 for the exact wordings of the scenarios used in these conditions.

### Measures Assessing Prioritization of Information About Specific Social Motives

In all experimental conditions, participants were then asked to complete two measures designed to assess the extent to which they prioritized obtaining information about specific motives that the target person might have. Both measures were informed by prior research identifying seven conceptually distinct fundamental social motives—self-protection, disease avoidance, affiliation, status, mate seeking, mate retention, kin care—and, more specifically, by the 11 subscales comprising a self-report measure that assesses individual differences in these seven fundamental social motives (the “Fundamental Social Motives Inventory” [FSMI]; Neel et al., 2016).

Both measures employed 11 brief descriptions indicating information about the target person’s motivational dispositions. These descriptions began with the words “*How motivated that person is to. . .*” and then contained additional summary phrases corresponding to the motives assessed by the 11 FSMI subscales. These summary phrases (accompanied, in brackets, by the relevant FSMI subscale) were as follows: “*. . .avoid dangerous people and risky situations*” [self-protection]; “*. . .avoid infectious diseases and people who carry diseases*” [disease avoidance]; “*. . .be socially included and to be part of a group*” [affiliation (group)]; “*. . .avoid being excluded or rejected by other people*” [affiliation (exclusion concern)]; “*. . .be independent and to spend time alone*” [affiliation (independence)]; “*. . .achieve high status and positions of leadership*” [status]; “*. . .seek out new romantic or sexual partners*” [mate seeking]; “*. . .maintain a loyal and long-lasting romantic relationship*” [mate retention (general)]; “*. . .avoid being cheated on or dumped by a romantic partner*” [mate retention (breakup concern)]; “*. . .be close to their family and to attend to the needs of family members*” [kin care (family)]; and “*. . .nurture and care for their children*” [kin care (children)].

These 11 descriptions were incorporated into two separate measures, one of which employed a forced-choice methodology, and the other that employed a rating scale methodology. All participants completed both measures.

#### Forced-choice method

The forced-choice measure presented participants with every pairing of these 11 motive descriptions (55 pairings in all), and, for each pairing, participants were asked to “indicate which characteristic you would be more interested in learning about [the target person].” A prioritization score for each of the 11 motive descriptions was calculated as the total number of times it was selected across all forced-choice pairings with the 10 other motive descriptions. These scores had a possible range of 0 to 10.

#### Rating scale method

The rating scale measure presented participants with each of the 11 motive descriptions, and participants were asked to “indicate how interested you would be in learning each of the following things about [the target person].” Ratings were made on a 7-point scale (1 = *Very uninterested*, 7 = *Very interested*).

### Other Measures

#### Perceived stability of motives

Participants were presented again with the 11 motive descriptions and, for each, were asked to rate “your belief about how stable, enduring, and resistant-to-change it is.” Ratings were made on a 7-point scale (1 = *Not stable at all*, 7 = *Extremely stable*).

#### Participants’ own traits and motives

In addition to several questions assessing demographic information (age, sex, parental status, etc.), participants also completed questionnaires assessing individual differences in their own personality traits and motives. Individual differences in personality traits were assessed with the *Big Five Inventory* (BFI-44; John & Srivastava, 1999), which consists of 44 trait-relevant self-descriptive statements to which participants respond by indicating their agreement on a 5-point rating scale (1 = *Disagree strongly*, 5 = *Agree strongly*). Scores for five personality trait dimensions (agreeableness, conscientiousness, extraversion, neuroticism, openness) were computed according to procedures described in John and Srivastava (1999; Cronbach’s α ranged from .81 to .88). Individual differences in participants’ motives were assessed with the FSMI (Neel et al., 2016), which consists of 65 motivation-relevant self-descriptive statements to which participants respond by indicating their agreement on a 7-point rating scale (1 = *Strongly disagree*, 7 = *Strongly agree*). Scores for the 11 subscales (identified above) were computed according to procedures described in Neel et al. (2016).^3^ (Cronbach’s α’s for the 11 subscales ranged from .77 to .94.)

## Results

### Preliminary Analytic Considerations

#### Analytic focus on seven key motive descriptions

The primary measures of interest—the forced-choice and rating scale measures assessing the extent to which participants prioritized information about specific social motives—each produced prioritization scores for 11 different motive descriptions. Of greater interest than the 11 specific descriptions are the seven conceptually distinct fundamental social motives to which one or more of these descriptions correspond.^4^ Therefore, for the sake of both conceptual parsimony and expository efficiency, we report results on only one motive description for each of the seven fundamental social motives. For the three fundamental social motives for which there was more than one description, we report results pertaining to the one description that, based on both face validity and prior empirical results (e.g., Neel et al., 2016), appears to offer the broadest representation of the underlying motivational construct. Therefore, in the results reported subsequently, we report results on the motive prioritization scores for the following seven motive descriptions: *self-protection; disease avoidance; affiliation (group); status; mate seeking; mate retention (general); kin care (family)*.^5^ (See Supplemental Materials for additional results pertaining to all 11 motive descriptions.)

#### Relations between the two measures of information prioritization

Responses on the two primary measures of motive prioritization—the forced-choice and rating scale measures—were substantially positively correlated: Across the seven focal motive descriptions, *r*s (between a prioritization score obtained from the forced-choice measure and the corresponding rating on the rating scale measure) ranged from .42 [.37, .48]^6^ (self-protection) to .62 [.58, .66] (status), all *p*’s <.001. Consequently, there is considerable similarity in the results obtained on both measures. For the primary analyses reported subsequently, we report results obtained from both measures. For some additional analyses that address follow-up questions, we fully report only the results on the forced-choice measure while more briefly reporting any notable differences from results on the rating scale measure. (Corresponding results on the rating scale measure are reported fully in Supplemental Materials.)

#### Decision rules for statistical inference

Due to the non-hypothesis-driven nature of this investigation and the large number of statistical tests that were conducted across the forced-choice and rating scale measures, results are identified as statistically significant only if *p* < .01, and inferences are drawn only if statistically significant (*p* < .01) results emerged consistently across both the forced-choice and rating scale measures. In addition, for correlational results, inferences are drawn only for correlations of *r* =.2 or greater.

### What Motive(s) Did Participants Most Highly Prioritize Obtaining Information About?

Tables 3 and 4 present mean motive prioritization scores (along with standard deviations and bootstrapped 99% confidence intervals [9,999 replications]) obtained from the forced-choice and rating scale measures. Both tables present two sets of means. One set of means was calculated within the experimental condition that prompted participants to simply “Imagine that you’re about to meet a person” (*No social context; N* = 408)—which imposed no constraints on the person that participants might imagine, nor on the circumstances within which they might imagine meeting that person. Thus, across participants, these means might tacitly represent a variety of person perception contexts. The other set of means was calculated across the 11 additional conditions that included some additional contextual details (*N* = 1,094). These means more explicitly represent a variety of person perception contexts. (Tables list the motive descriptions according to a rank ordering of means obtained from the forced-choice measure within the “No social context” condition.)

**Table 3.** Results From the Forced-Choice Measure Assessing the Prioritization of Information About Specific Motives.

	No social context (“... a person”)			Some additional context (11 different conditions)		
Motive	*M*	*SD*	99% CI	*M*	*SD*	99% CI
Kin Care (Family)	6.92^a^	2.40	[6.62, 7.22]	6.94^a^	2.33	[6.76, 7.12]
Mate Retention (General)	6.59^a^	2.27	[6.30, 6.87]	5.85^b^	2.46	[5.66, 6.04]
Affiliation (Group)	5.08^b^	2.10	[4.81, 5.35]	5.15^c^	2.33	[4.97, 5.33]
Self-protection	4.87^bc^	2.14	[4.60, 5.15]	5.82^b^	2.38	[5.64, 6.00]
Status	4.50^bcd^	2.74	[4.14, 4.84]	4.63^d^	2.80	[4.41, 4.85]
Disease Avoidance	4.14^de^	2.62	[3.81, 4.47]	4.65^d^	2.84	[4.43, 4.87]
Mate Seeking	4.04^e^	2.70	[3.69, 4.38]	3.26^e^	2.77	[3.05, 3.47]

*Note.* Within-column means that do not share a common superscript are significantly different from one another (*p* < .01). CI = confidence interval.

**Table 4.** Results From the Rating Scale Measure Assessing the Prioritization of Information About Specific Motives.

	No social context (“... a person”)			Some additional context (11 different conditions)		
Motive	*M*	*SD*	99% CI	*M*	*SD*	99% CI
Kin Care (Family)	5.69^a^	1.31	[5.53, 5.85]	5.43^a^	1.63	[5.30, 5.56]
Mate Retention (General)	5.53^a^	1.47	[5.34, 5.71]	4.89^bc^	1.87	[4.75.04]
Affiliation (Group)	4.61^bc^	1.51	[4.42, 4.80]	4.70^c^	1.57	[4.58, 4.82]
Self-protection	4.76^b^	1.58	[4.56, 4.96]	5.15^b^	1.57	[5.03, 5.27]
Status	4.53^bc^	1.77	[4.30, 4.75]	4.53^c^	1.83	[4.38, 4.67]
Disease Avoidance	4.20^c^	1.80	[3.98, 4.43]	4.47^c^	1.86	[4.33, 4.62]
Mate Seeking	4.55^bc^	1.77	[4.31, 4.77]	4.01^d^	2.02	[3.86, 4.17]

*Note.* Within-column means that do not share a common superscript are significantly different from one another (*p* < .01). CI = confidence interval.

Which specific motive(s) were most highly prioritized? In the absence of any specified social context, results on both the forced-choice and rating scale measures show that means for the *kin care (family)* and *mate retention (general)* motives were significantly higher than any of the other motives (*p*’s < .01) and were not significantly different from each other. Within the set of conditions for which some additional context was presented, both measures show that the mean for *kin care (family)* was uniquely significantly higher than any of the other motives. Although not as high as mean for *kin care (family)*, means for *self-protection* and *mate retention* motives were also relatively high in the latter set of conditions, compared with most other motives.

### Variability Across Perceivers: Effects of Individual Differences on Motive Prioritization

The preceding results indicate that, in general, participants prioritized obtaining information about some motives (e.g., kin care) more than others. These priorities might plausibly vary across participants, depending on participants’ demographic characteristics, their personality traits (measured by the BFI-44), or their own corresponding motivational tendencies (measured by the FSMI). We conducted additional analyses on motive prioritization scores to test whether—when aggregated across all experimental conditions—any of these motive prioritization scores were significantly (*p* < .01), substantially (*r* ≥ .2), and consistently (across both measures) predicted by these individual difference variables.

There was limited evidence that motive prioritization scores were influenced by demographic variables. None of the correlations with participant age, sex, nor parental status met the inferential thresholds identified above. (Age correlated negatively with the *mate seeking* prioritization score obtained from the forced-choice measure, *r* = −.20 [−.26, −.13], but the analogous correlation for the rating scale measure failed to meet inferential thresholds.) Relationship status was the only demographic variable that produced effects meeting these inferential standards. Because relationship status was measured as a multicategory variable, we ran treatment-coded regressions comparing each of five relationship categories (married; in a committed relationship; dating one person; dating several people; other) against participants who identified as being single. Results showed that *kin care (family)* was more highly prioritized among participants who were married (βs = .29 [.13, .45] and .23 [.06, .39] on the forced-choice and rating scale measures, respectively), and *status* was more highly prioritized among participants in a committed relationship (βs = .21 [.02, .39] and .23 [.04, .42] on the forced-choice and rating scale measures, respectively).

To explore possible effects of participants’ own personality traits, we computed correlations between motive prioritization scores and participants’ scores on each of five personality trait dimensions assessed by the BFI-44 (agreeableness, conscientiousness, extraversion, neuroticism, and openness). Only one correlation met the inferential thresholds identified earlier: Participants’ own agreeableness correlated positively with prioritization of information about *kin care (family)* (*r*s = .29 [.22, .35] and .22 [.15, .28] on the forced-choice and rating scale measures, respectively).

We also computed correlations between each motive prioritization score and participants’ scores on the *corresponding* subscale of the FSMI. These correlation coefficients are presented in Table 5. With one exception (*mate retention*) there were consistent, positive, and statistically significant (*p* < .01) correlations between motive prioritization scores and participants’ scores on the corresponding FSMI subscale. Mean *r*s within each column of Table 5 are .25 and .26—indicating that, in general, participants more highly prioritized information about motives that they themselves experienced most strongly.

**Table 5.** Correlations Between Specific Motive Prioritization Scores and Participants’ Own Scores on the Corresponding Subscale of the Fundamental Social Motives.

Motive	Correlation (*r*) based on forced-choice measure of motive prioritization	Correlation (*r*) based on rating scale measure of motive prioritization
Kin Care (Family)	.43 [.38, .48]	.34 [.28, .40]
Mate Retention (General)	.15 [.07, .22]	.05 [−.02, .13]
Affiliation (Group)	.26 [.20, .32]	.31 [.25, .37]
Self-protection	.23 [.16, .29]	.28 [.21, .34]
Status	.26 [.20, .32]	.37 [.31, .42]
Disease Avoidance	.16 [.09, .22]	.22 [.16, .29]
Mate Seeking	.27 [.21, .33]	.22 [.16, .29]

*Note.* 99% confidence intervals are presented in square brackets.

### Variability Across Contexts: Effects of Experimental Condition on Motive Prioritization

**Figure 1.**
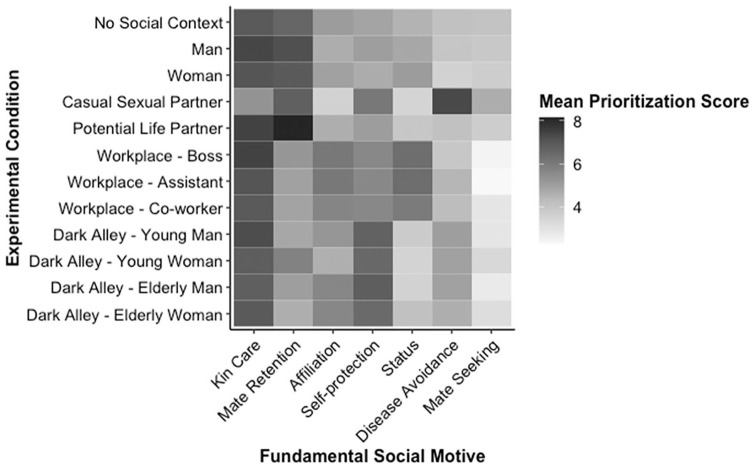
Graphical depiction of mean prioritization scores (obtained from the forced-choice measure) for each motive within each of the 12 experimental conditions. *Note.* Cell shading represents the magnitude of mean prioritization score, with darker shading indicating higher prioritization.

Figure 1 provides a graphical representation of mean prioritization scores—obtained from the forced-choice measure—for each of the seven key motives within each of the 12 different experimental conditions. An analogous figure depicting means obtained from the rating scale measure is presented in Supplemental Materials. Visual perusal of these figures suggests that mean prioritization scores varied across different appraisal contexts. To quantify this variability, we computed the standard deviation around the grand mean of the 12 condition-specific means. These standard deviations (for both the forced-choice and rating scale measures) are reported in Table 6. On the forced-choice measure, means for *kin care (family)* were most highly consistent (i.e., had the lowest variability across conditions), followed by means for *mate seeking, affiliation (group)*, and *self-protection*. On the rating scale measure, means for *affiliation (group)* and *self-protection* were most consistent, followed by *kin care (family)*.

**Table 6.** Variability of Motive Prioritization Scores Across Conditions, as Indicated by the Standard Deviation of 12 Condition-Specific Means Around the Grand Mean for Each Motive.

Motive	Forced-choice measure	Rating scale measure
Kin Care (Family)	.58	.43
Mate Retention (General)	1.17	.88
Affiliation (Group)	.73	.29
Self-protection	.74	.27
Status	1.10	.66
Disease Avoidance	.97	.60
Mate Seeking	.70	.77

The preceding results provide information about the overall extent to which there was contextual variability in motive prioritization scores, but those results do not identify s*pecific* conditions within which each motive was especially likely—or unlikely—to be prioritized.^7^

To do so, we generated linear regression models for each motive, predicting motive prioritization score as a function of the experimental condition. The 12 conditions were deviation effect-coded. The size of each β coefficient indicates the extent to which a motive’s prioritization score within a specific condition deviated from the mean of scores across all other conditions, and the sign of that coefficient indicates whether the motive was especially prioritized (positive coefficient) or deprioritized (negative coefficient) in that condition. These models yielded 30 effects that were statistically significant (*p* < .01) on *both* the forced-choice and rating scale measures. The following paragraphs report, by motive, each of these effects, which are presented in graphical format in Figure 1. For illustrative purposes, we report β coefficients (and 99% confidence intervals) from analyses on the forced-choice measure. Full results are presented in Supplemental Materials.

Information about *kin care (family)* motives was generally highly prioritized across all conditions, but there was one condition in which it was relatively less prioritized: in the context of a casual sexual partner (β = −.70 [−.95, −.46]).

Interest in obtaining information about *mate retention (general)* motives was more variable across contexts: Relative to other contexts, it was lower in the three workplace contexts (βs = −.29 [−.52, −.06], −.39 [−.61, −.16], and −.41 [−.63, −.19] in new boss, new assistant, and new coworker conditions, respectively) and in three of the four dark alley contexts (βs = −.45 [−.68, −.22], −.36 [−.58, −.13], and −.54 [−.76, −.32] in the young man, elderly man, and elderly woman conditions, respectively); it was relatively highly prioritized in both romantic contexts (βs = .34 [.12, .57] and .92 [.70, 1.14] in the casual sexual partner and potential life partner conditions, respectively), when the target person was identified simply as a man (β = .53 [.31, .76]) or woman (β = .41 [.18, .63]), and in the “No Social Context” condition (β = .28 [.16, .41]).

Information about *affiliation (group)* motives was relatively deprioritized in the contexts of a casual sexual partner (β = −.69 [−.93, −.45]) and meeting a young woman in a dark alley (β = −.25 [−.49, −.01]) but was more highly prioritized in the context of meeting a new assistant (β = .40 [.16, .64]).

There were no conditions in which information about *self-protection* was especially highly prioritized, and it was relatively deprioritized only in the “No Social Context” condition (β = −.37 [−.50, −.24]).

Information about *status* motives was relatively deprioritized in the context of a casual sexual partner (β = −.42 [−.65, −.18]) and in three of the four dark alley contexts (βs = −.30 [−.54, −.07], −.39 [−.63, −.16], and −.38 [−.62, −.15] in the young man, young woman, and elderly man conditions, respectively); it was relatively highly prioritized in all three workplace contexts (βs = .62 [.38, .86], .63 [.40, .86], and .49 [.26, .72] in new boss, new assistant, and new coworker conditions, respectively).

Information about *disease avoidance* motives was relatively deprioritized in the new boss condition (β = −.25 [−.49, −.01]) and in the “No Social Context” condition (β = −.17 [−.30, −.04]); it was relatively highly prioritized in the context of a casual sexual partner (β = .98 [.74, 1.22]).

Information about *mate seeking* motives was relatively deprioritized in two of the workplace contexts (βs = −.30 [−.54, −.05] and −.36 [−.60, −.12] in the new boss and new assistant conditions, respectively); it was more highly prioritized in the context of a casual sexual partner (β *=* .48 [.24, .72]) and in the “No Social Context” condition (β *=* .26 [.12, .39]).

Collectively, these results (which can be more readily interpreted with the graphical assistance of Figure 1) document specific ways in which social context did, or did not, affect the motives that participants wanted to obtain information about. Notably, in the absence of explicit information about specific circumstances that might evoke specific motivational concerns, participants highly prioritized information about both kin care and mate retention motives. But, while information about mate retention motives was deprioritized within the workplace and dark alley conditions (which evoked situation-specific interests in other motives), information about kin care motives remained a high priority even within those conditions. Indeed, with only one exception (the casual sexual partner condition), contextual information did not diminish participants’ strong interest in obtaining information about kin care motives.

### Relations Between Motive Prioritization Scores and Perceived Stability of Motives

The results presented thus far provide information about the motives that people are generally interested (or less interested) in obtaining information about, but they do not shed light on why. Studies 1 to 4 included one measure that might help to address that question: the measure assessing perceived stability of motives. For pragmatic reasons, people might plausibly prioritize information about dispositions that are believed to be more stable across time and circumstances. If so, then mean ratings of stability would be expected to show a pattern similar to the mean motive prioritization scores presented in Tables 3 and 4.

The mean stability ratings (with bootstrapped 99% CIs) for each motive are presented in Table 7. The relative magnitudes of these means do bear some similarity to the mean prioritization scores. This similarity is illustrated by correlational analyses that treat motive as the unit of analysis: There are positive correlations between the means presented in Table 7 and the four sets of means presented in Tables 3 and 4; *r*s (*n* = 7) ranged from .75 [−.31, .98] to .97 [.66, 1.00]. In addition, treating individual participants as the unit of analysis, we computed correlations between participants’ stability ratings for each motive and the corresponding motive prioritization scores. We computed these seven correlations separately for the four different motive prioritization scores identified in Tables 3 and 4 (i.e., separate prioritization scores obtained from the forced-choice and rating scale measures, and separate prioritization scores for subsets of participants who were in either the “No Social Context” condition or in one of the 11 other conditions). All 28 correlations were positive (*r*s ranged from .09 [−.04, .21] to .31 [.24, .38]), and 21 of these *r*’s were statistically significant (*p* < .01). (For only one motive—*kin care (family)*—were all four *r*s statistically significant and <.2 in magnitude.) Although these results must be interpreted with caution, both sets of correlations suggest that the most highly prioritized motives (e.g., kin care) were also perceived to be most stable and enduring.

**Table 7.** Results From a Measure Assessing the Extent to Which Participants Perceived Each Motive to be “Stable, Enduring, and Resistant-To-Change.”

	Perceived stability		
Motive	*M*	*SD*	99% CI
Kin Care (Family)	5.60^a^	1.25	[5.52, 5.69]
Mate Retention (General)	5.27^b^	1.44	[5.17, 5.36]
Affiliation (Group)	4.77^cd^	1.34	[4.68, 4.86]
Self-protection	4.94^c^	1.41	[4.84, 5.03]
Status	4.76^cd^	1.39	[4.67, 4.85]
Disease Avoidance	4.68^d^	1.52	[4.58, 4.78]
Mate Seeking	3.83^e^	1.52	[3.73, 3.93]

*Note.* Within-column means that do not share a common superscript are significantly different from one another (*p* < .01). CI = confidence interval.

## Discussion

Studies 1 to 4 were designed to assess the extent to which perceivers prioritize obtaining information about each of seven fundamental social motives and to explore the extent to which these priorities vary across perceivers and across contexts. Results did show some evidence of variation across perceivers (e.g., people prioritized information about motives that they themselves experienced strongly). Results also showed ample evidence of variation across contexts. For instance, workplace contexts evoked increased interest in others’ status motives; the context of a casual sexual encounter evoked increased interest in others’ disease-avoidance motives; and while participants expressed high interest in someone’s mate retention motives when the only information available was that the person was a “person” or “man” or a “woman” (and in dating contexts too), their interest in mate retention motives was substantially decreased under circumstances that evoked situation-specific interests in other motives instead. Thus, just as specific circumstances activate specific motives in perceivers (Cook et al., 2021; Morse et al., 2015; Schaller et al., 2017), specific circumstances also lead perceivers to express specific interests in obtaining information about specific motives in others. It is with these findings in mind that one additional finding is perhaps especially striking: Participants expressed especially high interest in obtaining information about others’ kin care motives and did so across an especially wide range of contexts.

Why is it that people seem to generally prioritize information about some motives more than others? It is not yet clear, although one clue is provided by additional results showing that kin care motives (and, to a lesser extent, mate retention motives) were perceived to be highly stable and resistant to change. It is also possible that, when expressing interest in specific motives, people may not be interested in those underlying motives, per se, but may instead be using that information to indirectly inform them about other things that they want to know about. For example, the generally high interest in information about kin care motives might reflect the perception that kin care motives are (compared with other motives) especially diagnostic of traits that connote behavioral propensities for “getting along” and “getting ahead” (Abele et al., 2021), or the subset of those traits that connote moral character (Goodwin et al., 2014). Study 5 was designed to provide evidence bearing on this possibility.

## Study 5

Participants in Study 5 completed a motive prioritization measure (the rating scale measure employed in Studies 1–4) and also completed a new measure assessing participants’ beliefs about the extent to which information about specific motives was diagnostic of specific behavioral traits. Treating traits as the unit of analysis, we examined correlations between mean responses on the latter measure and the mean motive prioritization scores obtained from Studies 1 to 4. We also conducted additional analyses that treated participants as the unit of analysis, assessing the extent to which individual differences in motive prioritization scores correlated with individual differences in perceived diagnosticity of traits.^8^

## Method

### Participants

Participants were 174 English-speaking North Americans recruited in February 2020 via the same procedures used in Studies 1 to 4 (see Supplemental Materials for demographic information).

### Measures

#### Trait diagnosticity measure

Participants were presented with seven scenarios that had the following structure: “Imagine that the only thing that you know about a person is: The person has a very high motivation to [brief description of motive].” The brief descriptions of motives were identical to seven descriptions that were used in Studies 1 to 4: “*. . .avoid dangerous people and risky situations*” [self-protection]; “*. . .avoid infectious diseases and people who carry diseases*” [disease avoidance]; “*. . .be socially included and to be part of a group*” [affiliation (group)]; “. . .*achieve high status and positions of leadership*” [status]; “*. . .seek out new romantic or sexual partners*” [mate seeking] “*. . .maintain a loyal and long-lasting romantic relationship*” [mate retention (general)]; and “*. . .be close to their family and to attend to the needs of family members*” [kin care (family)]. Following each scenario, participants were instructed, “Now, please rate the extent to which that specific piece of information is actually informative about whether the person is (or isn’t) . . .” This prompt was followed by rating scales for the following personality trait descriptions: *extroverted and enthusiastic; dependable and self-disciplined; complex and open to new experiences; anxious and easily upset; warm and sympathetic; capable and competent*; and *honest and trustworthy*.^9^ (Ratings were made on 7-point scales, with endpoints labeled “not at all informative” and “very informative.”)

#### Motive prioritization measure

Participants completed an abbreviated version of the rating scale motive prioritization measure used in Studies 1 to 4, specific to the condition in which no contextual information was provided (i.e., the instructions simply stated “Imagine that you’re about to meet a person. . .”). This version included items corresponding only to the seven focal motives identified earlier.

## Results and Discussion

Table 8 shows mean trait diagnosticity ratings indicating the extent to which people generally perceive each of seven motives to be informative about each of seven traits. Treating the seven motives as the unit of analysis, we computed correlations (*N* = 7) between each of seven sets of trait diagnosticity means (the means presented in the columns of Table 8) and each of the four sets of mean motive prioritization scores obtained in Studies 1 to 4 (presented in Tables 3 and 4). The results show that mean motive prioritization scores were consistently positively correlated with mean diagnosticity scores for four of the seven traits: *dependable and self-disciplined* (*r*’s ranged from .58 [−.55, .96] to .70 [−.40, .97]); *capable and competent* (*r*’s ranged from .53 [−.60, .95] to .57 [−.57, .96]); *warm and sympathetic* (*r*s ranged from .58 [−.56, .96] to .93 [.35, .99]); *honest and trustworthy* (*r*’s ranged from .66 [−.46, .97] to .95 [.50, 1.00]). Although these results certainly cannot compel confident inferences, they are consistent with the following interpretation: The motives that people generally prioritize obtaining information about (e.g., kin care) are also generally perceived to be diagnostic of behavioral propensities for “getting along” and “getting ahead”—and perhaps especially diagnostic of the subset of those traits that connote moral character (e.g., trustworthy).

**Table 8.** Mean Trait Diagnosticity Ratings (and *SD*s) Indicating the Extent to Which Information About a Specific Motive is Perceived to be Diagnostic of a Specific Trait.

	Trait						
Motive	Extroverted and enthusiastic	Dependable and self-disciplined	Complex and open to new experiences	Anxious and easily upset	Warm and sympathetic	Capable and competent	Honest and trustworthy
Kin Care (Family)	4.76 (1.80)	5.44 (1.33)	4.37 (1.85)	3.87 (1.80)	5.66 (1.25)	5.39 (1.42)	5.41 (1.32)
Mate Retention (General)	4.68 (1.72)	5.26 (1.57)	4.56 (1.73)	3.83 (1.90)	5.60 (1.22)	5.02 (1.59)	5.58 (1.45)
Affiliation (Group)	5.55 (1.30)	4.73 (1.86)	5.31 (1.37)	4.20 (1.86)	5.24 (1.50)	4.63 (1.84)	4.83 (1.66)
Self-Protection	4.52 (1.83)	4.81 (1.77)	4.48 (1.87)	4.95 (1.73)	4.58 (1.79)	4.70 (1.84)	4.59 (1.82)
Status	5.30 (1.43)	5.44 (1.32)	5.08 (1.57)	3.76 (1.85)	4.70 (1.76)	5.49 (1.33)	4.79 (1.65)
Disease Avoidance	4.13 (1.87)	4.76 (1.68)	4.17 (1.90)	5.06 (1.57)	4.36 (1.85)	4.47 (1.86)	4.56 (1.77)
Mate Seeking	5.44 (1.41)	4.21 (1.84)	5.31 (1.53)	3.72 (1.88)	4.65 (1.74)	4.36 (1.88)	4.35 (1.95)

Because participants completed a rating scale motive prioritization measure,^10^ we also conducted additional exploratory analyses that treated individual participants as units of analysis. For each motive, we computed correlations (*N* = 174) between participants’ motive prioritization rating and their corresponding trait diagnosticity ratings (indicating the extent to which that motive was perceived to be diagnostic of each of the seven traits). Tables reporting these results can be found in Supplemental Materials. Most (48 of 49) *r*s were positive. This pattern could indicate that individual differences in motive prioritization scores reflect individual differences in the extent to which motives are perceived to be diagnostic of *any* behavioral trait, but it could also be a statistical artifact resulting from individual differences in rating scale usage. Within this set of generally positive correlations, one additional finding is worth noting in light of the results (reported earlier) showing that (a) participants highly prioritized information about kin care and mate retention motives, and also (b) perceived these motives to be highly diagnostic of warmth and trustworthiness: Individual participants’ prioritization ratings for both *kin care (family)* and *mate retention (general)* were only weakly correlated with their trait diagnosticity ratings for *warm and sympathetic* and *honest and trustworthy*. These relatively modest correlations, along with the relatively low *SD*’s associated with these particular diagnosticity ratings (see Table 8), suggest the following interpretation: Because people did not substantially vary in their (generally strong) beliefs that kin care and mate retention motives are diagnostic of warmth and trustworthiness, individual differences in these beliefs did not readily predict individual-level variability around the general tendency to highly prioritize information about these particular motives.

## General Discussion

What motives might perceivers most highly prioritize obtaining information about, and to what extent do these priorities vary across different perceivers and different social contexts? To address those questions, we assessed the extent to which perceivers prioritized obtaining information about each of seven fundamental social motives: self-protection, disease avoidance, affiliation, status, mate seeking, mate retention, and kin care. Data were obtained from a large sample of people who varied in their own characteristics, traits, and motives, and these data were obtained within 12 experimental conditions that varied in terms of specific contextual circumstances. One notable result was the finding that most people, in most contexts, highly prioritized obtaining information about kin care motives.

The results did show some evidence of individual differences; but these effects were few, modest in size, and did not substantially moderate the extent to which information about specific motives was prioritized. In addition—and consistent with pragmatic perspectives on person perception (Fiske, 1992; McArthur & Baron, 1983; Neel & Lassetter, 2019; Pirlott & Cook, 2018)—there was variability across contexts. For instance, in the absence of information about specific situations that might activate specific person-perception goals, participants highly prioritized obtaining information not only about a person’s kin care motives but their mate retention motives too; however, interest in mate retention motives was reduced under several different circumstances that evoked context-specific interests in other motives instead. Another example: Interest in obtaining information about another person’s disease-avoidance motives was generally low, but it was highly prioritized in the specific condition in which participants were asked to consider that person as a potential sexual partner. That same context—a casual sexual encounter—was also notable for being the only condition in which interest in kin care motives was deprioritized. Across all other circumstances examined here, participants expressed a strong interest in obtaining information about kin care motives.

Why might this be? Several additional results offer clues. Results from Studies 1 to 4 showed that, compared with other fundamental motives, a person’s kin care motives were perceived to be especially stable. This finding is perhaps especially intriguing when considered alongside prior evidence suggesting that moral character is also perceived to persist (Goodwin et al., 2014; White et al., 2020). Additional results from Study 5 revealed that both kin care and mate retention motives were perceived to be highly diagnostic of traits connoting behavioral inclinations toward competence, dependability, warmth, and trustworthiness. These results suggest that, in most contexts, the goals that govern perceivers’ interests in someone’s underlying motives are the same pragmatic goals that underlie perceivers’ interests in someone’s behavioral traits: They want to know about that person’s interpersonal intentions and capacity to carry out those intentions (Abele et al., 2021; Fiske et al., 2007)—and, perhaps above all, they want to know that person’s moral character (Goodwin et al., 2014). The results also suggest that people perceive kin care motives (and mate retention motives too) to be especially useful as a means of inferring moral character—which further suggests that laypeople tacitly recognize the fact that, in everyday life, “familial” motivational systems have behavioral implications that extend far beyond the specific pair-bonding and parental caregiving contexts within which they originally evolved (Ko et al., 2020; Schaller et al., 2017; Schaller, 2018).

There is an intriguing thematic convergence between these results and the findings reported by Ko et al. (2020)—which showed that people place a particularly high value on their own kin care and mate retention motives. Might there be a causal relation between the high importance that people ascribe to their *own* “familial” motives and their keen interest in obtaining information about *others’* such motives? Perhaps, and, if so, the causal arrow might plausibly point in either direction. Keen interest in others’ kin care motives might be a consequence of the high importance people place on their own analogous motives, or, alternatively, the high importance people place on their own kin care motives might be a consequence of their keen interest in others’ such motives. Future research will be required to ascertain whether any such causal relation exists.

### Limitations and Future Directions

Future research will also be required to address limitations associated with the specific methodological choices we made for the purposes of collecting these data. For instance, we employed recruitment methods designed to ensure diversity along several demographic dimensions that are relevant to fundamental motives and person perception (e.g., relationship status and parental status), but the resulting samples were not diverse along other dimensions. All participants were computer-literate Americans, for example. Generalizability across cultures remains unknown.

Another limitation arises from the fact that there are many different circumstances within which perceivers might seek information about others’ motives, and our methods represented only a subset of them. These specific contexts (dating contexts, workplace contexts, contexts in which people might feel vulnerable to physical harm) were chosen because they seemed likely to evoke different kinds of motivational concerns—thus providing one means of exploring cross-situational variability in the extent to which perceivers prioritized obtaining information about each of the seven focal motives. But this approach did not accord equal representation to many other kinds of situations (e.g., cocktail parties, treaty negotiations, and hospital waiting rooms) that may have rather different situation-specific effects on the motives that people want to know about. We suggested earlier that a wider range of situations might have been tacitly represented across the 408 participants in the “No Social Context” condition (within which no constraints were imposed on the kinds of situations that participants might imagine meeting someone), but we gathered no evidence to substantiate that suggestion. The upshot: Although our results show that participants typically prioritized information about some motives (e.g., kin care) more than others, it would be premature to generalize that inference beyond the specific set of social circumstances represented within our methods. It will be informative for future research to use more comprehensive samplings of social situations to more systematically articulate the effects of specific contexts on perceivers’ priorities and to allow more confident conclusions about the motives that people generally most want to know about.

A third limitation pertains to our use of hypothetical scenarios. While these kinds of methods are common in research on social cognition, responses to hypothetical scenarios cannot perfectly predict how people think, feel, or behave when presented with real people in real life (Wilson & Gilbert, 2003). Hypothetical scenarios inevitably omit countless perceptual stimuli that are present in real-world social interactions, which might potentially affect the specific kinds of information that perceivers seek to obtain about others’ motives. It may be useful for future research to use complementary methods, such as experience sampling, to address this limitation.

It will also be informative for future research to assess perceivers’ interest in obtaining information about *other* motives that were not included within the empirical studies reported here. These studies focused specifically on seven motivational systems that have been the focus of conceptual and empirical work within the fundamental social motives framework (Cook et al., 2021; Huelsnitz et al., 2020; Kenrick et al., 2010; Ko et al., 2020; Krems et al., 2017; Neel et al., 2016). These seven motivational systems cover a broad range of conceptual territory but—because the framework itself is logically constrained by the biological principles that inform it (Schaller et al., 2010, 2017)—they do not represent an exhaustive list of motives that matter. Other research on human motivation identifies many other motives too—including motives pertaining to constructs as conceptually diverse as food (Anselme & Güntürkün, 2019), power (McClelland, 1985), autonomy (Deci & Ryan, 2008), cognitive closure (Kruglanski et al., 2010), and meaning (Heine et al., 2006). To what extent might perceivers want to know how strongly other people experience these and other motives? How might perceivers’ interest in obtaining that information compare with their zeal to obtain information about the motives that were highly prioritized in the results reported here? And what exactly is it about these motives that explain whether perceivers do or do not prioritize obtaining information about them? Additional empirical research will be required to answer those questions. The current results represent just one step toward a more complete empirical understanding of the kinds of personality-relevant information—which includes motives as well as traits (Winter et al., 1998)—that people want to know about other people.

## Supplemental Material

sj-docx-1-psp-10.1177_01461672211069468 – Supplemental material for What Motives Do People Most Want to Know About When Meeting Another Person? An Investigation Into Prioritization of Information About Seven Fundamental Motives Supplemental material, sj-docx-1-psp-10.1177_01461672211069468 for What Motives Do People Most Want to Know About When Meeting Another Person? An Investigation Into Prioritization of Information About Seven Fundamental Motives by Matthew I. Billet, Hugh C. McCall and Mark Schaller in Personality and Social Psychology Bulletin

sj-docx-2-psp-10.1177_01461672211069468 – Supplemental material for What Motives Do People Most Want to Know About When Meeting Another Person? An Investigation Into Prioritization of Information About Seven Fundamental Motives Supplemental material, sj-docx-2-psp-10.1177_01461672211069468 for What Motives Do People Most Want to Know About When Meeting Another Person? An Investigation Into Prioritization of Information About Seven Fundamental Motives by Matthew I. Billet, Hugh C. McCall and Mark Schaller in Personality and Social Psychology Bulletin

## Notes

1 Affiliation, status, mate seeking, mate retention, and kin care apply to specific domains of social life. Self-protection and disease avoidance are not specific to the social realm, but—because other people are potential sources of harm or infection—these two motives also regulate social cognition and social interaction (e.g., Murray & Schaller, 2016; Neuberg et al., 2011)

2 All manipulations, measures, and exclusions are reported either within the Methods and Results sections or in Supplemental Materials. Datasets for all studies are available at https://osf.io/2e37m/?view_only=e2a241895ff94c0b98b3d1b8f7cf39ad

3 In accordance with FSMI instructions (Neel et al., 2016), items associated with the Kin Care (Children) subscale were completed by participants only if they indicated that they were parents, and items associated with the Mate Retention subscales were completed by participants only if they indicated that they were in an ongoing romantic relationship.

4 The mismatch between underlying motives and descriptions is isomorphic with the corresponding mismatch between underlying motives and FSMI subscales (Neel et al., 2016). The FSMI includes a single subscale for each of four motives (self-protection, disease avoidance, status, and mate seeking), two subscales each for mate retention and kin care motives, and three subscales for the affiliation motive.

5 This presentational choice does not affect the scoring of the motive prioritization scores, described in the “Method” section.

6 Throughout this article, values presented in square brackets are 99% confidence intervals.

7 Some readers might also be interested in a complementary question: Which specific motives had especially high—or especially low—prioritization scores within each experimental condition, relative to the prioritization of other motive information within that condition? That question is addressed by additional analyses presented in the Supplemental Materials.

8 We also conducted another study (*N* = 303) in which a different measure was used to assess diagnosticity of behavioral traits but in which participants did not complete any motive prioritization measure. The limited set of results replicated most, but not all, of the corresponding results of Study 5. A full description can be found in Supplemental Materials.

9 The first five trait descriptions were adapted from a short measure of the “big 5” personality traits (Gosling et al., 2003) and correspond to the following behavioral dispositions: extraversion, conscientiousness, openness, neuroticism, and agreeableness.

10 Results on this motive prioritization measure were consistent with results from Studies 1 to 4. For example, mean ratings were highest for *mate retention (general)* and *kin care (family)* and the lowest for *disease avoidance* and *mate seeking*.
