# Supplementary material for: What Motives Do People Most Want to Know About When Meeting Another Person? An Investigation Into Prioritization of Information About Seven Fundamental Motives
Source: Pers Soc Psychol Bull. 2022 Jan 26;49(4):495–509. doi: 10.1177/01461672211069468 (PMC9989231; doi:10.1177/01461672211069468)
Supplement: sj-docx-1-psp-10.1177_01461672211069468 – Supplemental material for What Motives Do People Most Want to Know About When Meeting Another Person? An Investigation Into Prioritization of Information About Seven Fundamental Motives [file sj-docx-1-psp-10.1177_01461672211069468.docx]

Supplemental Materials

What Motives do People Most Want to Know About When Meeting Another Person? An Investigation into Prioritization of Information about Seven Fundamental Motives

Table of Contents

[Demographics Information 3](#_Toc87782047)

[Exclusions 4](#_Toc87782048)

[What Motive(s) Did Participants Most Highly Prioritize Obtaining Information About? 5](#_Toc87782049)

[With and Without a Social Context 5](#_Toc87782050)

[Forced-Choice Measure 5](#_Toc87782051)

[Rating Scale Measure 6](#_Toc87782052)

[Motive Means & Distribution Info by Specific Social Context 7](#_Toc87782053)

[Motive Prioritization Across Social Contexts: Rating Scale Heat Map 13](#_Toc87782054)

[Variability of Motive Prioritization Across Conditions (Standard Deviations around Grand Mean) 14](#_Toc87782055)

[Specific Conditions in which each Motive was Prioritized 15](#_Toc87782056)

[Forced-Choice Measure 15](#_Toc87782057)

[Rating Scale Measure 17](#_Toc87782058)

[Alternative Analysis: Prioritized motives relative to other motives within a condition 19](#_Toc87782059)

[Forced-Choice Measure 19](#_Toc87782060)

[Study 1: Gender 19](#_Toc87782061)

[Study 2: Dating 20](#_Toc87782062)

[Study 3: Workplace 21](#_Toc87782063)

[Study 4: Dark Alley 22](#_Toc87782064)

[Rating Scale Measure 23](#_Toc87782065)

[Study 1: Gender 23](#_Toc87782066)

[Study 2: Dating 24](#_Toc87782067)

[Study 3: Workplace 25](#_Toc87782068)

[Study 4: Dark Alley 26](#_Toc87782069)

[Motive Stability 27](#_Toc87782070)

[Mean Motive Stability Ratings 27](#_Toc87782071)

[Motive Stability Correlations with Motive Prioritization 28](#_Toc87782072)

[7 Key Fundamental Social Motives 28](#_Toc87782073)

[All 11 Fundamental Social Motive Subscales 28](#_Toc87782074)

[Study 5: Trait Inference 29](#_Toc87782075)

[Mean Motive Prioritization Ratings: Rating Scale Measure 29](#_Toc87782076)

[Mean Trait Inference Ratings 30](#_Toc87782077)

[Correlations Among Motive Prioritization Scores 32](#_Toc87782078)

[Trait Inference Correlations with Motive Prioritization Scores 33](#_Toc87782079)

[Motive as Unit of Analysis 33](#_Toc87782080)

[Individual as Unit of Analysis 34](#_Toc87782081)

[Trait Inference Pilot (Unreported in Manuscript) 38](#_Toc87782082)

[Methods and Procedure 38](#_Toc87782083)

[Mean Trait Inference Ratings 39](#_Toc87782084)

[Trait Inference Correlations with Motive Prioritization 42](#_Toc87782085)

# Demographics Information

|  | **Study 1 (Gender)** | **Study 2 (Dating)** | **Study 3 (Workplace)** | **Study 4 (Dark Alley)** | **Overall** |
| --- | --- | --- | --- | --- | --- |
| **N** | **300** | **302** | **401** | **499** | **1502** |
| **Age** |  |  |  |  |  |
| Mean (SD) | 36.8 (12.8) | 34.8 (10.8) | 37.7 (12.5) | 36.4 (12.3) | 36.5 (12.2) |
| Median [Min, Max] | 33.0 [19.0, 90.0] | 32.0 [19.0, 71.0] | 34.0 [19.0, 88.0] | 33.0 [19.0, 80.0] | 33.0 [19.0, 90.0] |
| **Sex** |  |  |  |  |  |
| Female | 190 (63.3%) | 167 (55.3%) | 225 (56.1%) | 324 (64.9%) | 906 (60.3%) |
| Male | 110 (36.7%) | 135 (44.7%) | 176 (43.9%) | 175 (35.1%) | 596 (39.7%) |
| **Parenthood Status** |  |  |  |  |  |
| Non-Parent | 154 (51.3%) | 170 (56.3%) | 217 (54.1%) | 266 (53.3%) | 807 (53.7%) |
| Parent | 146 (48.7%) | 132 (43.7%) | 184 (45.9%) | 233 (46.7%) | 695 (46.3%) |
| **Relationship Status** |  |  |  |  |  |
| Married | 141 (47.0%) | 120 (39.7%) | 181 (45.1%) | 207 (41.5%) | 649 (43.2%) |
| Committed | 60 (20.0%) | 77 (25.5%) | 90 (22.4%) | 118 (23.6%) | 345 (23.0%) |
| Dating | 18 (6.0%) | 19 (6.3%) | 12 (3.0%) | 25 (5.0%) | 74 (4.9%) |
| Dating Mult. | 1 (0.3%) | 5 (1.7%) | 5 (1.2%) | 3 (0.6%) | 14 (0.9%) |
| Single | 79 (26.3%) | 77 (25.5%) | 108 (26.9%) | 136 (27.3%) | 400 (26.6%) |
| Other | 1 (0.3%) | 4 (1.3%) | 5 (1.2%) | 10 (2.0%) | 20 (1.3%) |

|  | | **Study 5** | | | **Pilot Study (Unreported)** | |
| --- | --- | --- | --- | --- | --- | --- |
| **N** | | **174** | | | **303** | |
| **Age** |  | | **Ethnicity** |  | **Age** |  |
| 18 - 24 | 14 (8.0%) | | Asian | 90 (51.7%) | Mean (SD) | 37.9 (12.3) |
| 25 - 34 | 97 (55.7%) | | White | 69 (39.7%) | Median [Min, Max] | 34.0 [19.0, 76.0] |
| 35 - 44 | 40 (23.0%) | | Other | 15 (8.6%) | **Sex** |  |
| 45 - 54 | 14 (8.0%) | | **Religion** |  | Female | 188 (62.0%) |
| 55 - 64 | 7 (4.0%) | | Christian | 75 (43.1%) | Male | 115 (38.0%) |
| 65 - 74 | 2 (1.1%) | | Hindu | 56 (32.2%) |  |  |
| **Sex** |  | | Other | 43 (24.7%) |  |  |
| Female | 70 (40.2%) | | **Education** |  |  |  |
| Male | 104 (59.8%) | | Post-Secondary Degree | 138 (79.3%) |  |  |
| **Income** |  | |  |  |  |  |
| $0 - $39k | 74 (42.5%) | |  |  |  |  |
| $40k - $99k | 82 (47.1%) | |  |  |  |  |
| $100k+ | 18 (10.3%) | |  |  |  |  |

# Exclusions

In Studies 1 – 4 there were 3 participants who reported ages below the age of consent (2, 5, and 11 years old). Participants were in the *man*, *new boss,* and *elderly woman dark alley* conditions. These participants were excluded from analyses.

In Study 5, 222 participants completed the survey. 24 participants did not pass the attention check (the check was to respond “Other” and then enter the name “Tolstoy”. Participants passed the check if they spelled Tolstoy approximately correctly). 10 participants indicated they did not wish for their data to be analyzed for one reason or another. 14 participants were excluded for having duplicate IP addresses.

# What Motive(s) Did Participants Most Highly Prioritize Obtaining Information About?

## With and Without a Social Context

### Forced-Choice Measure

| **Motive** | **No Context** | | | | | | **Context** | | | | | |
| --- | --- | --- | --- | --- | --- | --- | --- | --- | --- | --- | --- | --- |
|  |  | | *99% CI* | |  | |  | | *99% CI* | |  | |
|  | **Mean** | **Lower** | | **Upper** | | **SD** | **Mean** | **Lower** | | **Upper** | | **SD** |
| Kin Care (Fam) | 6.924 | 6.618 | | 7.221 | | 2.396 | 6.941 | 6.755 | | 7.122 | | 2.328 |
| Mate Retention (Gen) | 6.593 | 6.304 | | 6.873 | | 2.270 | 5.848 | 5.658 | | 6.042 | | 2.463 |
| Kin Care (Child) | 6.397 | 6.032 | | 6.752 | | 2.859 | 6.274 | 6.055 | | 6.495 | | 2.817 |
| Affiliation (Indep) | 5.525 | 5.240 | | 5.816 | | 2.244 | 5.240 | 5.060 | | 5.418 | | 2.257 |
| Affiliation (Group) | 5.076 | 4.806 | | 5.346 | | 2.096 | 5.154 | 4.973 | | 5.332 | | 2.332 |
| Self-Protection | 4.873 | 4.598 | | 5.150 | | 2.136 | 5.819 | 5.635 | | 6.002 | | 2.375 |
| Status | 4.495 | 4.145 | | 4.841 | | 2.744 | 4.628 | 4.412 | | 4.848 | | 2.803 |
| Disease Avoidance | 4.142 | 3.811 | | 4.473 | | 2.616 | 4.647 | 4.428 | | 4.870 | | 2.836 |
| Mate Seeking | 4.037 | 3.694 | | 4.375 | | 2.701 | 3.257 | 3.047 | | 3.469 | | 2.772 |
| Affiliation (Excl) | 3.797 | 3.527 | | 4.081 | | 2.187 | 4.175 | 4.013 | | 4.345 | | 2.133 |
| Mate Retention (Breakup) | 3.142 | 2.863 | | 3.424 | | 2.180 | 3.017 | 2.844 | | 3.191 | | 2.215 |

### Rating Scale Measure

| **Motive** | **No Context** | | | | | | **Context** | | | | | |
| --- | --- | --- | --- | --- | --- | --- | --- | --- | --- | --- | --- | --- |
|  |  | | *99% CI* | |  | |  | | *99% CI* | |  | |
|  | **Mean** | **Lower** | | **Upper** | | **SD** | **Mean** | **Lower** | | **Upper** | | **SD** |
| Kin Care (Fam) | 5.694 | 5.527 | | 5.850 | | 1.306 | 5.432 | 5.304 | | 5.559 | | 1.628 |
| Mate Retention (Gen) | 5.527 | 5.338 | | 5.706 | | 1.470 | 4.893 | 4.751 | | 5.038 | | 1.869 |
| Kin Care (Child) | 5.439 | 5.223 | | 5.654 | | 1.693 | 5.339 | 5.198 | | 5.474 | | 1.787 |
| Affiliation (Indep) | 5.353 | 5.181 | | 5.512 | | 1.311 | 5.097 | 4.981 | | 5.214 | | 1.503 |
| Self-Protection | 4.757 | 4.559 | | 4.963 | | 1.576 | 5.152 | 5.031 | | 5.275 | | 1.571 |
| Affiliation (Group) | 4.613 | 4.419 | | 4.804 | | 1.509 | 4.701 | 4.577 | | 4.820 | | 1.565 |
| Mate Seeking | 4.547 | 4.313 | | 4.771 | | 1.774 | 4.013 | 3.855 | | 4.168 | | 2.024 |
| Status | 4.531 | 4.297 | | 4.752 | | 1.765 | 4.527 | 4.384 | | 4.669 | | 1.829 |
| Affiliation (Excl) | 4.248 | 4.049 | | 4.449 | | 1.580 | 4.475 | 4.353 | | 4.599 | | 1.607 |
| Disease Avoidance | 4.203 | 3.975 | | 4.429 | | 1.798 | 4.474 | 4.331 | | 4.621 | | 1.861 |
| Mate Retention (Breakup) | 4.123 | 3.904 | | 4.346 | | 1.727 | 3.881 | 3.747 | | 4.017 | | 1.777 |

# Motive Means & Distribution Info by Specific Social Context

| **Motive** | **Condition** | **Forced-Choice Measure** | | | | **Rating Scale Measure** | | | |
| --- | --- | --- | --- | --- | --- | --- | --- | --- | --- |
|  |  | ***M*** | ***SD*** | **Skew** | **Kurt.** | ***M*** | ***SD*** | **Skew** | **Kurt** |
| Kin Care (Fam) | Man | 7.39 | 2.12 | -0.99 | 0.91 | 6.04 | 1.27 | -1.62 | 2.30 |
| Kin Care (Fam) | Woman | 7.06 | 2.33 | -0.88 | -0.09 | 5.63 | 1.63 | -1.48 | 1.65 |
| Kin Care (Fam) | No Context | 6.92 | 2.40 | -0.69 | -0.26 | 5.69 | 1.31 | -1.34 | 1.77 |
| Kin Care (Fam) | Casual Sexual Partner | 5.29 | 2.60 | 0.02 | -0.95 | 4.94 | 1.81 | -0.74 | -0.66 |
| Kin Care (Fam) | Potential Life Partner | 7.46 | 1.75 | -0.92 | 0.63 | 6.25 | 0.98 | -2.01 | 5.40 |
| Kin Care (Fam) | New Boss | 7.46 | 2.36 | -0.70 | -0.57 | 5.71 | 1.53 | -1.48 | 1.90 |
| Kin Care (Fam) | New Assistant | 7.04 | 2.04 | -0.56 | -0.23 | 5.45 | 1.49 | -1.03 | 0.56 |
| Kin Care (Fam) | New Coworker | 6.92 | 2.45 | -0.86 | 0.12 | 5.17 | 1.73 | -1.00 | 0.03 |
| Kin Care (Fam) | Young Man Dark Alley | 7.25 | 2.15 | -0.75 | 0.16 | 5.47 | 1.54 | -1.19 | 0.91 |
| Kin Care (Fam) | Young Woman Dark Alley | 6.83 | 2.49 | -0.84 | -0.04 | 5.01 | 1.88 | -0.90 | -0.38 |
| Kin Care (Fam) | Elderly Man Dark Alley | 6.75 | 2.45 | -0.63 | -0.29 | 5.02 | 1.79 | -0.74 | -0.54 |
| Kin Care (Fam) | Elderly Woman Dark Alley | 6.89 | 2.07 | -0.33 | -0.58 | 5.06 | 1.51 | -0.88 | 0.20 |
| Affiliation (Excl) | Man | 3.24 | 1.89 | 0.37 | -0.35 | 4.43 | 1.48 | -0.43 | -0.45 |
| Affiliation (Excl) | Woman | 3.36 | 1.85 | 0.37 | -0.24 | 4.23 | 1.75 | -0.23 | -0.98 |
| Affiliation (Excl) | No Context | 3.80 | 2.19 | 0.39 | -0.52 | 4.25 | 1.58 | -0.26 | -0.77 |
| Affiliation (Excl) | Casual Sexual Partner | 3.41 | 1.94 | 0.25 | -0.31 | 4.01 | 1.61 | -0.12 | -0.95 |
| Affiliation (Excl) | Potential Life Partner | 3.22 | 1.94 | 0.46 | -0.29 | 4.53 | 1.45 | -0.32 | -0.78 |
| Affiliation (Excl) | New Boss | 4.27 | 2.05 | 0.11 | -0.79 | 4.56 | 1.52 | -0.65 | 0.08 |
| Affiliation (Excl) | New Assistant | 4.65 | 1.94 | 0.00 | -0.35 | 5.03 | 1.56 | -0.81 | -0.02 |
| Affiliation (Excl) | New Coworker | 4.43 | 2.00 | 0.03 | -0.50 | 4.34 | 1.67 | -0.46 | -0.82 |
| Affiliation (Excl) | Young Man Dark Alley | 4.81 | 2.40 | 0.26 | -0.65 | 4.57 | 1.68 | -0.35 | -0.88 |
| Affiliation (Excl) | Young Woman Dark Alley | 4.32 | 2.15 | 0.21 | -0.55 | 4.39 | 1.68 | -0.39 | -0.67 |
| Affiliation (Excl) | Elderly Man Dark Alley | 5.07 | 1.97 | -0.01 | -0.06 | 4.56 | 1.57 | -0.54 | -0.50 |
| Affiliation (Excl) | Elderly Woman Dark Alley | 5.14 | 2.06 | -0.06 | -0.48 | 4.56 | 1.55 | -0.53 | -0.41 |
| Self-Protection | Man | 5.02 | 2.36 | 0.10 | -0.85 | 4.86 | 1.54 | -0.78 | 0.18 |
| Self-Protection | Woman | 4.69 | 2.22 | 0.48 | -0.22 | 4.79 | 1.66 | -0.55 | -0.51 |
| Self-Protection | No Context | 4.87 | 2.14 | 0.09 | -0.34 | 4.76 | 1.58 | -0.59 | -0.38 |
| Self-Protection | Casual Sexual Partner | 6.07 | 2.29 | -0.20 | -0.81 | 5.57 | 1.44 | -1.27 | 1.57 |
| Self-Protection | Potential Life Partner | 5.06 | 1.89 | 0.12 | -0.57 | 5.21 | 1.25 | -0.79 | 0.55 |
| Self-Protection | New Boss | 5.61 | 2.22 | 0.02 | -0.93 | 4.96 | 1.68 | -0.91 | 0.15 |
| Self-Protection | New Assistant | 5.59 | 1.85 | 0.05 | -0.10 | 5.08 | 1.45 | -0.47 | -0.33 |
| Self-Protection | New Coworker | 5.61 | 2.04 | -0.03 | -0.53 | 4.89 | 1.60 | -0.70 | -0.43 |
| Self-Protection | Young Man Dark Alley | 6.69 | 2.77 | -0.55 | -0.76 | 5.42 | 1.69 | -0.84 | -0.14 |
| Self-Protection | Young Woman Dark Alley | 6.49 | 2.26 | 0.02 | -1.03 | 5.33 | 1.58 | -0.97 | 0.29 |
| Self-Protection | Elderly Man Dark Alley | 6.78 | 2.30 | -0.35 | -0.56 | 5.42 | 1.49 | -0.67 | -0.43 |
| Self-Protection | Elderly Woman Dark Alley | 6.44 | 2.75 | -0.49 | -0.78 | 5.16 | 1.71 | -0.65 | -0.48 |
| Kin Care (Child) | Man | 6.85 | 2.66 | -0.81 | -0.21 | 5.96 | 1.51 | -1.65 | 1.99 |
| Kin Care (Child) | Woman | 6.39 | 3.14 | -0.66 | -0.82 | 5.36 | 1.90 | -0.96 | -0.33 |
| Kin Care (Child) | No Context | 6.40 | 2.86 | -0.55 | -0.65 | 5.44 | 1.69 | -1.15 | 0.49 |
| Kin Care (Child) | Casual Sexual Partner | 4.97 | 2.94 | -0.20 | -1.05 | 4.65 | 2.02 | -0.46 | -1.20 |
| Kin Care (Child) | Potential Life Partner | 6.67 | 2.83 | -0.87 | -0.44 | 5.97 | 1.59 | -1.93 | 3.09 |
| Kin Care (Child) | New Boss | 6.40 | 2.37 | -0.43 | -0.48 | 5.51 | 1.72 | -1.27 | 0.83 |
| Kin Care (Child) | New Assistant | 5.93 | 2.72 | -0.22 | -0.78 | 5.26 | 1.57 | -0.82 | -0.08 |
| Kin Care (Child) | New Coworker | 5.97 | 2.84 | -0.28 | -0.99 | 4.99 | 1.86 | -0.80 | -0.45 |
| Kin Care (Child) | Young Man Dark Alley | 6.94 | 2.69 | -0.69 | -0.50 | 5.67 | 1.52 | -1.14 | 0.66 |
| Kin Care (Child) | Young Woman Dark Alley | 6.41 | 2.96 | -0.58 | -0.71 | 5.12 | 1.94 | -0.80 | -0.68 |
| Kin Care (Child) | Elderly Man Dark Alley | 6.48 | 2.88 | -0.35 | -1.18 | 5.27 | 1.80 | -0.90 | -0.29 |
| Kin Care (Child) | Elderly Woman Dark Alley | 6.02 | 2.48 | -0.19 | -0.78 | 4.97 | 1.72 | -0.67 | -0.38 |
| Affiliation (Indep) | Man | 5.14 | 2.30 | -0.12 | -0.29 | 5.38 | 1.27 | -0.71 | 0.00 |
| Affiliation (Indep) | Woman | 6.12 | 2.12 | -0.22 | -0.32 | 5.73 | 1.25 | -1.46 | 2.72 |
| Affiliation (Indep) | No Context | 5.52 | 2.24 | -0.04 | -0.38 | 5.35 | 1.31 | -0.90 | 0.79 |
| Affiliation (Indep) | Casual Sexual Partner | 5.22 | 2.25 | -0.12 | -0.64 | 5.08 | 1.52 | -0.82 | 0.20 |
| Affiliation (Indep) | Potential Life Partner | 4.70 | 2.16 | 0.25 | -0.34 | 5.60 | 1.08 | -0.61 | -0.06 |
| Affiliation (Indep) | New Boss | 4.90 | 1.97 | -0.05 | -0.32 | 4.78 | 1.52 | -0.59 | -0.33 |
| Affiliation (Indep) | New Assistant | 5.36 | 2.00 | -0.11 | -0.49 | 5.33 | 1.22 | -0.74 | 0.80 |
| Affiliation (Indep) | New Coworker | 5.74 | 2.27 | -0.11 | -0.84 | 5.09 | 1.52 | -0.87 | 0.06 |
| Affiliation (Indep) | Young Man Dark Alley | 4.48 | 2.41 | 0.12 | -0.92 | 4.67 | 1.57 | -0.26 | -0.82 |
| Affiliation (Indep) | Young Woman Dark Alley | 5.23 | 2.28 | -0.43 | -0.23 | 4.88 | 1.71 | -0.72 | -0.36 |
| Affiliation (Indep) | Elderly Man Dark Alley | 5.04 | 2.39 | -0.05 | -0.90 | 4.58 | 1.69 | -0.65 | -0.45 |
| Affiliation (Indep) | Elderly Woman Dark Alley | 5.68 | 2.22 | -0.23 | -0.49 | 4.89 | 1.63 | -0.60 | -0.52 |
| Mate Retention (Breakup) | Man | 2.87 | 1.96 | 0.35 | -0.71 | 4.43 | 1.57 | -0.37 | -0.58 |
| Mate Retention (Breakup) | Woman | 3.11 | 2.15 | 0.44 | -0.41 | 4.17 | 1.73 | -0.31 | -1.04 |
| Mate Retention (Breakup) | No Context | 3.14 | 2.18 | 0.42 | -0.64 | 4.12 | 1.73 | -0.21 | -0.96 |
| Mate Retention (Breakup) | Casual Sexual Partner | 4.28 | 2.47 | -0.18 | -1.11 | 4.50 | 1.62 | -0.58 | -0.50 |
| Mate Retention (Breakup) | Potential Life Partner | 3.36 | 2.24 | 0.17 | -1.00 | 4.66 | 1.48 | -0.38 | -0.51 |
| Mate Retention (Breakup) | New Boss | 2.35 | 2.09 | 0.70 | -0.65 | 3.25 | 1.77 | 0.39 | -0.86 |
| Mate Retention (Breakup) | New Assistant | 2.29 | 2.16 | 1.05 | 0.39 | 3.19 | 1.82 | 0.51 | -0.88 |
| Mate Retention (Breakup) | New Coworker | 2.62 | 2.21 | 0.97 | 0.44 | 3.41 | 1.67 | 0.28 | -0.96 |
| Mate Retention (Breakup) | Young Man Dark Alley | 3.15 | 1.91 | 0.44 | -0.34 | 3.78 | 1.61 | 0.01 | -0.76 |
| Mate Retention (Breakup) | Young Woman Dark Alley | 3.56 | 2.27 | 0.46 | -0.43 | 4.04 | 1.88 | -0.01 | -1.02 |
| Mate Retention (Breakup) | Elderly Man Dark Alley | 2.93 | 2.19 | 0.50 | -0.65 | 3.61 | 1.84 | 0.16 | -1.06 |
| Mate Retention (Breakup) | Elderly Woman Dark Alley | 2.69 | 2.01 | 0.91 | 0.95 | 3.61 | 1.84 | 0.27 | -1.05 |
| Disease Avoidance | Man | 3.98 | 2.68 | 0.32 | -0.82 | 4.59 | 1.73 | -0.37 | -0.86 |
| Disease Avoidance | Woman | 3.58 | 2.58 | 0.45 | -0.68 | 4.16 | 1.80 | -0.30 | -0.99 |
| Disease Avoidance | No Context | 4.14 | 2.62 | 0.13 | -0.85 | 4.20 | 1.80 | -0.22 | -1.00 |
| Disease Avoidance | Casual Sexual Partner | 7.35 | 2.75 | -0.77 | -0.47 | 6.07 | 1.59 | -1.91 | 2.72 |
| Disease Avoidance | Potential Life Partner | 4.15 | 2.54 | 0.15 | -0.94 | 4.81 | 1.53 | -0.62 | -0.20 |
| Disease Avoidance | New Boss | 3.91 | 2.57 | 0.25 | -0.78 | 3.83 | 1.83 | -0.02 | -1.10 |
| Disease Avoidance | New Assistant | 4.43 | 2.36 | 0.39 | -0.58 | 4.19 | 1.80 | -0.13 | -1.05 |
| Disease Avoidance | New Coworker | 4.26 | 2.55 | 0.04 | -0.96 | 4.15 | 1.78 | -0.23 | -0.96 |
| Disease Avoidance | Young Man Dark Alley | 5.01 | 2.82 | -0.13 | -0.98 | 4.47 | 1.84 | -0.43 | -0.85 |
| Disease Avoidance | Young Woman Dark Alley | 4.96 | 2.76 | 0.12 | -1.07 | 4.42 | 1.78 | -0.42 | -0.79 |
| Disease Avoidance | Elderly Man Dark Alley | 4.95 | 2.96 | -0.13 | -1.09 | 4.71 | 1.84 | -0.48 | -0.86 |
| Disease Avoidance | Elderly Woman Dark Alley | 4.60 | 2.89 | -0.07 | -1.01 | 3.82 | 1.96 | 0.03 | -1.26 |
| Status | Man | 4.78 | 2.40 | 0.12 | -0.54 | 4.87 | 1.63 | -0.50 | -0.76 |
| Status | Woman | 5.09 | 2.71 | -0.03 | -1.01 | 4.81 | 1.60 | -0.47 | -0.62 |
| Status | No Context | 4.50 | 2.74 | 0.20 | -0.81 | 4.53 | 1.77 | -0.50 | -0.76 |
| Status | Casual Sexual Partner | 3.45 | 2.42 | 0.24 | -0.88 | 4.09 | 1.86 | -0.29 | -1.12 |
| Status | Potential Life Partner | 3.93 | 2.50 | 0.19 | -0.73 | 4.67 | 1.60 | -0.67 | -0.14 |
| Status | New Boss | 6.34 | 2.34 | 0.06 | -1.03 | 5.41 | 1.56 | -1.06 | 0.77 |
| Status | New Assistant | 6.37 | 2.51 | -0.17 | -0.87 | 5.66 | 1.37 | -1.01 | 0.46 |
| Status | New Coworker | 5.98 | 2.94 | -0.29 | -0.97 | 5.04 | 1.78 | -0.75 | -0.41 |
| Status | Young Man Dark Alley | 3.77 | 2.61 | 0.18 | -1.06 | 3.88 | 1.87 | -0.01 | -1.15 |
| Status | Young Woman Dark Alley | 3.52 | 2.45 | 0.38 | -0.34 | 3.82 | 1.79 | -0.01 | -0.91 |
| Status | Elderly Man Dark Alley | 3.56 | 2.69 | 0.46 | -0.71 | 3.70 | 1.82 | 0.14 | -1.09 |
| Status | Elderly Woman Dark Alley | 4.13 | 2.84 | 0.35 | -0.79 | 3.84 | 1.89 | -0.06 | -1.28 |
| Affiliation (Group) | Man | 4.65 | 2.32 | 0.32 | -0.48 | 4.81 | 1.51 | -0.43 | -0.52 |
| Affiliation (Group) | Woman | 4.95 | 2.33 | -0.25 | -0.59 | 4.70 | 1.40 | -0.97 | 0.43 |
| Affiliation (Group) | No Context | 5.08 | 2.10 | 0.06 | -0.65 | 4.61 | 1.51 | -0.51 | -0.31 |
| Affiliation (Group) | Casual Sexual Partner | 3.58 | 2.09 | 0.20 | -0.53 | 4.27 | 1.64 | -0.55 | -0.89 |
| Affiliation (Group) | Potential Life Partner | 4.60 | 2.15 | 0.18 | -1.05 | 4.63 | 1.34 | -0.49 | -0.03 |
| Affiliation (Group) | New Boss | 6.07 | 1.92 | -0.33 | -0.27 | 4.95 | 1.62 | -0.62 | -0.41 |
| Affiliation (Group) | New Assistant | 6.06 | 2.48 | -0.33 | -0.79 | 5.37 | 1.39 | -0.78 | 0.05 |
| Affiliation (Group) | New Coworker | 5.67 | 2.41 | -0.07 | -1.02 | 4.77 | 1.74 | -0.62 | -0.65 |
| Affiliation (Group) | Young Man Dark Alley | 5.23 | 2.40 | -0.08 | -1.17 | 4.66 | 1.58 | -0.46 | -0.43 |
| Affiliation (Group) | Young Woman Dark Alley | 4.58 | 2.17 | -0.22 | -0.87 | 4.29 | 1.68 | -0.46 | -0.68 |
| Affiliation (Group) | Elderly Man Dark Alley | 5.65 | 2.10 | -0.05 | -0.82 | 4.60 | 1.49 | -0.45 | -0.41 |
| Affiliation (Group) | Elderly Woman Dark Alley | 5.66 | 2.05 | 0.00 | -0.55 | 4.67 | 1.56 | -0.62 | -0.22 |
| Mate Seeking | Man | 3.88 | 2.76 | 0.34 | -0.82 | 4.88 | 1.72 | -0.73 | -0.34 |
| Mate Seeking | Woman | 3.75 | 3.06 | 0.41 | -0.88 | 4.15 | 1.98 | -0.18 | -1.34 |
| Mate Seeking | No Context | 4.04 | 2.70 | 0.11 | -0.87 | 4.55 | 1.77 | -0.52 | -0.68 |
| Mate Seeking | Casual Sexual Partner | 4.65 | 2.86 | -0.12 | -1.25 | 5.37 | 1.55 | -1.23 | 1.01 |
| Mate Seeking | Potential Life Partner | 3.71 | 2.94 | 0.31 | -1.12 | 5.12 | 1.78 | -0.97 | 0.05 |
| Mate Seeking | New Boss | 2.49 | 2.35 | 0.67 | -0.77 | 3.05 | 1.87 | 0.45 | -1.01 |
| Mate Seeking | New Assistant | 2.32 | 2.53 | 0.94 | -0.27 | 3.20 | 2.02 | 0.48 | -1.14 |
| Mate Seeking | New Coworker | 2.90 | 2.60 | 0.75 | -0.16 | 3.52 | 1.86 | 0.15 | -1.18 |
| Mate Seeking | Young Man Dark Alley | 2.86 | 2.59 | 0.81 | -0.05 | 3.79 | 1.82 | 0.12 | -1.00 |
| Mate Seeking | Young Woman Dark Alley | 3.31 | 2.80 | 0.60 | -0.65 | 4.01 | 2.02 | -0.10 | -1.29 |
| Mate Seeking | Elderly Man Dark Alley | 2.77 | 2.49 | 0.82 | -0.16 | 3.31 | 1.99 | 0.40 | -1.16 |
| Mate Seeking | Elderly Woman Dark Alley | 3.17 | 2.69 | 0.70 | -0.57 | 3.71 | 2.05 | 0.11 | -1.35 |
| Mate Retention (Gen) | Man | 7.20 | 1.88 | -0.49 | -0.43 | 6.04 | 1.12 | -1.49 | 3.08 |
| Mate Retention (Gen) | Woman | 6.90 | 2.08 | -0.51 | -0.37 | 5.60 | 1.50 | -1.13 | 0.57 |
| Mate Retention (Gen) | No Context | 6.59 | 2.27 | -0.35 | -0.66 | 5.53 | 1.47 | -1.21 | 1.17 |
| Mate Retention (Gen) | Casual Sexual Partner | 6.73 | 2.45 | -0.71 | -0.13 | 5.73 | 1.45 | -1.53 | 2.14 |
| Mate Retention (Gen) | Potential Life Partner | 8.15 | 2.06 | -0.93 | -0.46 | 6.50 | 0.86 | -1.58 | 1.39 |
| Mate Retention (Gen) | New Boss | 5.20 | 2.14 | 0.12 | -0.46 | 4.10 | 1.97 | -0.28 | -1.17 |
| Mate Retention (Gen) | New Assistant | 4.96 | 2.18 | 0.22 | -0.48 | 4.35 | 1.88 | -0.29 | -1.02 |
| Mate Retention (Gen) | New Coworker | 4.90 | 2.19 | -0.03 | -0.55 | 4.15 | 1.73 | -0.28 | -0.98 |
| Mate Retention (Gen) | Young Man Dark Alley | 4.81 | 2.33 | -0.05 | -0.61 | 4.27 | 1.77 | -0.50 | -0.82 |
| Mate Retention (Gen) | Young Woman Dark Alley | 5.79 | 2.33 | -0.25 | -0.64 | 4.74 | 1.86 | -0.63 | -0.71 |
| Mate Retention (Gen) | Elderly Man Dark Alley | 5.03 | 2.06 | -0.05 | -0.80 | 4.04 | 1.94 | -0.20 | -1.20 |
| Mate Retention (Gen) | Elderly Woman Dark Alley | 4.60 | 2.30 | 0.35 | -0.67 | 4.24 | 1.87 | -0.28 | -1.13 |

# Motive Prioritization Across Social Contexts: Rating Scale Heat Map


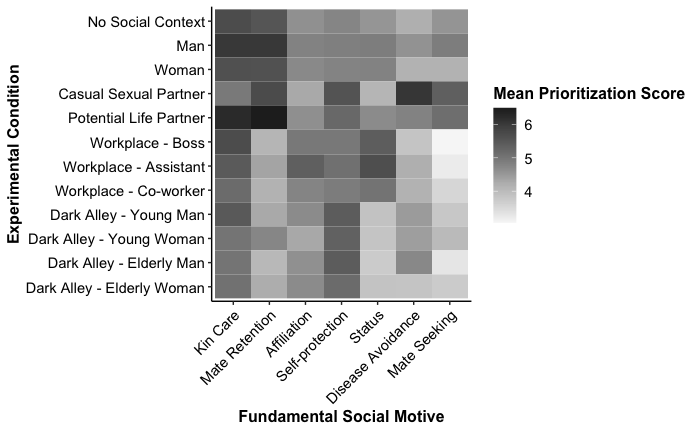


# Variability of Motive Prioritization Across Conditions (Standard Deviations around Grand Mean)

| **Motive** | **Forced-Choice Measure** | | | | | **Rating Scale Measure** | | | | |
| --- | --- | --- | --- | --- | --- | --- | --- | --- | --- | --- |
|  | **Total** | **Gender** | **Dating** | **Workplace** | **Dark Alley** | **Total** | **Gender** | **Dating** | **Workplace** | **Dark Alley** |
| Kin Care (Fam) | 0.58 | 0.24 | 1.53 | 0.28 | 0.22 | 0.43 | 0.29 | 0.93 | 0.27 | 0.22 |
| Affiliation (Excl) | 0.71 | 0.09 | 0.13 | 0.19 | 0.37 | 0.25 | 0.14 | 0.37 | 0.35 | 0.09 |
| Self-Protection | 0.74 | 0.24 | 0.72 | 0.01 | 0.16 | 0.27 | 0.05 | 0.25 | 0.10 | 0.12 |
| Kin Care (Child) | 0.52 | 0.32 | 1.20 | 0.26 | 0.38 | 0.40 | 0.42 | 0.93 | 0.26 | 0.30 |
| Affiliation (Indep) | 0.46 | 0.69 | 0.37 | 0.42 | 0.50 | 0.37 | 0.24 | 0.37 | 0.28 | 0.15 |
| Mate Retention (Breakup) | 0.55 | 0.17 | 0.65 | 0.17 | 0.37 | 0.50 | 0.19 | 0.11 | 0.11 | 0.20 |
| Disease Avoidance | 0.97 | 0.29 | 2.26 | 0.27 | 0.19 | 0.60 | 0.30 | 0.89 | 0.20 | 0.38 |
| Status | 1.10 | 0.22 | 0.34 | 0.22 | 0.28 | 0.66 | 0.04 | 0.41 | 0.31 | 0.07 |
| Affiliation (Group) | 0.73 | 0.21 | 0.72 | 0.23 | 0.51 | 0.29 | 0.08 | 0.26 | 0.31 | 0.18 |
| Mate Seeking | 0.70 | 0.09 | 0.67 | 0.30 | 0.25 | 0.77 | 0.51 | 0.18 | 0.24 | 0.29 |
| Mate Retention (Gen) | 1.17 | 0.21 | 1.00 | 0.16 | 0.52 | 0.88 | 0.31 | 0.54 | 0.13 | 0.30 |

# Specific Conditions in which each Motive was Prioritized

## Forced-Choice Measure

|  | **Kin Care (Fam.)** | | | **Mate Retention (Gen.)** | | | **Affiliation (Group)** | | | **Self-Protection** | | | **Status** | | | **Disease Avoidance** | | | **Mate Seeking** | | |
| --- | --- | --- | --- | --- | --- | --- | --- | --- | --- | --- | --- | --- | --- | --- | --- | --- | --- | --- | --- | --- | --- |
| *Predictors* | *std. Beta* | *standardized CI* | *p* | *std. Beta* | *standardized CI* | *p* | *std. Beta* | *standardized CI* | *p* | *std. Beta* | *standardized CI* | *p* | *std. Beta* | *standardized CI* | *p* | *std. Beta* | *standardized CI* | *p* | *std. Beta* | *standardized CI* | *p* |
| Intercept (Grand Mean) | 0.00 | -0.07 – 0.07 | **<0.001** | -0.06 | -0.13 – 0.01 | **<0.001** | 0.01 | -0.06 – 0.08 | **<0.001** | 0.08 | 0.01 – 0.15 | **<0.001** | 0.01 | -0.06 – 0.08 | **<0.001** | 0.04 | -0.03 – 0.10 | **<0.001** | -0.05 | -0.12 – 0.02 | **<0.001** |
| No Context | -0.01 | -0.14 – 0.13 | 0.907 | 0.28 | 0.16 – 0.41 | **<0.001** | -0.03 | -0.16 – 0.10 | 0.541 | -0.37 | -0.50 – -0.24 | **<0.001** | -0.04 | -0.17 – 0.09 | 0.381 | -0.17 | -0.30 – -0.04 | **0.001** | 0.26 | 0.12 – 0.39 | **<0.001** |
| Man | 0.19 | -0.05 – 0.44 | **0.039** | 0.53 | 0.31 – 0.76 | **<0.001** | -0.22 | -0.46 – 0.02 | **0.017** | -0.31 | -0.54 – -0.07 | **0.001** | 0.06 | -0.18 – 0.29 | 0.527 | -0.23 | -0.46 – 0.01 | **0.014** | 0.20 | -0.04 – 0.44 | **0.032** |
| Woman | 0.05 | -0.19 – 0.29 | 0.580 | 0.41 | 0.18 – 0.63 | **<0.001** | -0.09 | -0.33 – 0.15 | 0.347 | -0.45 | -0.69 – -0.21 | **<0.001** | 0.17 | -0.06 – 0.40 | 0.061 | -0.37 | -0.61 – -0.13 | **<0.001** | 0.15 | -0.09 – 0.40 | 0.100 |
| Casual Sexual Partner | -0.70 | -0.95 – -0.46 | **<0.001** | 0.34 | 0.12 – 0.57 | **<0.001** | -0.69 | -0.93 – -0.45 | **<0.001** | 0.14 | -0.10 – 0.38 | 0.129 | -0.42 | -0.65 – -0.18 | **<0.001** | 0.98 | 0.74 – 1.22 | **<0.001** | 0.48 | 0.24 – 0.72 | **<0.001** |
| Potential Life Partner | 0.22 | -0.02 – 0.46 | **0.017** | 0.92 | 0.70 – 1.14 | **<0.001** | -0.24 | -0.47 – -0.01 | **0.008** | -0.29 | -0.52 – -0.06 | **0.001** | -0.25 | -0.48 – -0.02 | **0.006** | -0.17 | -0.40 – 0.07 | 0.065 | 0.14 | -0.10 – 0.38 | 0.128 |
| New Boss | 0.22 | -0.02 – 0.47 | **0.020** | -0.29 | -0.52 – -0.06 | **0.001** | 0.41 | 0.17 – 0.65 | **<0.001** | -0.05 | -0.29 – 0.18 | 0.556 | 0.62 | 0.38 – 0.86 | **<0.001** | -0.25 | -0.49 – -0.01 | **0.007** | -0.30 | -0.54 – -0.05 | **0.002** |
| New Assistant | 0.04 | -0.20 – 0.29 | 0.644 | -0.39 | -0.61 – -0.16 | **<0.001** | 0.40 | 0.16 – 0.64 | **<0.001** | -0.07 | -0.30 – 0.17 | 0.475 | 0.63 | 0.40 – 0.86 | **<0.001** | -0.06 | -0.30 – 0.17 | 0.482 | -0.36 | -0.60 – -0.12 | **<0.001** |
| New Coworker | -0.01 | -0.25 – 0.23 | 0.939 | -0.41 | -0.63 – -0.19 | **<0.001** | 0.23 | -0.01 – 0.46 | **0.012** | -0.06 | -0.29 – 0.18 | 0.524 | 0.49 | 0.26 – 0.72 | **<0.001** | -0.12 | -0.36 – 0.11 | 0.172 | -0.15 | -0.39 – 0.09 | 0.102 |
| Young Man Dark Alley | 0.13 | -0.11 – 0.38 | 0.164 | -0.45 | -0.68 – -0.22 | **<0.001** | 0.04 | -0.21 – 0.28 | 0.701 | 0.40 | 0.16 – 0.64 | **<0.001** | -0.30 | -0.54 – -0.07 | **0.001** | 0.14 | -0.10 – 0.38 | 0.122 | -0.16 | -0.41 – 0.08 | 0.083 |
| Young Woman Dark Alley | -0.05 | -0.29 – 0.20 | 0.622 | -0.05 | -0.27 – 0.18 | 0.584 | -0.25 | -0.49 – -0.01 | **0.007** | 0.32 | 0.08 – 0.55 | **0.001** | -0.39 | -0.63 – -0.16 | **<0.001** | 0.13 | -0.11 – 0.36 | 0.168 | -0.00 | -0.24 – 0.24 | 0.968 |
| Elderly Man Dark Alley | -0.08 | -0.32 – 0.16 | 0.388 | -0.36 | -0.58 – -0.13 | **<0.001** | 0.22 | -0.02 – 0.46 | **0.018** | 0.44 | 0.20 – 0.68 | **<0.001** | -0.38 | -0.62 – -0.15 | **<0.001** | 0.12 | -0.11 – 0.36 | 0.183 | -0.20 | -0.44 – 0.04 | **0.033** |
| Elderly Woman Dark Alley | -0.02 | -0.26 – 0.22 | 0.832 | -0.54 | -0.76 – -0.32 | **<0.001** | 0.22 | -0.01 – 0.46 | **0.014** | 0.30 | 0.06 – 0.53 | **0.001** | -0.18 | -0.41 – 0.05 | **0.049** | -0.00 | -0.24 – 0.23 | 0.965 | -0.06 | -0.29 – 0.18 | 0.548 |
| Observations | 1502 | | | 1502 | | | 1502 | | | 1502 | | | 1502 | | | 1502 | | | 1502 | | |
| R^2^ / R^2^ adjusted | 0.043 / 0.036 | | | 0.182 / 0.176 | | | 0.075 / 0.068 | | | 0.095 / 0.088 | | | 0.113 / 0.107 | | | 0.092 / 0.086 | | | 0.056 / 0.049 | | |

|  | **Kin Care (Children)** | | | **Affiliation (Excl. Concern)** | | | **Affiliation (Indep.)** | | | **Mate Retention (Breakup)** | | |
| --- | --- | --- | --- | --- | --- | --- | --- | --- | --- | --- | --- | --- |
| *Predictors* | *std. Beta* | *standardized CI* | *p* | *std. Beta* | *standardized CI* | *p* | *std. Beta* | *standardized CI* | *p* | *std. Beta* | *standardized CI* | *p* |
| Intercept (Grand Mean) | -0.01 | -0.08 – 0.06 | **<0.001** | 0.03 | -0.04 – 0.10 | **<0.001** | -0.03 | -0.10 – 0.05 | **<0.001** | -0.01 | -0.08 – 0.06 | **<0.001** |
| No Context | 0.04 | -0.10 – 0.18 | 0.455 | -0.16 | -0.29 – -0.03 | **0.002** | 0.12 | -0.02 – 0.25 | **0.026** | 0.05 | -0.08 – 0.19 | 0.323 |
| Man | 0.20 | -0.05 – 0.44 | **0.036** | -0.42 | -0.66 – -0.18 | **<0.001** | -0.05 | -0.30 – 0.19 | 0.577 | -0.07 | -0.32 – 0.17 | 0.442 |
| Woman | 0.04 | -0.21 – 0.28 | 0.687 | -0.36 | -0.60 – -0.12 | **<0.001** | 0.38 | 0.14 – 0.63 | **<0.001** | 0.04 | -0.21 – 0.28 | 0.690 |
| Casual Sexual Partner | -0.47 | -0.71 – -0.22 | **<0.001** | -0.34 | -0.58 – -0.10 | **<0.001** | -0.02 | -0.26 – 0.23 | 0.866 | 0.57 | 0.32 – 0.81 | **<0.001** |
| Potential Life Partner | 0.14 | -0.10 – 0.38 | 0.146 | -0.43 | -0.66 – -0.19 | **<0.001** | -0.25 | -0.49 – -0.01 | **0.008** | 0.15 | -0.09 – 0.39 | 0.105 |
| New Boss | 0.04 | -0.21 – 0.29 | 0.686 | 0.06 | -0.18 – 0.30 | 0.528 | -0.16 | -0.41 – 0.09 | 0.092 | -0.31 | -0.55 – -0.06 | **0.001** |
| New Assistant | -0.13 | -0.37 – 0.12 | 0.184 | 0.24 | -0.00 – 0.47 | **0.010** | 0.04 | -0.20 – 0.29 | 0.640 | -0.33 | -0.58 – -0.09 | **<0.001** |
| New Coworker | -0.11 | -0.35 – 0.13 | 0.235 | 0.13 | -0.10 – 0.37 | 0.142 | 0.21 | -0.03 – 0.45 | **0.024** | -0.19 | -0.43 – 0.05 | **0.045** |
| Young Man Dark Alley | 0.23 | -0.02 – 0.48 | **0.017** | 0.31 | 0.07 – 0.55 | **0.001** | -0.35 | -0.59 – -0.10 | **<0.001** | 0.05 | -0.19 – 0.30 | 0.577 |
| Young Woman Dark Alley | 0.04 | -0.20 – 0.29 | 0.642 | 0.08 | -0.15 – 0.32 | 0.373 | -0.01 | -0.26 – 0.23 | 0.885 | 0.24 | -0.00 – 0.48 | **0.010** |
| Elderly Man Dark Alley | 0.07 | -0.17 – 0.32 | 0.459 | 0.43 | 0.19 – 0.67 | **<0.001** | -0.10 | -0.34 – 0.15 | 0.303 | -0.04 | -0.29 – 0.20 | 0.633 |
| Elderly Woman Dark Alley | -0.09 | -0.34 – 0.15 | 0.316 | 0.46 | 0.23 – 0.70 | **<0.001** | 0.18 | -0.06 – 0.43 | **0.049** | -0.16 | -0.39 – 0.08 | 0.095 |
| Observations | 1502 | | | 1502 | | | 1502 | | | 1502 | | |
| R^2^ / R^2^ adjusted | 0.025 / 0.018 | | | 0.085 / 0.078 | | | 0.033 / 0.025 | | | 0.045 / 0.038 | | |

## Rating Scale Measure

|  | **Kin Care (Fam.)** | | | **Mate Retention (Gen.)** | | | **Affiliation (Group)** | | | **Self-Protection** | | | **Status** | | | **Disease Avoidance** | | | **Mate Seeking** | | |
| --- | --- | --- | --- | --- | --- | --- | --- | --- | --- | --- | --- | --- | --- | --- | --- | --- | --- | --- | --- | --- | --- |
| *Predictors* | *std. Beta* | *standardized CI* | *p* | *std. Beta* | *standardized CI* | *p* | *std. Beta* | *standardized CI* | *p* | *std. Beta* | *standardized CI* | *p* | *std. Beta* | *standardized CI* | *p* | *std. Beta* | *standardized CI* | *p* | *std. Beta* | *standardized CI* | *p* |
| Intercept (Grand Mean) | -0.03 | -0.10 – 0.04 | **<0.001** | -0.07 | -0.14 – -0.00 | **<0.001** | 0.01 | -0.06 – 0.08 | **<0.001** | 0.05 | -0.02 – 0.12 | **<0.001** | -0.00 | -0.07 – 0.07 | **<0.001** | 0.03 | -0.04 – 0.10 | **<0.001** | -0.05 | -0.12 – 0.02 | **<0.001** |
| No Context | 0.16 | 0.02 – 0.29 | **0.003** | 0.33 | 0.20 – 0.45 | **<0.001** | -0.05 | -0.19 – 0.08 | 0.321 | -0.23 | -0.36 – -0.09 | **<0.001** | 0.00 | -0.13 – 0.13 | 0.963 | -0.13 | -0.27 – -0.00 | **0.009** | 0.25 | 0.12 – 0.38 | **<0.001** |
| Man | 0.38 | 0.14 – 0.62 | **<0.001** | 0.61 | 0.39 – 0.84 | **<0.001** | 0.07 | -0.17 – 0.32 | 0.438 | -0.17 | -0.41 – 0.08 | 0.082 | 0.19 | -0.05 – 0.43 | **0.039** | 0.07 | -0.17 – 0.31 | 0.434 | 0.42 | 0.18 – 0.65 | **<0.001** |
| Woman | 0.11 | -0.13 – 0.35 | 0.232 | 0.37 | 0.14 – 0.59 | **<0.001** | 0.00 | -0.24 – 0.25 | 0.983 | -0.21 | -0.46 – 0.03 | **0.027** | 0.16 | -0.08 – 0.39 | 0.090 | -0.16 | -0.40 – 0.08 | 0.092 | 0.05 | -0.18 – 0.28 | 0.585 |
| Casual Sexual Partner | -0.33 | -0.57 – -0.09 | **<0.001** | 0.44 | 0.22 – 0.67 | **<0.001** | -0.28 | -0.52 – -0.03 | **0.004** | 0.29 | 0.04 – 0.53 | **0.003** | -0.24 | -0.48 – -0.00 | **0.009** | 0.88 | 0.64 – 1.12 | **<0.001** | 0.67 | 0.43 – 0.90 | **<0.001** |
| Potential Life Partner | 0.52 | 0.28 – 0.75 | **<0.001** | 0.87 | 0.65 – 1.09 | **<0.001** | -0.04 | -0.28 – 0.20 | 0.664 | 0.06 | -0.18 – 0.30 | 0.525 | 0.08 | -0.15 – 0.31 | 0.378 | 0.19 | -0.04 – 0.43 | **0.035** | 0.54 | 0.31 – 0.77 | **<0.001** |
| New Boss | 0.16 | -0.08 – 0.41 | 0.082 | -0.47 | -0.69 – -0.24 | **<0.001** | 0.16 | -0.08 – 0.41 | 0.089 | -0.10 | -0.35 – 0.15 | 0.289 | 0.49 | 0.25 – 0.73 | **<0.001** | -0.34 | -0.58 – -0.09 | **<0.001** | -0.51 | -0.74 – -0.27 | **<0.001** |
| New Assistant | -0.00 | -0.24 – 0.24 | 0.983 | -0.33 | -0.55 – -0.11 | **<0.001** | 0.44 | 0.19 – 0.68 | **<0.001** | -0.03 | -0.27 – 0.22 | 0.790 | 0.63 | 0.39 – 0.86 | **<0.001** | -0.14 | -0.38 – 0.10 | 0.123 | -0.43 | -0.66 – -0.20 | **<0.001** |
| New Coworker | -0.18 | -0.42 – 0.05 | **0.047** | -0.44 | -0.66 – -0.22 | **<0.001** | 0.05 | -0.19 – 0.29 | 0.591 | -0.14 | -0.39 – 0.10 | 0.124 | 0.28 | 0.05 – 0.52 | **0.002** | -0.16 | -0.40 – 0.07 | 0.073 | -0.27 | -0.50 – -0.04 | **0.003** |
| Young Man Dark Alley | 0.01 | -0.23 – 0.25 | 0.915 | -0.37 | -0.60 – -0.15 | **<0.001** | -0.02 | -0.27 – 0.23 | 0.838 | 0.19 | -0.06 – 0.44 | 0.051 | -0.36 | -0.60 – -0.12 | **<0.001** | 0.01 | -0.23 – 0.25 | 0.925 | -0.13 | -0.37 – 0.10 | 0.146 |
| Young Woman Dark Alley | -0.29 | -0.53 – -0.05 | **0.002** | -0.11 | -0.33 – 0.11 | 0.194 | -0.26 | -0.50 – -0.02 | **0.006** | 0.13 | -0.11 – 0.38 | 0.159 | -0.39 | -0.63 – -0.16 | **<0.001** | -0.02 | -0.25 – 0.22 | 0.849 | -0.02 | -0.25 – 0.21 | 0.803 |
| Elderly Man Dark Alley | -0.28 | -0.52 – -0.04 | **0.003** | -0.50 | -0.73 – -0.28 | **<0.001** | -0.06 | -0.31 – 0.19 | 0.535 | 0.19 | -0.06 – 0.43 | **0.048** | -0.45 | -0.69 – -0.22 | **<0.001** | 0.14 | -0.10 – 0.38 | 0.127 | -0.38 | -0.61 – -0.15 | **<0.001** |
| Elderly Woman Dark Alley | -0.25 | -0.49 – -0.02 | **0.006** | -0.39 | -0.61 – -0.17 | **<0.001** | -0.02 | -0.26 – 0.22 | 0.852 | 0.02 | -0.22 – 0.26 | 0.802 | -0.38 | -0.61 – -0.14 | **<0.001** | -0.34 | -0.58 – -0.11 | **<0.001** | -0.18 | -0.41 – 0.05 | **0.048** |
| Observations | 1500 | | | 1500 | | | 1499 | | | 1499 | | | 1499 | | | 1499 | | | 1497 | | |
| R^2^ / R^2^ adjusted | 0.060 / 0.053 | | | 0.194 / 0.188 | | | 0.026 / 0.018 | | | 0.030 / 0.023 | | | 0.096 / 0.090 | | | 0.078 / 0.072 | | | 0.121 / 0.115 | | |

|  | **Kin Care (Children)** | | | **Affiliation (Excl. Concern)** | | | **Affiliation (Indep.)** | | | **Mate Retention (Breakup)** | | |
| --- | --- | --- | --- | --- | --- | --- | --- | --- | --- | --- | --- | --- |
| *Predictors* | *std. Beta* | *standardized CI* | *p* | *std. Beta* | *standardized CI* | *p* | *std. Beta* | *standardized CI* | *p* | *std. Beta* | *standardized CI* | *p* |
| Intercept (Grand Mean) | -0.01 | -0.08 – 0.06 | **<0.001** | 0.03 | -0.05 – 0.10 | **<0.001** | -0.04 | -0.11 – 0.04 | **<0.001** | -0.03 | -0.10 – 0.04 | **<0.001** |
| No Context | 0.05 | -0.08 – 0.19 | 0.325 | -0.13 | -0.27 – 0.01 | **0.014** | 0.16 | 0.03 – 0.30 | **0.002** | 0.13 | -0.01 – 0.26 | **0.014** |
| Man | 0.35 | 0.10 – 0.59 | **<0.001** | -0.01 | -0.26 – 0.23 | 0.888 | 0.18 | -0.06 – 0.43 | **0.049** | 0.30 | 0.06 – 0.54 | **0.001** |
| Woman | 0.01 | -0.23 – 0.25 | 0.924 | -0.14 | -0.39 – 0.11 | 0.143 | 0.42 | 0.18 – 0.66 | **<0.001** | 0.15 | -0.09 – 0.40 | 0.097 |
| Casual Sexual Partner | -0.39 | -0.64 – -0.15 | **<0.001** | -0.28 | -0.53 – -0.03 | **0.004** | -0.02 | -0.27 – 0.22 | 0.810 | 0.34 | 0.10 – 0.58 | **<0.001** |
| Potential Life Partner | 0.35 | 0.11 – 0.59 | **<0.001** | 0.05 | -0.19 – 0.29 | 0.603 | 0.33 | 0.10 – 0.57 | **<0.001** | 0.43 | 0.20 – 0.67 | **<0.001** |
| New Boss | 0.09 | -0.16 – 0.34 | 0.339 | 0.07 | -0.18 – 0.32 | 0.491 | -0.23 | -0.47 – 0.02 | **0.016** | -0.37 | -0.61 – -0.12 | **<0.001** |
| New Assistant | -0.05 | -0.29 – 0.19 | 0.596 | 0.36 | 0.11 – 0.60 | **<0.001** | 0.15 | -0.09 – 0.39 | 0.114 | -0.40 | -0.64 – -0.16 | **<0.001** |
| New Coworker | -0.20 | -0.44 – 0.04 | **0.030** | -0.07 | -0.32 – 0.17 | 0.431 | -0.02 | -0.26 – 0.22 | 0.851 | -0.28 | -0.52 – -0.04 | **0.003** |
| Young Man Dark Alley | 0.18 | -0.07 – 0.43 | 0.059 | 0.07 | -0.18 – 0.32 | 0.450 | -0.30 | -0.55 – -0.06 | **0.002** | -0.07 | -0.31 – 0.18 | 0.479 |
| Young Woman Dark Alley | -0.13 | -0.37 – 0.12 | 0.174 | -0.04 | -0.29 – 0.20 | 0.665 | -0.16 | -0.40 – 0.08 | 0.085 | 0.08 | -0.16 – 0.32 | 0.388 |
| Elderly Man Dark Alley | -0.05 | -0.29 – 0.20 | 0.635 | 0.07 | -0.18 – 0.31 | 0.492 | -0.37 | -0.61 – -0.12 | **<0.001** | -0.16 | -0.41 – 0.08 | 0.082 |
| Elderly Woman Dark Alley | -0.21 | -0.45 – 0.03 | **0.022** | 0.06 | -0.18 – 0.31 | 0.494 | -0.15 | -0.39 – 0.09 | 0.099 | -0.16 | -0.40 – 0.08 | 0.082 |
| Observations | 1496 | | | 1500 | | | 1499 | | | 1496 | | |
| R^2^ / R^2^ adjusted | 0.037 / 0.030 | | | 0.021 / 0.013 | | | 0.050 / 0.043 | | | 0.061 / 0.054 | | |

# Alternative Analysis: Prioritized motives relative to other motives within a condition

## Forced-Choice Measure

### Study 1: Gender

| **c** | **No Context (All Studies)** | | | **Man** | | | **Woman** | | |
| --- | --- | --- | --- | --- | --- | --- | --- | --- | --- |
| *Predictors* | *std. Beta* | *standardized CI* | *p* | *std. Beta* | *standardized CI* | *p* | *std. Beta* | *standardized CI* | *p* |
| Intercept (Grand Mean) | -0.00 | -0.03 – 0.03 | **<0.001** | -0.00 | -0.07 – 0.07 | **<0.001** | -0.00 | -0.07 – 0.07 | **<0.001** |
| Kin Care (Fam.) | 0.72 | 0.61 – 0.83 | **<0.001** | 0.87 | 0.66 – 1.08 | **<0.001** | 0.74 | 0.52 – 0.95 | **<0.001** |
| Kin Care (Child.) | 0.52 | 0.41 – 0.63 | **<0.001** | 0.67 | 0.46 – 0.88 | **<0.001** | 0.50 | 0.28 – 0.71 | **<0.001** |
| Mate Retention (Gen.) | 0.59 | 0.48 – 0.70 | **<0.001** | 0.80 | 0.59 – 1.01 | **<0.001** | 0.68 | 0.46 – 0.89 | **<0.001** |
| Mate Retention (Breakup.) | -0.69 | -0.80 – -0.58 | **<0.001** | -0.78 | -0.99 – -0.57 | **<0.001** | -0.67 | -0.89 – -0.46 | **<0.001** |
| Affiliation (Group) | 0.03 | -0.08 – 0.14 | 0.505 | -0.13 | -0.34 – 0.08 | 0.112 | -0.02 | -0.23 – 0.20 | 0.829 |
| Affiliation (Ind.) | 0.20 | 0.09 – 0.30 | **<0.001** | 0.05 | -0.16 – 0.26 | 0.525 | 0.40 | 0.18 – 0.62 | **<0.001** |
| Affiliation (Excl.) | -0.45 | -0.56 – -0.34 | **<0.001** | -0.64 | -0.85 – -0.43 | **<0.001** | -0.58 | -0.80 – -0.37 | **<0.001** |
| Self-protection | -0.05 | -0.16 – 0.06 | 0.264 | 0.01 | -0.20 – 0.22 | 0.928 | -0.11 | -0.33 – 0.10 | 0.182 |
| Status | -0.19 | -0.30 – -0.08 | **<0.001** | -0.08 | -0.29 – 0.13 | 0.318 | 0.03 | -0.18 – 0.25 | 0.698 |
| Disease Avoidance | -0.32 | -0.43 – -0.21 | **<0.001** | -0.37 | -0.58 – -0.16 | **<0.001** | -0.51 | -0.72 – -0.29 | **<0.001** |
| Mate Seeking | -0.36 | -0.47 – -0.25 | **<0.001** | -0.41 | -0.62 – -0.20 | **<0.001** | -0.45 | -0.66 – -0.23 | **<0.001** |
| Observations | 4488 | | | 1089 | | | 1089 | | |
| R^2^ / R^2^ adjusted | 0.193 / 0.191 | | | 0.291 / 0.285 | | | 0.244 / 0.237 | | |

### Study 2: Dating

|  | **Casual Sexual Partner** | | | **Potential Life Partner** | | |
| --- | --- | --- | --- | --- | --- | --- |
| *Predictors* | *std. Beta* | *standardized CI* | *p* | *std. Beta* | *standardized CI* | *p* |
| Intercept (Grand Mean) | -0.00 | -0.07 – 0.07 | **<0.001** | 0.00 | -0.06 – 0.06 | **<0.001** |
| Kin Care (Fam.) | 0.10 | -0.12 – 0.33 | 0.231 | 0.88 | 0.68 – 1.08 | **<0.001** |
| Kin Care (Child.) | -0.01 | -0.23 – 0.21 | 0.898 | 0.60 | 0.40 – 0.80 | **<0.001** |
| Mate Retention (Gen.) | 0.63 | 0.40 – 0.85 | **<0.001** | 1.12 | 0.92 – 1.32 | **<0.001** |
| Mate Retention (Breakup.) | -0.26 | -0.48 – -0.04 | **0.002** | -0.59 | -0.79 – -0.39 | **<0.001** |
| Affiliation (Group) | -0.51 | -0.73 – -0.29 | **<0.001** | -0.14 | -0.34 – 0.06 | 0.066 |
| Affiliation (Ind.) | 0.08 | -0.14 – 0.30 | 0.347 | -0.11 | -0.31 – 0.09 | 0.164 |
| Affiliation (Excl.) | -0.58 | -0.80 – -0.35 | **<0.001** | -0.63 | -0.83 – -0.44 | **<0.001** |
| Self-protection | 0.39 | 0.16 – 0.61 | **<0.001** | 0.02 | -0.18 – 0.22 | 0.788 |
| Status | -0.56 | -0.78 – -0.34 | **<0.001** | -0.38 | -0.58 – -0.18 | **<0.001** |
| Disease Avoidance | 0.85 | 0.63 – 1.07 | **<0.001** | -0.31 | -0.50 – -0.11 | **<0.001** |
| Mate Seeking | -0.13 | -0.35 – 0.10 | 0.146 | -0.46 | -0.66 – -0.26 | **<0.001** |
| Observations | 1078 | | | 1133 | | |
| R^2^ / R^2^ adjusted | 0.207 / 0.199 | | | 0.329 / 0.323 | | |

### Study 3: Workplace

|  | **New Assistant** | | | **New Boss** | | | **New Coworker** | | |
| --- | --- | --- | --- | --- | --- | --- | --- | --- | --- |
| *Predictors* | *std. Beta* | *standardized CI* | *p* | *std. Beta* | *standardized CI* | *p* | *std. Beta* | *standardized CI* | *p* |
| Intercept (Grand Mean) | 0.00 | -0.07 – 0.07 | **<0.001** | 0.00 | -0.07 – 0.07 | **<0.001** | 0.00 | -0.07 – 0.07 | **<0.001** |
| Kin Care (Fam.) | 0.76 | 0.55 – 0.97 | **<0.001** | 0.91 | 0.70 – 1.12 | **<0.001** | 0.70 | 0.49 – 0.92 | **<0.001** |
| Kin Care (Child.) | 0.35 | 0.14 – 0.55 | **<0.001** | 0.52 | 0.31 – 0.72 | **<0.001** | 0.36 | 0.14 – 0.57 | **<0.001** |
| Mate Retention (Gen.) | -0.01 | -0.22 – 0.19 | 0.853 | 0.07 | -0.13 – 0.28 | 0.361 | -0.04 | -0.25 – 0.18 | 0.669 |
| Mate Retention (Breakup.) | -1.01 | -1.21 – -0.80 | **<0.001** | -0.98 | -1.19 – -0.77 | **<0.001** | -0.87 | -1.09 – -0.66 | **<0.001** |
| Affiliation (Group) | 0.39 | 0.19 – 0.60 | **<0.001** | 0.40 | 0.19 – 0.60 | **<0.001** | 0.24 | 0.03 – 0.46 | **0.004** |
| Affiliation (Ind.) | 0.13 | -0.07 – 0.34 | 0.096 | -0.04 | -0.25 – 0.17 | 0.630 | 0.27 | 0.05 – 0.49 | **0.001** |
| Affiliation (Excl.) | -0.13 | -0.34 – 0.08 | 0.106 | -0.27 | -0.48 – -0.06 | **0.001** | -0.21 | -0.42 – 0.01 | **0.013** |
| Self-protection | 0.22 | 0.01 – 0.43 | **0.006** | 0.23 | 0.02 – 0.43 | **0.005** | 0.22 | 0.01 – 0.44 | **0.008** |
| Status | 0.51 | 0.30 – 0.72 | **<0.001** | 0.50 | 0.29 – 0.70 | **<0.001** | 0.36 | 0.14 – 0.58 | **<0.001** |
| Disease Avoidance | -0.21 | -0.42 – -0.00 | **0.009** | -0.40 | -0.61 – -0.20 | **<0.001** | -0.27 | -0.49 – -0.05 | **0.001** |
| Mate Seeking | -1.00 | -1.20 – -0.79 | **<0.001** | -0.93 | -1.14 – -0.72 | **<0.001** | -0.77 | -0.98 – -0.55 | **<0.001** |
| Observations | 1100 | | | 1056 | | | 1122 | | |
| R^2^ / R^2^ adjusted | 0.295 / 0.289 | | | 0.329 / 0.323 | | | 0.218 / 0.211 | | |

### Study 4: Dark Alley

|  | **Young Man** | | | **Young Woman** | | | **Elderly Man** | | | **Elderly Woman** | | |
| --- | --- | --- | --- | --- | --- | --- | --- | --- | --- | --- | --- | --- |
| *Predictors* | *std. Beta* | *standardized CI* | *p* | *std. Beta* | *standardized CI* | *p* | *std. Beta* | *standardized CI* | *p* | *std. Beta* | *standardized CI* | *p* |
| Intercept (Grand Mean) | 0.00 | -0.07 – 0.07 | **<0.001** | 0.00 | -0.07 – 0.07 | **<0.001** | -0.00 | -0.07 – 0.07 | **<0.001** | 0.00 | -0.07 – 0.07 | **<0.001** |
| Kin Care (Fam.) | 0.79 | 0.57 – 1.01 | **<0.001** | 0.67 | 0.45 – 0.89 | **<0.001** | 0.63 | 0.41 – 0.85 | **<0.001** | 0.70 | 0.48 – 0.91 | **<0.001** |
| Kin Care (Child.) | 0.68 | 0.46 – 0.90 | **<0.001** | 0.52 | 0.29 – 0.74 | **<0.001** | 0.54 | 0.32 – 0.75 | **<0.001** | 0.38 | 0.16 – 0.59 | **<0.001** |
| Mate Retention (Gen.) | -0.07 | -0.29 – 0.15 | 0.437 | 0.29 | 0.07 – 0.51 | **0.001** | 0.01 | -0.21 – 0.23 | 0.896 | -0.15 | -0.36 – 0.07 | 0.079 |
| Mate Retention (Breakup.) | -0.65 | -0.87 – -0.43 | **<0.001** | -0.53 | -0.75 – -0.31 | **<0.001** | -0.75 | -0.96 – -0.53 | **<0.001** | -0.85 | -1.07 – -0.63 | **<0.001** |
| Affiliation (Group) | 0.08 | -0.14 – 0.30 | 0.342 | -0.15 | -0.38 – 0.07 | 0.074 | 0.23 | 0.02 – 0.45 | **0.006** | 0.24 | 0.02 – 0.46 | **0.004** |
| Affiliation (Ind.) | -0.18 | -0.40 – 0.04 | **0.031** | 0.08 | -0.14 – 0.31 | 0.327 | 0.01 | -0.20 – 0.23 | 0.862 | 0.25 | 0.03 – 0.47 | **0.003** |
| Affiliation (Excl.) | -0.07 | -0.29 – 0.15 | 0.437 | -0.25 | -0.47 – -0.03 | **0.004** | 0.03 | -0.19 – 0.24 | 0.761 | 0.05 | -0.17 – 0.27 | 0.548 |
| Self-protection | 0.60 | 0.38 – 0.81 | **<0.001** | 0.55 | 0.32 – 0.77 | **<0.001** | 0.64 | 0.42 – 0.86 | **<0.001** | 0.53 | 0.31 – 0.75 | **<0.001** |
| Status | -0.43 | -0.65 – -0.21 | **<0.001** | -0.54 | -0.76 – -0.32 | **<0.001** | -0.52 | -0.74 – -0.30 | **<0.001** | -0.32 | -0.54 – -0.10 | **<0.001** |
| Disease Avoidance | 0.00 | -0.22 – 0.22 | 0.966 | -0.01 | -0.24 – 0.21 | 0.865 | -0.02 | -0.23 – 0.20 | 0.828 | -0.15 | -0.36 – 0.07 | 0.079 |
| Mate Seeking | -0.75 | -0.97 – -0.53 | **<0.001** | -0.62 | -0.84 – -0.40 | **<0.001** | -0.81 | -1.02 – -0.59 | **<0.001** | -0.67 | -0.89 – -0.46 | **<0.001** |
| Observations | 1056 | | | 1100 | | | 1089 | | | 1122 | | |
| R^2^ / R^2^ adjusted | 0.244 / 0.237 | | | 0.195 / 0.188 | | | 0.239 / 0.232 | | | 0.214 / 0.207 | | |

## Rating Scale Measure

### Study 1: Gender

|  | **No Context (All Studies)** | | | **Man** | | | **Woman** | | |
| --- | --- | --- | --- | --- | --- | --- | --- | --- | --- |
| *Predictors* | *std. Beta* | *standardized CI* | *p* | *std. Beta* | *standardized CI* | *p* | *std. Beta* | *standardized CI* | *p* |
| Intercept (Grand Mean) | -0.00 | -0.04 – 0.04 | **<0.001** | -0.00 | -0.07 – 0.07 | **<0.001** | -0.00 | -0.07 – 0.07 | **<0.001** |
| Kin Care (Fam.) | 0.52 | 0.40 – 0.63 | **<0.001** | 0.57 | 0.34 – 0.80 | **<0.001** | 0.44 | 0.21 – 0.67 | **<0.001** |
| Kin Care (Child.) | 0.37 | 0.25 – 0.48 | **<0.001** | 0.52 | 0.29 – 0.75 | **<0.001** | 0.29 | 0.06 – 0.53 | **0.001** |
| Mate Retention (Gen.) | 0.42 | 0.30 – 0.53 | **<0.001** | 0.57 | 0.34 – 0.80 | **<0.001** | 0.42 | 0.19 – 0.66 | **<0.001** |
| Mate Retention (Breakup.) | -0.41 | -0.53 – -0.30 | **<0.001** | -0.42 | -0.66 – -0.19 | **<0.001** | -0.38 | -0.62 – -0.15 | **<0.001** |
| Affiliation (Group) | -0.12 | -0.24 – -0.01 | **0.006** | -0.19 | -0.42 – 0.04 | **0.031** | -0.09 | -0.32 – 0.15 | 0.347 |
| Affiliation (Ind.) | 0.31 | 0.20 – 0.43 | **<0.001** | 0.17 | -0.06 – 0.40 | 0.064 | 0.50 | 0.26 – 0.73 | **<0.001** |
| Affiliation (Excl.) | -0.34 | -0.45 – -0.22 | **<0.001** | -0.42 | -0.66 – -0.19 | **<0.001** | -0.35 | -0.58 – -0.11 | **<0.001** |
| Self-protection | -0.04 | -0.15 – 0.08 | 0.398 | -0.16 | -0.39 – 0.07 | 0.072 | -0.03 | -0.27 – 0.20 | 0.700 |
| Status | -0.17 | -0.29 – -0.06 | **<0.001** | -0.15 | -0.39 – 0.08 | 0.083 | -0.02 | -0.26 – 0.21 | 0.805 |
| Disease Avoidance | -0.37 | -0.48 – -0.25 | **<0.001** | -0.33 | -0.56 – -0.10 | **<0.001** | -0.39 | -0.62 – -0.15 | **<0.001** |
| Mate Seeking | -0.16 | -0.28 – -0.05 | **<0.001** | -0.15 | -0.38 – 0.08 | 0.097 | -0.39 | -0.63 – -0.16 | **<0.001** |
| Observations | 4485 | | | 1088 | | | 1087 | | |
| R^2^ / R^2^ adjusted | 0.106 / 0.104 | | | 0.140 / 0.132 | | | 0.117 / 0.109 | | |

### Study 2: Dating

|  | **Casual Sexual Partner** | | | **Potential Life Partner** | | |
| --- | --- | --- | --- | --- | --- | --- |
| *Predictors* | *std. Beta* | *standardized CI* | *p* | *std. Beta* | *standardized CI* | *p* |
| Intercept (Grand Mean) | 0.00 | -0.07 – 0.07 | **<0.001** | -0.00 | -0.07 – 0.07 | **<0.001** |
| Kin Care (Fam.) | 0.00 | -0.23 – 0.23 | 0.981 | 0.64 | 0.42 – 0.86 | **<0.001** |
| Kin Care (Child.) | -0.16 | -0.39 – 0.07 | 0.077 | 0.46 | 0.24 – 0.68 | **<0.001** |
| Mate Retention (Gen.) | 0.45 | 0.22 – 0.68 | **<0.001** | 0.80 | 0.58 – 1.02 | **<0.001** |
| Mate Retention (Breakup.) | -0.25 | -0.48 – -0.01 | **0.006** | -0.40 | -0.61 – -0.18 | **<0.001** |
| Affiliation (Group) | -0.38 | -0.61 – -0.15 | **<0.001** | -0.42 | -0.63 – -0.20 | **<0.001** |
| Affiliation (Ind.) | 0.08 | -0.15 – 0.31 | 0.358 | 0.22 | -0.00 – 0.44 | **0.011** |
| Affiliation (Excl.) | -0.52 | -0.75 – -0.29 | **<0.001** | -0.48 | -0.70 – -0.26 | **<0.001** |
| Self-protection | 0.36 | 0.13 – 0.59 | **<0.001** | -0.04 | -0.25 – 0.18 | 0.674 |
| Status | -0.47 | -0.71 – -0.24 | **<0.001** | -0.39 | -0.61 – -0.17 | **<0.001** |
| Disease Avoidance | 0.64 | 0.41 – 0.87 | **<0.001** | -0.30 | -0.52 – -0.08 | **<0.001** |
| Mate Seeking | 0.24 | 0.01 – 0.48 | **0.007** | -0.10 | -0.32 – 0.12 | 0.244 |
| Observations | 1078 | | | 1133 | | |
| R^2^ / R^2^ adjusted | 0.139 / 0.131 | | | 0.193 / 0.185 | | |

### Study 3: Workplace

|  | **New Assistant** | | | **New Boss** | | | **New Coworker** | | |
| --- | --- | --- | --- | --- | --- | --- | --- | --- | --- |
| *Predictors* | *std. Beta* | *standardized CI* | *p* | *std. Beta* | *standardized CI* | *p* | *std. Beta* | *standardized CI* | *p* |
| Intercept (Grand Mean) | 0.00 | -0.07 – 0.07 | **<0.001** | 0.00 | -0.07 – 0.07 | **<0.001** | -0.00 | -0.07 – 0.07 | **<0.001** |
| Kin Care (Fam.) | 0.39 | 0.17 – 0.61 | **<0.001** | 0.61 | 0.38 – 0.83 | **<0.001** | 0.37 | 0.13 – 0.60 | **<0.001** |
| Kin Care (Child.) | 0.29 | 0.07 – 0.51 | **0.001** | 0.50 | 0.28 – 0.73 | **<0.001** | 0.27 | 0.04 – 0.50 | **0.003** |
| Mate Retention (Gen.) | -0.21 | -0.43 – 0.01 | **0.012** | -0.24 | -0.46 – -0.01 | **0.006** | -0.19 | -0.43 – 0.04 | **0.031** |
| Mate Retention (Breakup.) | -0.85 | -1.07 – -0.63 | **<0.001** | -0.69 | -0.92 – -0.46 | **<0.001** | -0.60 | -0.83 – -0.37 | **<0.001** |
| Affiliation (Group) | 0.35 | 0.13 – 0.57 | **<0.001** | 0.21 | -0.02 – 0.43 | **0.018** | 0.15 | -0.08 – 0.38 | 0.098 |
| Affiliation (Ind.) | 0.33 | 0.11 – 0.55 | **<0.001** | 0.12 | -0.11 – 0.34 | 0.173 | 0.32 | 0.09 – 0.55 | **<0.001** |
| Affiliation (Excl.) | 0.16 | -0.06 – 0.38 | 0.057 | 0.00 | -0.22 – 0.23 | 0.968 | -0.09 | -0.32 – 0.14 | 0.315 |
| Self-protection | 0.19 | -0.03 – 0.41 | **0.026** | 0.21 | -0.01 – 0.44 | **0.015** | 0.21 | -0.02 – 0.45 | **0.018** |
| Status | 0.51 | 0.29 – 0.73 | **<0.001** | 0.45 | 0.22 – 0.67 | **<0.001** | 0.30 | 0.06 – 0.53 | **0.001** |
| Disease Avoidance | -0.30 | -0.52 – -0.08 | **<0.001** | -0.38 | -0.61 – -0.16 | **<0.001** | -0.19 | -0.43 – 0.04 | **0.031** |
| Mate Seeking | -0.85 | -1.07 – -0.63 | **<0.001** | -0.79 | -1.02 – -0.57 | **<0.001** | -0.54 | -0.77 – -0.30 | **<0.001** |
| Observations | 1100 | | | 1054 | | | 1111 | | |
| R^2^ / R^2^ adjusted | 0.215 / 0.208 | | | 0.204 / 0.196 | | | 0.109 / 0.101 | | |

### Study 4: Dark Alley

|  | **Young Man** | | | **Young Woman** | | | **Elderly Man** | | | **Elderly Woman** | | |
| --- | --- | --- | --- | --- | --- | --- | --- | --- | --- | --- | --- | --- |
| *Predictors* | *std. Beta* | *standardized CI* | *p* | *std. Beta* | *standardized CI* | *p* | *std. Beta* | *standardized CI* | *p* | *std. Beta* | *standardized CI* | *p* |
| Intercept (Grand Mean) | -0.00 | -0.07 – 0.07 | **<0.001** | 0.00 | -0.08 – 0.08 | **<0.001** | -0.00 | -0.07 – 0.07 | **<0.001** | -0.00 | -0.07 – 0.07 | **<0.001** |
| Kin Care (Fam.) | 0.48 | 0.25 – 0.72 | **<0.001** | 0.25 | 0.01 – 0.49 | **0.008** | 0.31 | 0.08 – 0.54 | **0.001** | 0.35 | 0.12 – 0.58 | **<0.001** |
| Kin Care (Child.) | 0.59 | 0.36 – 0.83 | **<0.001** | 0.31 | 0.07 – 0.55 | **0.001** | 0.44 | 0.21 – 0.68 | **<0.001** | 0.30 | 0.07 – 0.54 | **0.001** |
| Mate Retention (Gen.) | -0.19 | -0.42 – 0.05 | **0.042** | 0.10 | -0.14 – 0.34 | 0.270 | -0.21 | -0.45 – 0.02 | **0.019** | -0.10 | -0.33 – 0.14 | 0.290 |
| Mate Retention (Breakup.) | -0.46 | -0.70 – -0.22 | **<0.001** | -0.27 | -0.51 – -0.03 | **0.003** | -0.44 | -0.68 – -0.21 | **<0.001** | -0.43 | -0.67 – -0.20 | **<0.001** |
| Affiliation (Group) | 0.03 | -0.20 – 0.27 | 0.721 | -0.14 | -0.38 – 0.10 | 0.131 | 0.09 | -0.15 – 0.32 | 0.335 | 0.14 | -0.09 – 0.37 | 0.125 |
| Affiliation (Ind.) | 0.04 | -0.20 – 0.28 | 0.674 | 0.18 | -0.06 – 0.42 | 0.056 | 0.08 | -0.16 – 0.31 | 0.399 | 0.26 | 0.03 – 0.49 | **0.004** |
| Affiliation (Excl.) | -0.02 | -0.25 – 0.22 | 0.848 | -0.09 | -0.33 – 0.15 | 0.352 | 0.07 | -0.17 – 0.30 | 0.469 | 0.08 | -0.15 – 0.31 | 0.376 |
| Self-protection | 0.45 | 0.22 – 0.69 | **<0.001** | 0.42 | 0.18 – 0.66 | **<0.001** | 0.52 | 0.29 – 0.76 | **<0.001** | 0.40 | 0.17 – 0.64 | **<0.001** |
| Status | -0.41 | -0.64 – -0.17 | **<0.001** | -0.39 | -0.63 – -0.15 | **<0.001** | -0.39 | -0.63 – -0.16 | **<0.001** | -0.31 | -0.54 – -0.08 | **0.001** |
| Disease Avoidance | -0.08 | -0.31 – 0.16 | 0.409 | -0.07 | -0.31 – 0.17 | 0.450 | 0.15 | -0.09 – 0.38 | 0.104 | -0.32 | -0.55 – -0.09 | **<0.001** |
| Mate Seeking | -0.45 | -0.69 – -0.22 | **<0.001** | -0.29 | -0.53 – -0.05 | **0.002** | -0.61 | -0.84 – -0.37 | **<0.001** | -0.38 | -0.62 – -0.15 | **<0.001** |
| Observations | 1053 | | | 1098 | | | 1076 | | | 1121 | | |
| R^2^ / R^2^ adjusted | 0.128 / 0.120 | | | 0.065 / 0.057 | | | 0.125 / 0.116 | | | 0.092 / 0.084 | | |

# Motive Stability

## Mean Motive Stability Ratings

| **Motive** |  | *99% CI* | |  |
| --- | --- | --- | --- | --- |
|  | **Mean** | **Lower** | **Upper** | **SD** |
| Kin Care (Child) | 5.822 | 5.732 | 5.909 | 1.317 |
| Kin Care (Fam.) | 5.603 | 5.518 | 5.684 | 1.249 |
| Mate Retention (Gen.) | 5.266 | 5.171 | 5.362 | 1.441 |
| Affiliation (Indep.) | 5.156 | 5.066 | 5.243 | 1.303 |
| Self-protection | 4.939 | 4.845 | 5.034 | 1.408 |
| Affiliation (Group) | 4.775 | 4.684 | 4.862 | 1.335 |
| Status | 4.764 | 4.673 | 4.857 | 1.393 |
| Disease Avoidance | 4.682 | 4.583 | 4.781 | 1.520 |
| Affiliation (Exclu. Conc.) | 4.457 | 4.363 | 4.553 | 1.423 |
| Mate Retention (Breakup Conc.) | 4.214 | 4.105 | 4.320 | 1.559 |
| Mate Seeking | 3.829 | 3.726 | 3.929 | 1.525 |

# Motive Stability Correlations with Motive Prioritization

## 7 Key Fundamental Social Motives

|  | *Stability Rating* | *Prioritization FC NC* | *Prioritization FC C* | *Prioritization RS NC* | *Prioritization RS C* |
| --- | --- | --- | --- | --- | --- |
| *Stability Rating* |  |  |  |  |  |
| *Prioritization FC NC* | 0.858 *(.014)* |  |  |  |  |
| *Prioritization FC C* | 0.969 *(<.001)* | 0.856 *(.014)* |  |  |  |
| *Prioritization RS NC* | 0.746 *(.054)* | 0.967 *(<.001)* | 0.766 *(.045)* |  |  |
| *Prioritization RS C* | 0.920 *(.003)* | 0.787 *(.036)* | 0.984 *(<.001)* | 0.717 *(.070)* |  |
| *Computed correlation used pearson-method with pairwise-deletion.* | | | | | |

*Note: FC = Forced-Choice Measure; RS = Rating Scale Measure; NC = No Context Condition; C = Collapsed Context Conditions*

## All 11 Fundamental Social Motive Subscales

|  | *Stability Rating* | *Prioritization FC NC* | *Prioritization FC C* | *Prioritization RS NC* | *Prioritization RS C* |
| --- | --- | --- | --- | --- | --- |
| *Stability Rating* |  |  |  |  |  |
| *Prioritization FC NC* | 0.889 *(<.001)* |  |  |  |  |
| *Prioritization FC C* | 0.934 *(<.001)* | 0.914 *(<.001)* |  |  |  |
| *Prioritization RS NC* | 0.830 *(.002)* | 0.962 *(<.001)* | 0.825 *(.002)* |  |  |
| *Prioritization RS C* | 0.927 *(<.001)* | 0.869 *(.001)* | 0.969 *(<.001)* | 0.825 *(.002)* |  |
| *Computed correlation used pearson-method with pairwise-deletion.* | | | | | |

*Note: FC = Forced-Choice Measure; RS = Rating Scale Measure; NC = No Context Condition; C = Collapsed Context Conditions*

# Study 5: Trait Inference

## Mean Motive Prioritization Ratings: Rating Scale Measure

| **Motive** | **N** | **Mean** | **SD** |
| --- | --- | --- | --- |
| Kin Care (Fam.) | 174 | 5.379310 | 1.327707 |
| Self-protection | 174 | 4.965517 | 1.454101 |
| Disease Avoidance | 174 | 4.810345 | 1.599750 |
| Status | 174 | 4.954023 | 1.453782 |
| Affiliation (Group) | 174 | 5.195402 | 1.469057 |
| Mate Seeking | 174 | 4.678161 | 1.743654 |
| Mate Retention (Gen.) | 174 | 5.419540 | 1.443289 |

## Mean Trait Inference Ratings

| **Motive** | **N** | **Mean** | **SD** |
| --- | --- | --- | --- |
| Kin Care Warmth | 174 | 5.655 | 1.252 |
| Kin Care Conscientiousness | 174 | 5.443 | 1.328 |
| Kin Care Competence | 174 | 5.385 | 1.417 |
| Kin Care Honesty | 174 | 5.408 | 1.321 |
| Kin Care Neuroticism | 174 | 3.868 | 1.796 |
| Kin Care Openness | 174 | 4.368 | 1.847 |
| Kin Care Extraversion | 174 | 4.764 | 1.798 |
| Mate Retention Warmth | 174 | 5.603 | 1.216 |
| Mate Retention Conscientiousness | 174 | 5.264 | 1.569 |
| Mate Retention Competence | 174 | 5.017 | 1.589 |
| Mate Retention Honesty | 174 | 5.575 | 1.448 |
| Mate Retention Neuroticism | 174 | 3.833 | 1.893 |
| Mate Retention Openness | 174 | 4.563 | 1.732 |
| Mate Retention Extraversion | 174 | 4.678 | 1.724 |
| Affiliation Warmth | 174 | 5.236 | 1.496 |
| Affiliation Conscientiousness | 174 | 4.730 | 1.857 |
| Affiliation Competence | 174 | 4.626 | 1.838 |
| Affiliation Honesty | 174 | 4.833 | 1.656 |
| Affiliation Neuroticism | 174 | 4.190 | 1.857 |
| Affiliation Openness | 174 | 5.305 | 1.366 |
| Affiliation Extraversion | 174 | 5.552 | 1.301 |
| Self-Protection Warmth | 174 | 4.580 | 1.787 |
| Self-Protection Conscientiousness | 174 | 4.810 | 1.774 |
| Self-Protection Competence | 174 | 4.701 | 1.835 |
| Self-Protection Honesty | 174 | 4.586 | 1.822 |
| Self-Protection Neuroticism | 174 | 4.954 | 1.726 |
| Self-Protection Openness | 174 | 4.477 | 1.868 |
| Self-Protection Extraversion | 174 | 4.523 | 1.833 |
| Disease Avoidance Warmth | 174 | 4.356 | 1.850 |
| Disease Avoidance Conscientiousness | 174 | 4.759 | 1.676 |
| Disease Avoidance Competence | 174 | 4.471 | 1.855 |
| Disease Avoidance Honesty | 174 | 4.557 | 1.768 |
| Disease Avoidance Neuroticism | 174 | 5.063 | 1.566 |
| Disease Avoidance Openness | 174 | 4.172 | 1.903 |
| Disease Avoidance Extraversion | 174 | 4.126 | 1.867 |
| Status Warmth | 174 | 4.701 | 1.764 |
| Status Conscientiousness | 174 | 5.454 | 1.324 |
| Status Competence | 174 | 5.494 | 1.333 |
| Status Honesty | 174 | 4.793 | 1.646 |
| Status Neuroticism | 174 | 3.764 | 1.846 |
| Status Openness | 174 | 5.075 | 1.566 |
| Status Extraversion | 174 | 5.299 | 1.427 |
| Mate Seeking Warmth | 174 | 4.649 | 1.743 |
| Mate Seeking Conscientiousness | 174 | 4.213 | 1.839 |
| Mate Seeking Competence | 174 | 4.362 | 1.884 |
| Mate Seeking Honesty | 174 | 4.351 | 1.950 |
| Mate Seeking Neuroticism | 174 | 3.724 | 1.882 |
| Mate Seeking Openness | 174 | 5.310 | 1.527 |
| Mate Seeking Extraversion | 174 | 5.443 | 1.408 |

## Correlations Among Motive Prioritization Scores

|  | *1* | *2* | *3* | *4* | *5* | *6* | *7* |
| --- | --- | --- | --- | --- | --- | --- | --- |
| *1. Kin Care (Fam)* |  |  |  |  |  |  |  |
| *2. Self-Protection* | 0.294 *(<.001)* |  |  |  |  |  |  |
| *3. Disease Avoidance* | 0.143 *(.060)* | 0.318 *(<.001)* |  |  |  |  |  |
| *4. Status* | 0.192 *(.011)* | 0.363 *(<.001)* | 0.327 *(<.001)* |  |  |  |  |
| *5. Affiliation (Group)* | 0.089 *(.242)* | 0.417 *(<.001)* | 0.220 *(.004)* | 0.510 *(<.001)* |  |  |  |
| *6. Mate Seeking* | 0.036 *(.641)* | 0.167 *(.028)* | 0.142 *(.062)* | 0.370 *(<.001)* | 0.334 *(<.001)* |  |  |
| *7. Mate Retention (General)* | 0.420 *(<.001)* | 0.310 *(<.001)* | 0.205 *(.007)* | 0.329 *(<.001)* | 0.299 *(<.001)* | 0.343 *(<.001)* |  |

## Trait Inference Correlations with Motive Prioritization Scores

### Motive as Unit of Analysis

|  | *1* | *2* | *3* | *4* | *5* | *6* | *7* | *8* | *9* | *10* | *11* |
| --- | --- | --- | --- | --- | --- | --- | --- | --- | --- | --- | --- |
| *1 Prioritization FC NC* |  |  |  |  |  |  |  |  |  |  |  |
| *2 Prioritization FC C* | 0.856 *(.014)* |  |  |  |  |  |  |  |  |  |  |
| *3 Prioritization RS NC* | 0.967 *(<.001)* | 0.766 *(.045)* |  |  |  |  |  |  |  |  |  |
| *4 Prioritization RS C* | 0.787 *(.036)* | 0.984 *(<.001)* | 0.717 *(.070)* |  |  |  |  |  |  |  |  |
| *5 Warmth* | 0.929 *(.002)* | 0.670 *(.100)* | 0.895 *(.006)* | 0.580 *(.172)* |  |  |  |  |  |  |  |
| *6 Conscientiousness* | 0.655 *(.110)* | 0.698 *(.081)* | 0.581 *(.171)* | 0.640 *(.122)* | 0.525 *(.226)* |  |  |  |  |  |  |
| *7 Competence* | 0.569 *(.182)* | 0.560 *(.191)* | 0.555 *(.196)* | 0.530 *(.221)* | 0.493 *(.261)* | 0.943 *(.001)* |  |  |  |  |  |
| *8 Honesty* | 0.951 *(.001)* | 0.765 *(.045)* | 0.892 *(.007)* | 0.658 *(.108)* | 0.906 *(.005)* | 0.755 *(.050)* | 0.643 *(.120)* |  |  |  |  |
| *9 Neuroticism* | -0.348 *(.445)* | 0.086 *(.854)* | -0.456 *(.304)* | 0.145 *(.756)* | -0.564 *(.187)* | -0.265 *(.565)* | -0.474 *(.282)* | -0.409 *(.363)* |  |  |  |
| *10 Openness* | -0.360 *(.428)* | -0.588 *(.165)* | -0.292 *(.525)* | -0.569 *(.182)* | -0.026 *(.955)* | -0.367 *(.418)* | -0.132 *(.778)* | -0.325 *(.477)* | -0.572 *(.179)* |  |  |
| *11 Extraversion* | -0.161 *(.730)* | -0.402 *(.372)* | -0.099 *(.832)* | -0.391 *(.386)* | 0.168 *(.718)* | -0.192 *(.679)* | 0.060 *(.898)* | -0.150 *(.748)* | -0.693 *(.084)* | 0.965 *(<.001)* |  |
| *Computed correlation used pearson-method with pairwise-deletion.* | | | | | | | | | | | |

*Note: FC = Forced-Choice Measure; RS = Rating Scale Measure; NC = No Context Condition; C = Collapsed Context Conditions*

### Individual as Unit of Analysis

#### Trait Inference (Kin Care)

|  | *Kin Care* | *Self-Protection* | *Disease Avoidance* | *Status* | *Affiliation* | *Mate Seeking* | *Mate Retention* |
| --- | --- | --- | --- | --- | --- | --- | --- |
| *Warmth* | 0.218 *(.004)* | 0.244 *(.001)* | 0.010 *(.891)* | 0.137 *(.071)* | 0.291 *(<.001)* | 0.110 *(.147)* | 0.205 *(.007)* |
| *Conscientiousness* | 0.190 *(.012)* | 0.283 *(<.001)* | 0.249 *(.001)* | 0.256 *(.001)* | 0.210 *(.005)* | 0.067 *(.381)* | 0.183 *(.016)* |
| *Competence* | 0.208 *(.006)* | 0.287 *(<.001)* | 0.372 *(<.001)* | 0.188 *(.013)* | 0.216 *(.004)* | 0.100 *(.191)* | 0.209 *(.006)* |
| *Honesty* | 0.195 *(.010)* | 0.242 *(.001)* | 0.239 *(.001)* | 0.139 *(.067)* | 0.200 *(.008)* | 0.082 *(.280)* | 0.086 *(.262)* |
| *Neuroticism* | 0.084 *(.269)* | -0.084 *(.272)* | 0.194 *(.010)* | 0.235 *(.002)* | 0.034 *(.657)* | 0.243 *(.001)* | 0.102 *(.181)* |
| *Openness* | 0.150 *(.048)* | 0.102 *(.182)* | 0.403 *(<.001)* | 0.269 *(<.001)* | 0.171 *(.024)* | 0.211 *(.005)* | 0.107 *(.162)* |
| *Extraversion* | 0.231 *(.002)* | 0.200 *(.008)* | 0.445 *(<.001)* | 0.290 *(<.001)* | 0.193 *(.011)* | 0.101 *(.185)* | 0.210 *(.005)* |

#### Trait Inference (Mate Retention)

|  | *Kin Care* | *Self-Protection* | *Disease Avoidance* | *Status* | *Affiliation* | *Mate Seeking* | *Mate Retention* |
| --- | --- | --- | --- | --- | --- | --- | --- |
| *Warmth* | 0.276 *(<.001)* | 0.231 *(.002)* | 0.223 *(.003)* | 0.143 *(.059)* | 0.167 *(.028)* | 0.032 *(.674)* | 0.184 *(.015)* |
| *Conscientiousness* | 0.210 *(.006)* | 0.260 *(.001)* | 0.366 *(<.001)* | 0.198 *(.009)* | 0.253 *(.001)* | 0.099 *(.194)* | 0.163 *(.032)* |
| *Competence* | 0.202 *(.007)* | 0.308 *(<.001)* | 0.388 *(<.001)* | 0.283 *(<.001)* | 0.308 *(<.001)* | 0.067 *(.382)* | 0.171 *(.024)* |
| *Honesty* | 0.145 *(.057)* | 0.295 *(<.001)* | 0.195 *(.010)* | 0.183 *(.016)* | 0.276 *(<.001)* | 0.154 *(.043)* | 0.130 *(.087)* |
| *Neuroticism* | 0.039 *(.609)* | 0.031 *(.680)* | 0.272 *(<.001)* | 0.230 *(.002)* | 0.020 *(.792)* | 0.274 *(<.001)* | 0.125 *(.100)* |
| *Openness* | 0.153 *(.044)* | 0.038 *(.622)* | 0.325 *(<.001)* | 0.222 *(.003)* | 0.129 *(.089)* | 0.137 *(.072)* | 0.143 *(.060)* |
| *Extraversion* | 0.246 *(.001)* | 0.268 *(<.001)* | 0.491 *(<.001)* | 0.303 *(<.001)* | 0.290 *(<.001)* | 0.138 *(.068)* | 0.282 *(<.001)* |

#### Trait Inference (Affiliation)

|  | *Kin Care* | *Self-Protection* | *Disease Avoidance* | *Status* | *Affiliation* | *Mate Seeking* | *Mate Retention* |
| --- | --- | --- | --- | --- | --- | --- | --- |
| *Warmth* | 0.220 *(.004)* | 0.160 *(.034)* | 0.318 *(<.001)* | 0.231 *(.002)* | 0.344 *(<.001)* | 0.080 *(.293)* | 0.179 *(.018)* |
| *Conscientiousness* | 0.213 *(.005)* | 0.361 *(<.001)* | 0.430 *(<.001)* | 0.314 *(<.001)* | 0.380 *(<.001)* | 0.150 *(.049)* | 0.191 *(.011)* |
| *Competence* | 0.167 *(.027)* | 0.300 *(<.001)* | 0.416 *(<.001)* | 0.270 *(<.001)* | 0.353 *(<.001)* | 0.125 *(.101)* | 0.171 *(.024)* |
| *Honesty* | 0.097 *(.202)* | 0.156 *(.040)* | 0.442 *(<.001)* | 0.191 *(.011)* | 0.254 *(.001)* | 0.115 *(.129)* | 0.015 *(.845)* |
| *Neuroticism* | -0.036 *(.634)* | 0.097 *(.205)* | 0.222 *(.003)* | 0.260 *(.001)* | 0.069 *(.366)* | 0.253 *(.001)* | 0.216 *(.004)* |
| *Openness* | 0.207 *(.006)* | 0.235 *(.002)* | 0.349 *(<.001)* | 0.321 *(<.001)* | 0.362 *(<.001)* | 0.073 *(.339)* | 0.210 *(.005)* |
| *Extraversion* | 0.209 *(.006)* | 0.038 *(.622)* | 0.225 *(.003)* | 0.185 *(.015)* | 0.146 *(.055)* | 0.048 *(.528)* | 0.258 *(.001)* |

#### Trait Inference (Self-Protection)

|  | *Kin Care* | *Self-Protection* | *Disease Avoidance* | *Status* | *Affiliation* | *Mate Seeking* | *Mate Retention* |
| --- | --- | --- | --- | --- | --- | --- | --- |
| *Warmth* | 0.253 *(.001)* | 0.208 *(.006)* | 0.310 *(<.001)* | 0.268 *(<.001)* | 0.225 *(.003)* | 0.123 *(.105)* | 0.125 *(.101)* |
| *Conscientiousness* | 0.207 *(.006)* | 0.311 *(<.001)* | 0.382 *(<.001)* | 0.306 *(<.001)* | 0.249 *(.001)* | 0.060 *(.428)* | 0.230 *(.002)* |
| *Competence* | 0.239 *(.001)* | 0.338 *(<.001)* | 0.372 *(<.001)* | 0.417 *(<.001)* | 0.419 *(<.001)* | 0.163 *(.032)* | 0.174 *(.022)* |
| *Honesty* | 0.185 *(.015)* | 0.307 *(<.001)* | 0.342 *(<.001)* | 0.331 *(<.001)* | 0.261 *(<.001)* | 0.114 *(.133)* | 0.143 *(.059)* |
| *Neuroticism* | 0.043 *(.574)* | 0.025 *(.746)* | 0.049 *(.520)* | -0.012 *(.871)* | -0.019 *(.801)* | 0.195 *(.010)* | 0.105 *(.167)* |
| *Openness* | 0.092 *(.227)* | 0.132 *(.083)* | 0.268 *(<.001)* | 0.144 *(.057)* | 0.160 *(.035)* | 0.291 *(<.001)* | 0.140 *(.066)* |
| *Extraversion* | 0.113 *(.138)* | -0.002 *(.980)* | 0.284 *(<.001)* | 0.196 *(.010)* | 0.176 *(.020)* | 0.299 *(<.001)* | 0.185 *(.014)* |

#### Trait Inference (Disease Avoidance)

|  | *Kin Care* | *Self-Protection* | *Disease Avoidance* | *Status* | *Affiliation* | *Mate Seeking* | *Mate Retention* |
| --- | --- | --- | --- | --- | --- | --- | --- |
| *Warmth* | 0.140 *(.065)* | 0.189 *(.012)* | 0.253 *(.001)* | 0.116 *(.128)* | 0.164 *(.031)* | 0.274 *(<.001)* | 0.100 *(.191)* |
| *Conscientiousness* | 0.145 *(.056)* | 0.250 *(.001)* | 0.382 *(<.001)* | 0.287 *(<.001)* | 0.193 *(.011)* | 0.163 *(.031)* | 0.147 *(.052)* |
| *Competence* | 0.110 *(.148)* | 0.237 *(.002)* | 0.496 *(<.001)* | 0.295 *(<.001)* | 0.223 *(.003)* | 0.169 *(.026)* | 0.150 *(.048)* |
| *Honesty* | 0.133 *(.079)* | 0.199 *(.009)* | 0.444 *(<.001)* | 0.224 *(.003)* | 0.160 *(.035)* | 0.109 *(.152)* | 0.048 *(.527)* |
| *Neuroticism* | 0.091 *(.231)* | 0.120 *(.114)* | 0.037 *(.627)* | 0.017 *(.829)* | 0.103 *(.178)* | 0.115 *(.129)* | 0.172 *(.023)* |
| *Openness* | 0.113 *(.136)* | 0.104 *(.170)* | 0.370 *(<.001)* | 0.287 *(<.001)* | 0.234 *(.002)* | 0.341 *(<.001)* | 0.070 *(.357)* |
| *Extraversion* | 0.081 *(.289)* | 0.097 *(.201)* | 0.368 *(<.001)* | 0.172 *(.023)* | 0.130 *(.087)* | 0.220 *(.003)* | 0.060 *(.435)* |

#### Trait Inference (Status)

|  | *Kin Care* | *Self-Protection* | *Disease Avoidance* | *Status* | *Affiliation* | *Mate Seeking* | *Mate Retention* |
| --- | --- | --- | --- | --- | --- | --- | --- |
| *Warmth* | 0.288 *(<.001)* | 0.190 *(.012)* | 0.236 *(.002)* | 0.263 *(<.001)* | 0.351 *(<.001)* | 0.264 *(<.001)* | 0.222 *(.003)* |
| *Conscientiousness* | 0.105 *(.166)* | 0.155 *(.041)* | 0.221 *(.003)* | 0.239 *(.001)* | 0.147 *(.052)* | 0.084 *(.272)* | 0.142 *(.062)* |
| *Competence* | -0.028 *(.712)* | 0.247 *(.001)* | 0.318 *(<.001)* | 0.244 *(.001)* | 0.372 *(<.001)* | 0.131 *(.085)* | 0.162 *(.033)* |
| *Honesty* | 0.295 *(<.001)* | 0.241 *(.001)* | 0.341 *(<.001)* | 0.320 *(<.001)* | 0.304 *(<.001)* | 0.124 *(.104)* | 0.241 *(.001)* |
| *Neuroticism* | -0.032 *(.678)* | 0.027 *(.723)* | 0.261 *(.001)* | 0.257 *(.001)* | 0.009 *(.911)* | 0.262 *(<.001)* | 0.092 *(.229)* |
| *Openness* | 0.234 *(.002)* | 0.341 *(<.001)* | 0.354 *(<.001)* | 0.156 *(.039)* | 0.230 *(.002)* | -0.031 *(.681)* | 0.244 *(.001)* |
| *Extraversion* | 0.309 *(<.001)* | 0.264 *(<.001)* | 0.278 *(<.001)* | 0.193 *(.011)* | 0.151 *(.046)* | 0.099 *(.193)* | 0.284 *(<.001)* |

#### Trait Inference (Mate Seeking)

|  | *Kin Care* | *Self-Protection* | *Disease Avoidance* | *Status* | *Affiliation* | *Mate Seeking* | *Mate Retention* |
| --- | --- | --- | --- | --- | --- | --- | --- |
| *Warmth* | 0.273 *(<.001)* | 0.162 *(.033)* | 0.293 *(<.001)* | 0.270 *(<.001)* | 0.244 *(.001)* | 0.242 *(.001)* | 0.155 *(.041)* |
| *Conscientiousness* | 0.125 *(.099)* | 0.184 *(.015)* | 0.312 *(<.001)* | 0.337 *(<.001)* | 0.310 *(<.001)* | 0.398 *(<.001)* | 0.193 *(.011)* |
| *Competence* | 0.127 *(.094)* | 0.209 *(.006)* | 0.422 *(<.001)* | 0.380 *(<.001)* | 0.315 *(<.001)* | 0.287 *(<.001)* | 0.193 *(.011)* |
| *Honesty* | 0.145 *(.057)* | 0.206 *(.006)* | 0.385 *(<.001)* | 0.356 *(<.001)* | 0.265 *(<.001)* | 0.253 *(.001)* | 0.212 *(.005)* |
| *Neuroticism* | -0.046 *(.549)* | 0.121 *(.111)* | 0.290 *(<.001)* | 0.196 *(.010)* | 0.041 *(.596)* | 0.246 *(.001)* | 0.032 *(.673)* |
| *Openness* | 0.133 *(.081)* | 0.218 *(.004)* | 0.275 *(<.001)* | 0.189 *(.013)* | 0.341 *(<.001)* | 0.207 *(.006)* | 0.206 *(.007)* |
| *Extraversion* | 0.305 *(<.001)* | 0.219 *(.004)* | 0.235 *(.002)* | 0.219 *(.004)* | 0.254 *(.001)* | 0.138 *(.069)* | 0.358 *(<.001)* |

# Trait Inference Pilot (Unreported in Manuscript)

## Methods and Procedure

Participants were 303 North Americans recruited via the same procedures used in Studies 1 – 4. (*M*_age_ = 37.95, 62% female).

These participants were presented (on Qualtrics) with a set of 12 scenarios that had the following structure: “All you know about a person is this: The person is [very high / very low] in a motivation to [brief description of motive].” The brief descriptions of motives were identical to the descriptions used in constructing the motive prioritization measures employed in Studies 1 – 4. Each participant was presented with scenarios corresponding to a randomly-determined subset of 6 of 11 motive descriptions employed in Studies 1 – 4, corresponding to each of the 11 subscales of the FMSI questionnaire. (Thus, across all 303 participants, similar numbers of participants [n’s ranged from 159 to 168] were presented with scenarios containing each of the 11 motive descriptions.) For each motive description, participants were presented with 2 scenarios, one that described a person who was “very high” in that motivation, and the other that described a person who was “very low” in that motivation. Following each scenario, participants were instructed, “Now, based on this piece of information, rate how you think this person’s personality traits compare to those of the average person.” Participants rated the target person on 6 personality trait descriptions: *extroverted and enthusiastic*; *dependable and self-disciplined*; *complex and open to new experiences*; *anxious and easily upset*; *warm and sympathetic*; *capable and competent*. Ratings were made on 7-point scales (with endpoints labeled “far below average” and “far above average”).

If information about a specific motive is perceived to be more diagnostic of a specific trait, then one would expect a larger difference between trait ratings for an individual described as “very high” versus “very low” on that motive. We calculated *trait diagnosticity* scores accordingly: For each motive description, trait ratings for “very low” target persons were subtracted from corresponding trait ratings for “very high” target persons, and the absolute values of these differences were then computed.

## Mean Trait Inference Ratings

| **Motive** | **N** | **Mean** | **SD** |
| --- | --- | --- | --- |
| Kin Care Extraversion | 303 | 2.024 | 1.795 |
| Kin Care Conscientiousness | 303 | 2.723 | 1.798 |
| Kin Care Openness | 303 | 1.711 | 1.699 |
| Kin Care Neuroticism | 303 | 1.837 | 1.466 |
| Kin Care Warmth | 303 | 3.018 | 1.856 |
| Kin Care Competence | 303 | 2.018 | 1.700 |
| Child Care Extraversion | 303 | 2.169 | 1.767 |
| Child Care Conscientiousness | 303 | 2.503 | 1.745 |
| Child Care Openness | 303 | 2.042 | 1.694 |
| Child Care Neuroticism | 303 | 2.323 | 1.676 |
| Child Care Warmth | 303 | 3.199 | 1.696 |
| Child Care Competence | 303 | 2.084 | 1.686 |
| Breakup Concern Extraversion | 303 | 1.927 | 1.647 |
| Breakup Concern Conscientiousness | 303 | 1.739 | 1.569 |
| Breakup Concern Openness | 303 | 2.073 | 1.580 |
| Breakup Concern Neuroticism | 303 | 2.464 | 1.834 |
| Breakup Concern Warmth | 303 | 1.400 | 1.509 |
| Breakup Concern Competence | 303 | 1.178 | 1.490 |
| Mate Retention Extraversion | 303 | 2.079 | 1.803 |
| Mate Retention Conscientiousness | 303 | 2.756 | 1.883 |
| Mate Retention Openness | 303 | 2.196 | 1.849 |
| Mate Retention Neuroticism | 303 | 1.877 | 1.582 |
| Mate Retention Warmth | 303 | 2.454 | 1.740 |
| Mate Retention Competence | 303 | 1.821 | 1.664 |
| Exclusion Concern Extraversion | 303 | 2.975 | 1.895 |
| Exclusion Concern Conscientiousness | 303 | 1.883 | 1.683 |
| Exclusion Concern Openness | 303 | 2.402 | 1.792 |
| Exclusion Concern Neuroticism | 303 | 2.579 | 1.827 |
| Exclusion Concern Warmth | 303 | 1.724 | 1.607 |
| Exclusion Concern Competence | 303 | 1.484 | 1.601 |
| Affiliation Independence Extraversion | 303 | 3.617 | 1.891 |
| Affiliation Independence Conscientiousness | 303 | 2.120 | 1.576 |
| Affiliation Independence Openness | 303 | 2.317 | 1.669 |
| Affiliation Independence Neuroticism | 303 | 1.440 | 1.218 |
| Affiliation Independence Warmth | 303 | 1.629 | 1.408 |
| Affiliation Independence Competence | 303 | 1.413 | 1.436 |
| Status Extraversion | 303 | 2.939 | 1.815 |
| Status Conscientiousness | 303 | 2.601 | 1.720 |
| Status Openness | 303 | 2.350 | 1.804 |
| Status Neuroticism | 303 | 2.025 | 1.668 |
| Status Warmth | 303 | 1.669 | 1.647 |
| Status Competence | 303 | 2.141 | 1.699 |
| Self Protection Extraversion | 303 | 2.802 | 1.891 |
| Self Protection Conscientiousness | 303 | 2.198 | 1.664 |
| Self Protection Openness | 303 | 3.311 | 1.911 |
| Self Protection Neuroticism | 303 | 2.425 | 1.810 |
| Self Protection Warmth | 303 | 1.163 | 1.331 |
| Self Protection Competence | 303 | 1.222 | 1.479 |
| Affiliation Extraversion | 303 | 3.807 | 1.938 |
| Affiliation Conscientiousness | 303 | 1.702 | 1.588 |
| Affiliation Openness | 303 | 2.688 | 1.795 |
| Affiliation Neuroticism | 303 | 2.019 | 1.712 |
| Affiliation Warmth | 303 | 1.906 | 1.622 |
| Affiliation Competence | 303 | 1.516 | 1.621 |
| Mate Seeking Extraversion | 303 | 3.577 | 1.749 |
| Mate Seeking Conscientiousness | 303 | 1.988 | 1.709 |
| Mate Seeking Openness | 303 | 2.790 | 1.678 |
| Mate Seeking Neuroticism | 303 | 1.695 | 1.492 |
| Mate Seeking Warmth | 303 | 1.643 | 1.457 |
| Mate Seeking Competence | 303 | 1.286 | 1.540 |
| Disease Avoidance Extraversion | 303 | 2.775 | 1.853 |
| Disease Avoidance Conscientiousness | 303 | 2.239 | 1.745 |
| Disease Avoidance Openness | 303 | 2.736 | 1.843 |
| Disease Avoidance Neuroticism | 303 | 2.744 | 1.816 |
| Disease Avoidance Warmth | 303 | 1.794 | 1.653 |
| Disease Avoidance Competence | 303 | 1.456 | 1.570 |

## Trait Inference Correlations with Motive Prioritization

|  | *Prioritization FC NC* | *Prioritization FC C* | *Prioritization RS NC* | *Prioritization RS C* | *Warmth* | *Extraversion* | *Conscientiousness* | *Openness* | *Neuroticism* | *Competence* |
| --- | --- | --- | --- | --- | --- | --- | --- | --- | --- | --- |
| *Warmth* | 0.795 *(.003)* | 0.675 *(.023)* | 0.726 *(.011)* | 0.598 *(.052)* |  |  |  |  |  |  |
| *Extraversion* | -0.286 *(.394)* | -0.288 *(.390)* | -0.276 *(.412)* | -0.189 *(.577)* | -0.480 *(.135)* |  |  |  |  |  |
| *Conscientiousness* | 0.732 *(.010)* | 0.683 *(.020)* | 0.693 *(.018)* | 0.597 *(.052)* | 0.621 *(.041)* | -0.514 *(.106)* |  |  |  |  |
| *Openness* | -0.427 *(.190)* | -0.249 *(.461)* | -0.473 *(.141)* | -0.208 *(.540)* | -0.689 *(.019)* | 0.572 *(.066)* | -0.399 *(.225)* |  |  |  |
| *Neuroticism* | -0.461 *(.154)* | -0.188 *(.580)* | -0.595 *(.053)* | -0.229 *(.498)* | -0.160 *(.639)* | -0.351 *(.290)* | -0.225 *(.506)* | 0.230 *(.496)* |  |  |
| *Competence* | 0.653 *(.029)* | 0.601 *(.051)* | 0.560 *(.073)* | 0.520 *(.101)* | 0.762 *(.006)* | -0.352 *(.288)* | 0.786 *(.004)* | -0.593 *(.055)* | -0.180 *(.596)* |  |
| *Computed correlation used pearson-method with pairwise-deletion.* | | | | | | | | | | |

*Note: FC = Forced-Choice Measure; RS = Rating Scale Measure; NC = No Context Condition; C = Collapsed Context Conditions*
