# Supplementary material for: What Motives Do People Most Want to Know About When Meeting Another Person? An Investigation Into Prioritization of Information About Seven Fundamental Motives
Source: Pers Soc Psychol Bull. 2022 Jan 26;49(4):495–509. doi: 10.1177/01461672211069468 (PMC9989231; doi:10.1177/01461672211069468)
Supplement: sj-docx-2-psp-10.1177_01461672211069468 – Supplemental material for What Motives Do People Most Want to Know About When Meeting Another Person? An Investigation Into Prioritization of Information About Seven Fundamental Motives [file sj-docx-2-psp-10.1177_01461672211069468.docx]

Methods and Materials Document

What Motives do People Most Want to Know About When Meeting Another Person? An Investigation into Prioritization of Information about Seven Fundamental Motives

Table of Contents

[Demographics 3](#_Toc74841753)

[Motive Prioritization Prompt 5](#_Toc74841754)

[Forced-Choice Measure 7](#_Toc74841755)

[Rating Scale Measure 19](#_Toc74841756)

[Fundamental Social Motives Inventory 20](#_Toc74841757)

[BFI-44 25](#_Toc74841758)

[Motive Stability Measure 28](#_Toc74841759)

[Trait Inferences (Study 5) 29](#_Toc74841760)

[English Proficiency 29](#_Toc74841761)

[Trait Inference Measure 30](#_Toc74841762)

[Motive Prioritization Measure 32](#_Toc74841763)

[Attention Check 33](#_Toc74841764)

[Demographics 34](#_Toc74841765)

# Demographics

How old are you?

Please type your answer as a number. For example, someone who is 32 years old would type:

32

________________________________________________________________

What is your sex?

o Male

o Female

What is your father's level of education?

o No High School Diploma

o High School Diploma

o Some college, but no degree

o Associate or technical degree

o Bachelor's degree

o Graduate or professional degree

What is your mother's level of education?

o No High School Diploma

o High School Diploma

o Some college, but no degree

o Associate or technical degree

o Bachelor's degree

o Graduate or professional degree

During your childhood and adolescence, what was your approximate household income (or equivalent income in Canadian currency)?

o $0 - $25,000

o $25,000 - $50,000

o $50,000 - $75,000

o $75,000 - $100,000

o More than $100,000

What is your ethnic background? (Select all that apply)

▢ Caucasian (white)

▢ Hispanic/Latino

▢ African

▢ East Asian (e.g., Chinese, Japanese, or Korean)

▢ Southeast Asian (e.g., Indian)

▢ Middle Eastern (e.g., Iranian)

▢ Indigenous (e.g., Metis, First Nations, or Inuit)

▢ Other (please specify): ________________________________________________

# Motive Prioritization Prompt

*The text prompt below is consistent in all conditions. The bolded font is modified based on condition. See below for the specific modifications made in each condition.*

Imagine that you're about to **start a new job, but you haven't yet met your new boss. Because you know you'll be spending a lot of time working under this person’s direction**, you're probably curious to learn more about what he or she is like. What exactly are you most interested in learning about **your new boss?**

For each pair of characteristics presented in the next 55 questions, indicate which characteristic you would be most interested in learning about **your new boss** before meeting him or her.

Modifications in each condition

Control:

Imagine that you're about to meet a person.

What exactly are you most interested in learning about that person?

For each pair of characteristics presented in the next 55 questions, indicate which characteristic you would be most interested in learning about that person before meeting him or her.

Man/Woman:

Imagine that you're about to meet a man.

What exactly are you most interested in learning about that man?

For each pair of characteristics presented in the next 55 questions, indicate which characteristic you would be most interested in learning about that man before meeting him.

Dating (Casual/Life partner):

Imagine that you’re single and are looking for a casual sexual encounter. You’ve just met someone who you find incredibly attractive. After flirting with this person for a while, it seems that the two of you have sexual chemistry, and you will have the opportunity to hook up with this person for a one-night stand if you want to.

But do you want to? You might want to know a bit more about the characteristics of this person.

For each pair of characteristics presented in the next 55 questions, indicate which characteristic you would be most interested in learning about this person before deciding whether to hook up for a casual sexual encounter.

- *“single and looking for a casual sexual encounter”; “incredibly attractive”; “you will have the opportunity to hook up with this person for a one-night stand if you want to.*
- *“single and looking for a long-term life partner”; “might be a good match for you”; “you will have the opportunity to pursue a long-term romantic relationship if you want to.”*

Boss/Assistant/Co-worker:

Imagine that you're about to start a new job, but you haven't yet met your new boss. Because you know you'll be spending a lot of time working under this person’s direction, you're probably curious to learn more about what he or she is like. What exactly are you most interested in learning about your new boss?

For each pair of characteristics presented in the next 55 questions, indicate which characteristic you would be most interested in learning about your new boss before meeting him or her.

- *“new boss”; “you'll be spending a lot of time working under this person’s direction”*
- *“new assistant” (“you’ll be spending a lot of time directing this person’s work”*
- *“new co-worker”; “you’ll be spending a lot of time working alongside this person”*

Dark Alley (Young man/Elderly man/Young woman/Elderly woman):

Imagine that you’re visiting a big city far from home. Late one night, as you’re walking back to your hotel, you accidentally take a wrong turn into a dark alley. At first, you’re all alone in the alley, but then you notice a young man emerge from the shadows, approaching you.

For each pair of characteristics presented in the next 55 questions, indicate which characteristic you would be most interested in learning about this person.

# Forced-Choice Measure

*Prompt*: What would you be more interested in learning about **your new boss**?

*Forced-Choice Items:*

1) What would you be more interested in learning about your new boss?

- How motivated that person is to be close to their family and to attend to the needs of family members
- How motivated that person is to avoid being cheated on or dumped by a romantic partner

2) What would you be more interested in learning about your new boss?

- How motivated that person is to nurture and care for their children
- How motivated that person is to maintain a loyal and long-lasting romantic relationship

3) What would you be more interested in learning about your new boss?

- How motivated that person is to avoid being excluded or rejected by other people
- How motivated that person is to be independent and to spend time alone

4) What would you be more interested in learning about your new boss?

- How motivated that person is to nurture and care for their children
- How motivated that person is to achieve high status and positions of leadership

5) What would you be more interested in learning about your new boss?

- How motivated that person is to avoid dangerous people and risky situations
- How motivated that person is to be independent and to spend time alone

| Page Break |  |
| --- | --- |

6) What would you be more interested in learning about your new boss?

- How motivated that person is to achieve high status and positions of leadership
- How motivated that person is to avoid being cheated on or dumped by a romantic partner

7) What would you be more interested in learning about your new boss?

- How motivated that person is to avoid being cheated on or dumped by a romantic partner
- How motivated that person is to be independent and to spend time alone

8) What would you be more interested in learning about your new boss?

- How motivated that person is to be independent and to spend time alone
- How motivated that person is to be socially included and to be part of a group

9) What would you be more interested in learning about your new boss?

- How motivated that person is to seek out new romantic or sexual partners
- How motivated that person is to avoid being cheated on or dumped by a romantic partner

10) What would you be more interested in learning about your new boss?

- How motivated that person is to avoid infectious diseases and people who carry diseases
- How motivated that person is to avoid being cheated on or dumped by a romantic partner

| Page Break |  |
| --- | --- |

11) What would you be more interested in learning about your new boss?

- How motivated that person is to seek out new romantic or sexual partners
- How motivated that person is to avoid infectious diseases and people who carry diseases

12) What would you be more interested in learning about your new boss?

- How motivated that person is to maintain a loyal and long-lasting romantic relationship
- How motivated that person is to seek out new romantic or sexual partners

13) What would you be more interested in learning about your new boss?

- How motivated that person is to be close to their family and to attend to the needs of family members
- How motivated that person is to avoid being excluded or rejected by other people

14) What would you be more interested in learning about your new boss?

- How motivated that person is to avoid dangerous people and risky situations
- How motivated that person is to seek out new romantic or sexual partners

15) What would you be more interested in learning about your new boss?

- How motivated that person is to seek out new romantic or sexual partners
- How motivated that person is to be socially included and to be part of a group

| Page Break |  |
| --- | --- |

16) What would you be more interested in learning about your new boss?

- How motivated that person is to maintain a loyal and long-lasting romantic relationship
- How motivated that person is to avoid dangerous people and risky situations

17) What would you be more interested in learning about your new boss?

- How motivated that person is to be independent and to spend time alone
- How motivated that person is to achieve high status and positions of leadership

18) What would you be more interested in learning about your new boss?

- How motivated that person is to maintain a loyal and long-lasting romantic relationship
- How motivated that person is to be independent and to spend time alone

19) What would you be more interested in learning about your new boss?

- How motivated that person is to be close to their family and to attend to the needs of family members
- How motivated that person is to achieve high status and positions of leadership

20) What would you be more interested in learning about your new boss?

- How motivated that person is to maintain a loyal and long-lasting romantic relationship
- How motivated that person is to be socially included and to be part of a group

| Page Break |  |
| --- | --- |

21) What would you be more interested in learning about your new boss?

- How motivated that person is to be independent and to spend time alone
- How motivated that person is to avoid infectious diseases and people who carry diseases

22) What would you be more interested in learning about your new boss?

- How motivated that person is to avoid being excluded or rejected by other people
- How motivated that person is to nurture and care for their children

23) What would you be more interested in learning about your new boss?

- How motivated that person is to nurture and care for their children
- How motivated that person is to be socially included and to be part of a group

24) What would you be more interested in learning about your new boss?

- How motivated that person is to be close to their family and to attend to the needs of family members
- How motivated that person is to be independent and to spend time alone

25) What would you be more interested in learning about your new boss?

- How motivated that person is to be socially included and to be part of a group
- How motivated that person is to avoid dangerous people and risky situations

| Page Break |  |
| --- | --- |

26) What would you be more interested in learning about your new boss?

- How motivated that person is to be socially included and to be part of a group
- How motivated that person is to achieve high status and positions of leadership

27) What would you be more interested in learning about your new boss?

- How motivated that person is to nurture and care for their children
- How motivated that person is to avoid dangerous people and risky situations

28) What would you be more interested in learning about your new boss?

- How motivated that person is to seek out new romantic or sexual partners
- How motivated that person is to nurture and care for their children

29) What would you be more interested in learning about your new boss?

- How motivated that person is to avoid infectious diseases and people who carry diseases
- How motivated that person is to nurture and care for their children

30) What would you be more interested in learning about your new boss?

- How motivated that person is to avoid dangerous people and risky situations
- How motivated that person is to be close to their family and to attend to the needs of family members

| Page Break |  |
| --- | --- |

31) What would you be more interested in learning about your new boss?

- How motivated that person is to be close to their family and to attend to the needs of family members
- How motivated that person is to maintain a loyal and long-lasting romantic relationship

32) What would you be more interested in learning about your new boss?

- How motivated that person is to avoid being excluded or rejected by other people
- How motivated that person is to avoid infectious diseases and people who carry diseases

33) What would you be more interested in learning about your new boss?

- How motivated that person is to maintain a loyal and long-lasting romantic relationship
- How motivated that person is to avoid being excluded or rejected by other people

34) What would you be more interested in learning about your new boss?

- How motivated that person is to avoid dangerous people and risky situations
- How motivated that person is to avoid being cheated on or dumped by a romantic partner

35) What would you be more interested in learning about your new boss?

- How motivated that person is to avoid being excluded or rejected by other people
- How motivated that person is to seek out new romantic or sexual partners

| Page Break |  |
| --- | --- |

36) What would you be more interested in learning about your new boss?

- How motivated that person is to be close to their family and to attend to the needs of family members
- How motivated that person is to seek out new romantic or sexual partners

37) What would you be more interested in learning about your new boss?

- How motivated that person is to nurture and care for their children
- How motivated that person is to be independent and to spend time alone

38) What would you be more interested in learning about your new boss?

- How motivated that person is to avoid infectious diseases and people who carry diseases
- How motivated that person is to be socially included and to be part of a group

39) What would you be more interested in learning about your new boss?

- How motivated that person is to avoid being excluded or rejected by other people
- How motivated that person is to achieve high status and positions of leadership

40) What would you be more interested in learning about your new boss?

- How motivated that person is to avoid being excluded or rejected by other people
- How motivated that person is to be socially included and to be part of a group

| Page Break |  |
| --- | --- |

41) What would you be more interested in learning about your new boss?

- How motivated that person is to achieve high status and positions of leadership
- How motivated that person is to avoid dangerous people and risky situations

42) What would you be more interested in learning about your new boss?

- How motivated that person is to achieve high status and positions of leadership
- How motivated that person is to seek out new romantic or sexual partners

43) What would you be more interested in learning about your new boss?

- How motivated that person is to be close to their family and to attend to the needs of family members
- How motivated that person is to be socially included and to be part of a group

44) What would you be more interested in learning about your new boss?

- How motivated that person is to be close to their family and to attend to the needs of family members
- How motivated that person is to nurture and care for their children

45) What would you be more interested in learning about your new boss?

- How motivated that person is to avoid being cheated on or dumped by a romantic partner
- How motivated that person is to maintain a loyal and long-lasting romantic relationship

| Page Break |  |
| --- | --- |

46) What would you be more interested in learning about your new boss?

- How motivated that person is to avoid being cheated on or dumped by a romantic partner
- How motivated that person is to be socially included and to be part of a group

47) What would you be more interested in learning about your new boss?

- How motivated that person is to avoid infectious diseases and people who carry diseases
- How motivated that person is to achieve high status and positions of leadership

48) What would you be more interested in learning about your new boss?

- How motivated that person is to avoid being excluded or rejected by other people
- How motivated that person is to avoid dangerous people and risky situations

49) What would you be more interested in learning about your new boss?

- How motivated that person is to maintain a loyal and long-lasting romantic relationship
- How motivated that person is to achieve high status and positions of leadership

50) What would you be more interested in learning about your new boss?

- How motivated that person is to avoid infectious diseases and people who carry diseases
- How motivated that person is to maintain a loyal and long-lasting romantic relationship

| Page Break |  |
| --- | --- |

51) What would you be more interested in learning about your new boss?

- How motivated that person is to avoid infectious diseases and people who carry diseases
- How motivated that person is to avoid dangerous people and risky situations

52) What would you be more interested in learning about your new boss?

- How motivated that person is to avoid being cheated on or dumped by a romantic partner
- How motivated that person is to avoid being excluded or rejected by other people

53) What would you be more interested in learning about your new boss?

- How motivated that person is to avoid infectious diseases and people who carry diseases
- How motivated that person is to be close to their family and to attend to the needs of family members

54) What would you be more interested in learning about your new boss?

- How motivated that person is to avoid being cheated on or dumped by a romantic partner
- How motivated that person is to nurture and care for their children

55) What would you be more interested in learning about your new boss?

- How motivated that person is to be independent and to spend time alone
- How motivated that person is to seek out new romantic or sexual partners

# Rating Scale Measure

Next, please indicate how interested you would be in learning each of the following things about **your new boss**.

|  |  |
| --- | --- |
| How motivated that person is to be close to their family and to attend to the needs of family members. | ▼ Very interested ... Very uninterested |
| How motivated that person is to avoid being excluded or rejected by other people. | ▼ Very interested ... Very uninterested |
| How motivated that person is to avoid dangerous people and risky situations. | ▼ Very interested ... Very uninterested |
| How motivated that person is to nurture and care for their children. | ▼ Very interested ... Very uninterested |
| How motivated that person is to be independent and to spend time alone. | ▼ Very interested ... Very uninterested |
| How motivated that person is to avoid being cheated on or dumped by a romantic partner. | ▼ Very interested ... Very uninterested |
| How motivated that person is to avoid infectious diseases and people who carry diseases. | ▼ Very interested ... Very uninterested |
| How motivated that person is to achieve high status and positions of leadership. | ▼ Very interested ... Very uninterested |
| How motivated that person is to be socially included and to be part of a group. | ▼ Very interested ... Very uninterested |
| How motivated that person is to seek out new romantic or sexual partners. | ▼ Very interested ... Very uninterested |
| How motivated that person is to maintain a loyal and long-lasting romantic relationship. | ▼ Very interested ... Very uninterested |

# Fundamental Social Motives Inventory

Do you have children?

- Yes
- No

What is your current relationship status?

- Married
- In a committed relationship
- Dating one person
- Dating several people
- Single
- Other (please specify): ________________________________________________

*Based on participant responses to the above questions concerning children and relationship status, we presented a selection of the FSMI subscales displayed below. Participants who did not indicate they had children were not presented with the Kin Care (Children) subscale. Participants who did not indicate they were in a relationship were not presented with the Mate Retention subscales (General & Breakup Concern)*

| 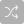 |
| --- |

Please indicate the extent to which you agree or disagree with the following statements.

|  | Strongly disagree | Disagree | Disagree somewhat | Neither agree nor disagree | Agree somewhat | Agree | Strongly agree |
| --- | --- | --- | --- | --- | --- | --- | --- |
| I spend a lot of time thinking about ways to meet possible dating partners. |  |  |  |  |  |  |  |
| I am interested in finding a new romantic/sexual partner. |  |  |  |  |  |  |  |
| I am not interested in meeting people to flirt with or date. |  |  |  |  |  |  |  |
| Starting a new romantic/sexual relationship is not a high priority for me. |  |  |  |  |  |  |  |
| I rarely think about finding a romantic or sexual partner. |  |  |  |  |  |  |  |
| I would like to find a new romantic/sexual partner soon. |  |  |  |  |  |  |  |
| It’s important to me that other people look up to me. |  |  |  |  |  |  |  |
| I want to be in a position of leadership. |  |  |  |  |  |  |  |
| It's important to me that others respect my rank or position. |  |  |  |  |  |  |  |
| I do things to ensure that I don’t lose the status I have. |  |  |  |  |  |  |  |
| I do not like being at the bottom of a hierarchy. |  |  |  |  |  |  |  |
| I do not worry very much about losing status. |  |  |  |  |  |  |  |
| I would be extremely hurt if a friend excluded me. |  |  |  |  |  |  |  |
| It would be a big deal to me if a group excluded me. |  |  |  |  |  |  |  |
| It bothers me when groups of people I know do things without me. |  |  |  |  |  |  |  |
| I worry about being rejected. |  |  |  |  |  |  |  |
| I often wonder whether I am being excluded. |  |  |  |  |  |  |  |
| I often think about whether other people accept me. |  |  |  |  |  |  |  |
| I would prefer to spend time alone than to be surrounded by other people. |  |  |  |  |  |  |  |
| I like to be alone even if I might lose some friends because of it. |  |  |  |  |  |  |  |
| Being apart from my friends for long periods of time does not bother me. |  |  |  |  |  |  |  |
| I don't mind being by myself for long periods of time. |  |  |  |  |  |  |  |
| Having time alone is extremely important to me. |  |  |  |  |  |  |  |
| I like to be by myself. |  |  |  |  |  |  |  |
| Being part of a group is important to me. |  |  |  |  |  |  |  |
| I enjoy working with a group to accomplish a goal. |  |  |  |  |  |  |  |
| I like being part of a team. |  |  |  |  |  |  |  |
| Working in a group is usually more trouble than it's worth. |  |  |  |  |  |  |  |
| When I’m in a group, I do things to help the group stay together. |  |  |  |  |  |  |  |
| Getting along with the people around me is a high priority. |  |  |  |  |  |  |  |
| Caring for family members is important to me. |  |  |  |  |  |  |  |
| Having close ties to my family is not very important to me. |  |  |  |  |  |  |  |
| I am not very interested in helping my family members. |  |  |  |  |  |  |  |
| I would rather not spend time with family members. |  |  |  |  |  |  |  |
| Being close to my family members is extremely important to me. |  |  |  |  |  |  |  |
| It is extremely important to me to have good relationships with my family members. |  |  |  |  |  |  |  |
| I think a lot about how to stay safe from dangerous people. |  |  |  |  |  |  |  |
| I am motivated to keep myself safe from others. |  |  |  |  |  |  |  |
| I do not worry about keeping myself safe from others. |  |  |  |  |  |  |  |
| I worry about dangerous people. |  |  |  |  |  |  |  |
| I think about how to protect myself from dangerous people. |  |  |  |  |  |  |  |
| I am motivated to protect myself from dangerous others. |  |  |  |  |  |  |  |
| I avoid places and people that might carry diseases. |  |  |  |  |  |  |  |
| I avoid people who might have a contagious illness. |  |  |  |  |  |  |  |
| I worry about catching colds and flu from too much contact with other people. |  |  |  |  |  |  |  |
| I do not worry very much about getting germs from others. |  |  |  |  |  |  |  |
| When someone near me is sick, it doesn’t bother me very much. |  |  |  |  |  |  |  |
| I don't mind being around people who are sick. |  |  |  |  |  |  |  |
| It is important to me that my partner is sexually loyal to me. |  |  |  |  |  |  |  |
| It is important to me that my partner is emotionally loyal to me. |  |  |  |  |  |  |  |
| I do not spend much time and energy doing things to keep my partner invested in our relationship. |  |  |  |  |  |  |  |
| It would not be that big a deal to me if my partner and I broke up. |  |  |  |  |  |  |  |
| If others were romantically interested in my partner, it would not bother me very much. |  |  |  |  |  |  |  |
| If my partner were to have romantic or sexual relationships with others, that would be OK with me. |  |  |  |  |  |  |  |
| I often think about whether my partner will leave me. |  |  |  |  |  |  |  |
| I worry about others stealing my romantic/sexual partner. |  |  |  |  |  |  |  |
| I worry that my romantic/sexual partner might leave me. |  |  |  |  |  |  |  |
| I wonder if my partner will leave me for someone else. |  |  |  |  |  |  |  |
| I worry that other people are interested in my romantic/sexual partner. |  |  |  |  |  |  |  |
| I am worried that my partner and I might break up. |  |  |  |  |  |  |  |
| I help take care of my children. |  |  |  |  |  |  |  |
| I like to spend time with my children. |  |  |  |  |  |  |  |
| Taking care of my children is not a high priority for me right now. |  |  |  |  |  |  |  |
| I often think about how I could stop bad things from happening to my children. |  |  |  |  |  |  |  |
| I rarely think about protecting my children. |  |  |  |  |  |  |  |
| Providing for my children is important to me. |  |  |  |  |  |  |  |

# BFI-44

Here are a number of characteristics that may or may not apply to you. For example, do you agree that you are someone who likes to spend time with others? Please indicate the extent to which you agree or disagree with each statement. Answer quickly. Please do not skip any items.
 
**I see myself as someone who...**

|  | Disagree strongly | Disagree a little | Neither agree nor disagree | Agree a little | Agree strongly |
| --- | --- | --- | --- | --- | --- |
| Is talkative |  |  |  |  |  |
| Tends to find fault with others |  |  |  |  |  |
| Does a thorough job |  |  |  |  |  |
| Is depressed, blue |  |  |  |  |  |
| Is original, comes up with new ideas |  |  |  |  |  |
| Is reserved |  |  |  |  |  |
| Is helpful and unselfish with others |  |  |  |  |  |
| Can be somewhat careless |  |  |  |  |  |
| Is relaxed, handles stress well |  |  |  |  |  |
| Is curious about many different things |  |  |  |  |  |
| Is full of energy |  |  |  |  |  |
| Starts quarrels with others |  |  |  |  |  |
| Is a reliable worker |  |  |  |  |  |
| Can be tense |  |  |  |  |  |
| Is ingenious, a deep thinker |  |  |  |  |  |
| Generates a lot of enthusiasm |  |  |  |  |  |
| Has a forgiving nature |  |  |  |  |  |
| Tends to be disorganized |  |  |  |  |  |
| Worries a lot |  |  |  |  |  |
| Has an active imagination |  |  |  |  |  |
| Tends to be quiet |  |  |  |  |  |
| Is generally trusting |  |  |  |  |  |
| Tends to be lazy |  |  |  |  |  |
| Is emotionally stable, not easily upset |  |  |  |  |  |
| Is inventive |  |  |  |  |  |
| Has an assertive personality |  |  |  |  |  |
| Can be cold and aloof |  |  |  |  |  |
| Perseveres until the task is finished |  |  |  |  |  |
| Can be moody |  |  |  |  |  |
| Values artistic, aesthetic experiences |  |  |  |  |  |
| Is sometimes shy, inhibited |  |  |  |  |  |
| Is considerate and kind to almost everyone |  |  |  |  |  |
| Respond to this item with "Agree a little" |  |  |  |  |  |
| Does things efficiently |  |  |  |  |  |
| Remains calm in tense situations |  |  |  |  |  |
| Prefers work that is routine |  |  |  |  |  |
| Is outgoing, sociable |  |  |  |  |  |
| Is sometimes rude to others |  |  |  |  |  |
| Makes plans and follows through with them |  |  |  |  |  |
| Gets nervous easily |  |  |  |  |  |
| Likes to reflect, play with ideas |  |  |  |  |  |
| Has few artistic interests |  |  |  |  |  |
| Likes to cooperate with others |  |  |  |  |  |
| Is easily distracted |  |  |  |  |  |
| Is sophisticated in art, music, or literature |  |  |  |  |  |

# Motive Stability Measure

The next 11 items identify specific aspects of individuals’ personalities.  For each of these personality characteristics, please indicate your belief about how stable, enduring, and resistant-to-change it is.

|  | Very unstable | Unstable | Somewhat unstable | Neutral | Somewhat stable | Stable | Very stable |
| --- | --- | --- | --- | --- | --- | --- | --- |
| How motivated a person is to be close to their family and to attend to the needs of family members |  |  |  |  |  |  |  |
| How motivated a person is to avoid being excluded or rejected by other people |  |  |  |  |  |  |  |
| How motivated a person is to avoid dangerous people and risky situations |  |  |  |  |  |  |  |
| How motivated a person is to nurture and care for their children |  |  |  |  |  |  |  |
| How motivated a person is to be independent and to spend time alone |  |  |  |  |  |  |  |
| How motivated a person is to avoid being cheated on or dumped by a romantic partner |  |  |  |  |  |  |  |
| How motivated a person is to avoid infectious diseases and people who carry diseases |  |  |  |  |  |  |  |
| How motivated a person is to achieve high status and positions of leadership |  |  |  |  |  |  |  |
| How motivated a person is to be socially included and to be part of a group |  |  |  |  |  |  |  |
| How motivated a person is to seek out new romantic or sexual partners |  |  |  |  |  |  |  |
| How motivated a person is to maintain a loyal and long-lasting romantic relationship |  |  |  |  |  |  |  |

# Trait Inferences (Study 5)

## English Proficiency

Choose the correct answer.
 
The hospital is now discounting 10 standard operations and _____ , ranging from having a baby and treating a cataract to undergoing a heart bypass.

- procedures
- equipment
- visits
- medication

Choose the correct answer.

 The number of girls in school and women in parliaments has risen, and their overall access to contraception has improved in the past decade, _____ new report.

- according a
- published in a
- according to a
- stated in a

Choose the correct answer.

 By far the most noticeable blemishes on the surface of the Sun _____ sunspots.

- the
- are
- that are
- in the

## Trait Inference Measure

*Note: The order of the response options were counterbalanced so that half of the sample responded to “warm and sympathetic” first and one half responded to “extroverted and enthusiastic” first. The order of motives was randomized.*

The purpose of this questionnaire is to get a sense of whether it is (or isn't) possible to draw conclusions about a person's personality traits based on a piece of information about that person's motives.

You'll be asked to respond to each piece of information on a 7-point rating scale (1=not at all informative; 7=very informative)

For the following 7 items, imagine that the only thing that you know about a person is:

*Kin Care*: **The person has a very high motivation to be close to their family and to attend to the needs of family members.**

*Mate Retention*: **The person has a very high motivation to maintain a loyal and long-lasting romantic relationship.**

*Affiliation*: **The person has a very high motivation to be socially included and to be part of a group.**

*Self-Protection:* **The person has a very high motivation to avoid dangerous people and risky situations.**

*Disease Avoidance*: **The person has a very high motivation to avoid infectious diseases and people who carry diseases.**

*Status*: **The person has a very high motivation to achieve high status and positions of leadership.**

*Mate Seeking*: **The person has a very high motivation to seek out new romantic or sexual partners.**

Now, please rate the extent to which that specific piece of information is actually informative about whether the person is (or isn't)...

|  | Not at all informative | 2 | 3 | 4 | 5 | 6 | Very informative |
| --- | --- | --- | --- | --- | --- | --- | --- |
| ... warm and sympathetic |  |  |  |  |  |  |  |
| ... dependable and self-disciplined |  |  |  |  |  |  |  |
| ... capable and competent |  |  |  |  |  |  |  |
| ... honest and trustworthy |  |  |  |  |  |  |  |
| ... anxious and easily upset |  |  |  |  |  |  |  |
| ... complex and open to new experiences |  |  |  |  |  |  |  |
| ... extroverted and enthusiastic |  |  |  |  |  |  |  |

## Motive Prioritization Measure

Imagine that you're about to meet a person. Please indicate how interested you would be in learning each of the following things about that person.


(1 = very uninterested and 7 = very interested)

Indicate how interested you would be in learning how motivated that person is to...

|  | Very uninterested (1) | 2 (2) | 3 (3) | Neutral (4) | 5 (5) | 6 (6) | Very interested (7) |
| --- | --- | --- | --- | --- | --- | --- | --- |
| ...be close to their family and to attend to the needs of family members (2) |  |  |  |  |  |  |  |
| ...avoid dangerous people and risky situations (3) |  |  |  |  |  |  |  |
| ...avoid infectious diseases and people who carry diseases (4) |  |  |  |  |  |  |  |
| ...achieve high status and positions of leadership (5) |  |  |  |  |  |  |  |
| ...be socially included and to be part of a group (6) |  |  |  |  |  |  |  |
| ...seek out new romantic or sexual partners (7) |  |  |  |  |  |  |  |
| ...maintain a loyal and long-lasting romantic relationship (8) |  |  |  |  |  |  |  |

## Attention Check

Research surveys often recruit hundreds of participants, which makes it very difficult to distinguish participants who thoughtfully completed the survey from participants who simply clicked their way through. We ask you to answer this question according to the following instructions, so we can be confident that you were one of those thoughtful participants! For the question below, please select the "Other" option. In the "Other" text box, please type the following word exactly as it appears here: Tolstoy 

- Kierkegaard (1)
- Camus (2)
- Thoreau (3)
- Emerson (4)
- Other (5) ________________________________________________

## Demographics

Age (please use numbers to indicate your age)

▼ Under 18 (1) ... 85 or older (9)

Gender

- Male (1)
- Female (2)
- Other (3)

Which ethnic group do you most identify with?

- White (1)
- Black or African American (2)
- American Indian or Alaska Native (3)
- Asian (4)
- Middle Eastern (5)
- Hispanic or Latino (6)
- Other - please specify (7) ________________________________________________

Please indicate what your household annual income is

▼ $0 - $19,000 (1) ... Greater than $220,000 (8)

What is your political orientation?

- Very Liberal (1)
- 2 (2)
- 3 (3)
- 4 (4)
- 5 (5)
- 6 (6)
- Very Conservative (7)

What is your religion?

- Christian (1)
- Hindu (2)
- Buddhist (3)
- Jewish (4)
- Athiest (5)
- Agnostic (6)
- Muslim (7)
- Non-religious (8)
- Sikh (9)
- Jain (10)
- Other - please specify (11) ________________________________________________

Please rate the following statements:

|  | Not at all | 2 | 3 | 4 | 5 | 6 | Very |
| --- | --- | --- | --- | --- | --- | --- | --- |
| How important is God in your life? (1) |  |  |  |  |  |  |  |
| How religious are you? (2) |  |  |  |  |  |  |  |
| How spiritual are you? (3) |  |  |  |  |  |  |  |

What is your highest educational degree? If you are currently in school please select your highest *completed* level of education.

- Less than high school
- High school graduate
- Some college, but no degree
- 2 year degree (e.g. associate's degree)
- 4 year degree (e.g. bachelor's degree)
- Postgraduate degree (e.g. MA, PhD, MD)
